# ChatGPT Research Group for Optimizing the Crystallinity of MOFs and COFs

**DOI:** 10.1021/acscentsci.3c01087

**Published:** 2023-11-10

**Authors:** Zhiling Zheng, Oufan Zhang, Ha L. Nguyen, Nakul Rampal, Ali H. Alawadhi, Zichao Rong, Teresa Head-Gordon, Christian Borgs, Jennifer T. Chayes, Omar M. Yaghi

**Affiliations:** † Department of Chemistry, University of California, Berkeley, California 94720, United States; ‡ Kavli Energy Nanoscience Institute, University of California, Berkeley, California 94720, United States; § Bakar Institute of Digital Materials for the Planet, College of Computing, Data Science, and Society, University of California, Berkeley, California 94720, United States; ∇ Kenneth S. Pitzer Center for Theoretical Chemistry, University of California, Berkeley, California 94720, United States; ¶ Department of Chemical and Biomolecular Engineering, University of California, Berkeley, California 94720, United States; ° Department of Bioengineering, University of California, Berkeley, California 94720, United States; ◊ Department of Electrical Engineering and Computer Sciences, University of California, Berkeley, California 94720, United States; • Department of Mathematics, University of California, Berkeley, California 94720, United States; □ Department of Statistics, University of California, Berkeley, California 94720, United States; ■ School of Information, University of California, Berkeley, California 94720, United States; ∥ KACST−UC Berkeley Center of Excellence for Nanomaterials for Clean Energy Applications, King Abdulaziz City for Science and Technology, Riyadh 11442, Saudi Arabia

## Abstract

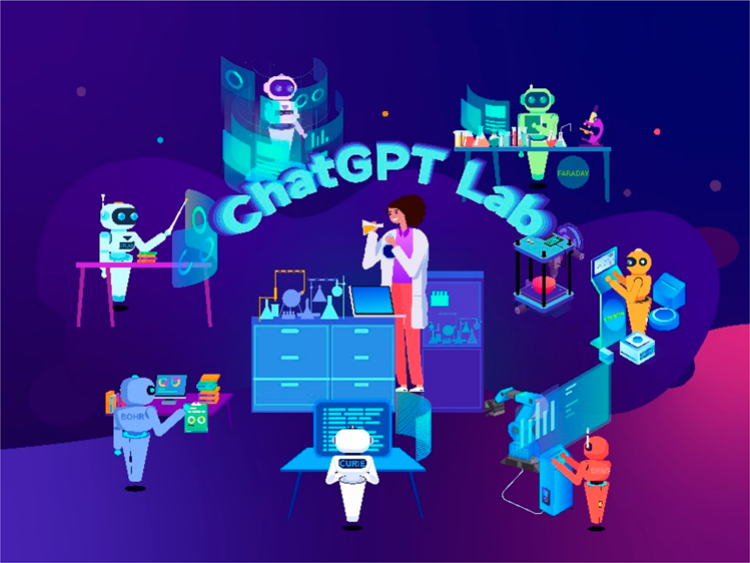

We leveraged the power of ChatGPT and Bayesian optimization in the development of a multi-AI-driven system, backed by seven large language model-based assistants and equipped with machine learning algorithms, that seamlessly orchestrates a multitude of research aspects in a chemistry laboratory (termed the ChatGPT Research Group). Our approach accelerated the discovery of optimal microwave synthesis conditions, enhancing the crystallinity of MOF-321, MOF-322, and COF-323 and achieving the desired porosity and water capacity. In this system, human researchers gained assistance from these diverse AI collaborators, each with a unique role within the laboratory environment, spanning strategy planning, literature search, coding, robotic operation, labware design, safety inspection, and data analysis. Such a comprehensive approach enables a single researcher working in concert with AI to achieve productivity levels analogous to those of an entire traditional scientific team. Furthermore, by reducing human biases in screening experimental conditions and deftly balancing the exploration and exploitation of synthesis parameters, our Bayesian search approach precisely zeroed in on optimal synthesis conditions from a pool of 6 million within a significantly shortened time scale. This work serves as a compelling proof of concept for an AI-driven revolution in the chemistry laboratory, painting a future where AI becomes an efficient collaborator, liberating us from routine tasks to focus on pushing the boundaries of innovation.

## Abstract 2

Leveraging ChatGPT and Bayesian optimization, this study introduces a multi-AI-driven chemistry lab system by merging AI with human expertise, expediting optimal synthesis condition discovery for MOFs and COFs.

## Introduction

Rapid advances in artificial intelligence (AI) inevitably will reshape chemistry and what chemists do in the laboratory.^1−5^ In particular, the recent development of large language models (LLMs) and machine learning (ML) algorithms will provide chemists with robust new means to address material discovery challenges.^2,6−17^ However, the complexity of laboratory routines often results in AI participation in isolated parts of the research process (e.g., predictive modeling, literature mining, robotic operations, and data analysis), resulting in a fragmented workflow that requires extensive human intervention in terms of coding, which is less accessible to chemists with limited programming experience. Bridging this gap demands innovative strategies that harness the AI’s real-time learning and self-instruction capabilities toward more comprehensive research automation.^18−20^

**Figure 1:**
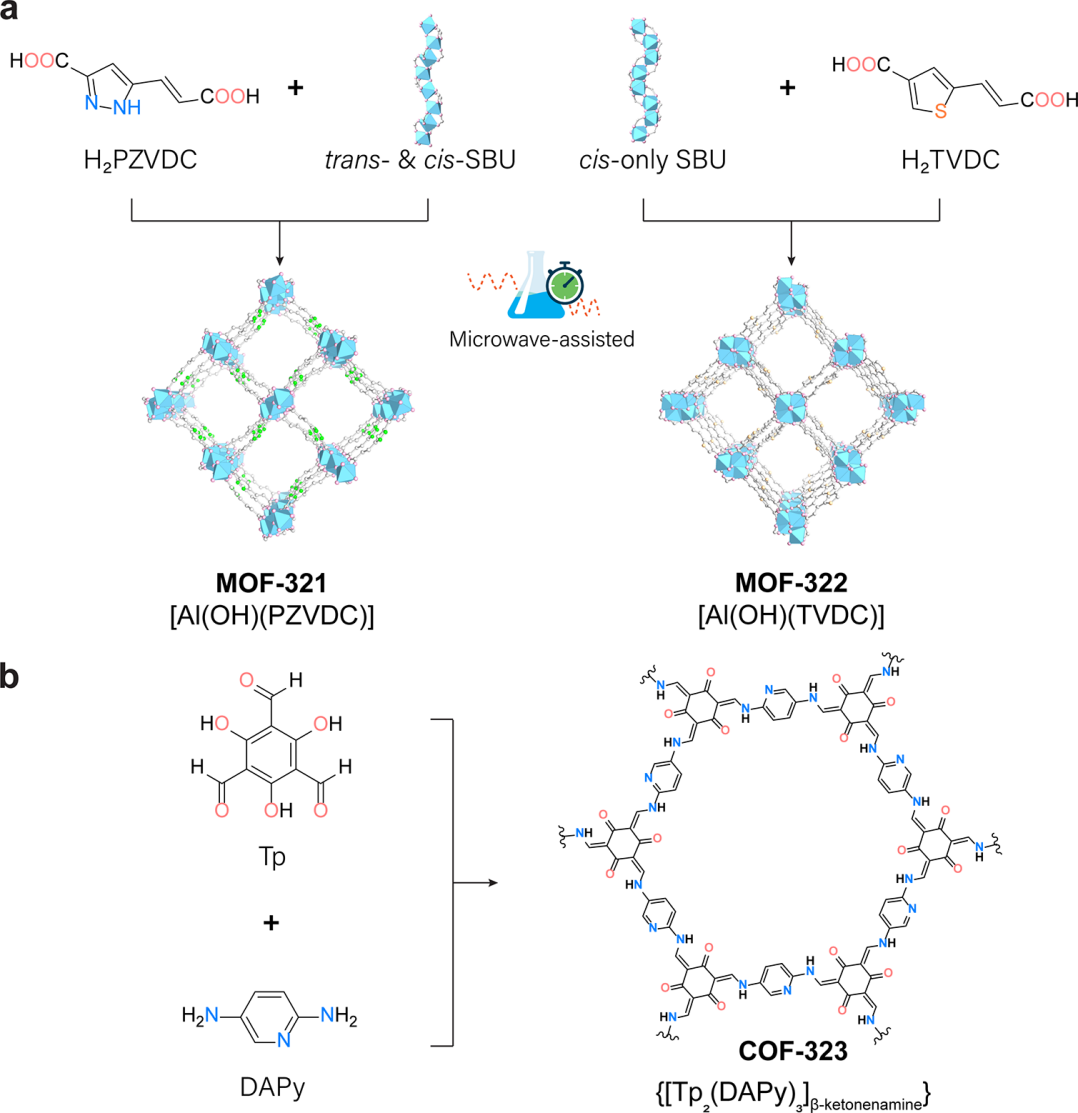
Microwave-assisted green synthesis of the crystalline compounds MOF-321 (MOF-LA2-1), MOF-322, and COF-323. (a) Comparison of the framework structures of rod MOFs, MOF-321 (left), and MOF-322 (right), highlighting their distinct organic linkers and aluminum rod SBU, which influence their optimal synthesis conditions. Color code: Al, blue octahedron; C, gray; N, green; S, yellow; O, pink. Hydrogen atoms are omitted for clarity. (b) Chemical structure of COF-323, [Tp_2_(DAPy)_3_]_β-ketonenamine_, formed by reticulating 1,3,5-triformylphloroglucinol (Tp) and 2,5-diaminopyridine (DAPy).

Herein, we introduce a protocol architecture leveraging LLMs, specifically ChatGPT powered by the GPT-4 model,^21^ to assemble a team of seven distinct AI research assistants, each specialized in different aspects of the research process.^21−28^ This approach seamlessly integrates these virtual collaborators, allowing humans to delegate a wide array of research tasks from literature review and code writing to laboratory operations and data interpretation. To demonstrate this strategy, we applied it to optimize the synthesis of reticular materials such as metal–organic frameworks (MOFs) and covalent organic frameworks (COFs) using Bayesian optimization^10,29,30^ (BO) algorithms. Specifically, we focused on MOF-321 [Al(OH)(PZVDC)], MOF-322 [Al(OH)(TVDC)] (Figure 1a), and COF-323 (Figure 1b), enabling the AI to initiate the discovery of optimal, previously unreported, microwave-assisted green synthesis conditions with no previous knowledge of such conditions.^31,32^ This multi-AI-agent approach’s strength lies in its design, which enables it to (i) accept human instructions in conversational language, eliminating the need for coding experience, (ii) promote task specialization, minimizing potential confusion from a singular LLM handling multiple roles, and (iii) incorporate a real-time, text-based feedback mechanism, allowing the AI to adapt to evolving project details. Furthermore, the ML algorithms incorporated into this system ensure that both human bias and hallucinations from LLM-based assistants can be reduced. This approach not only augments research efficiency but also redefines the traditional research paradigm. It enables a single researcher to match the productivity of a team of experts, thus providing a promising pathway toward fully automated research, wherein humans and AI synergistically collaborate to drive scientific discovery and innovation.

## Results and Discussion

**Figure 2:**
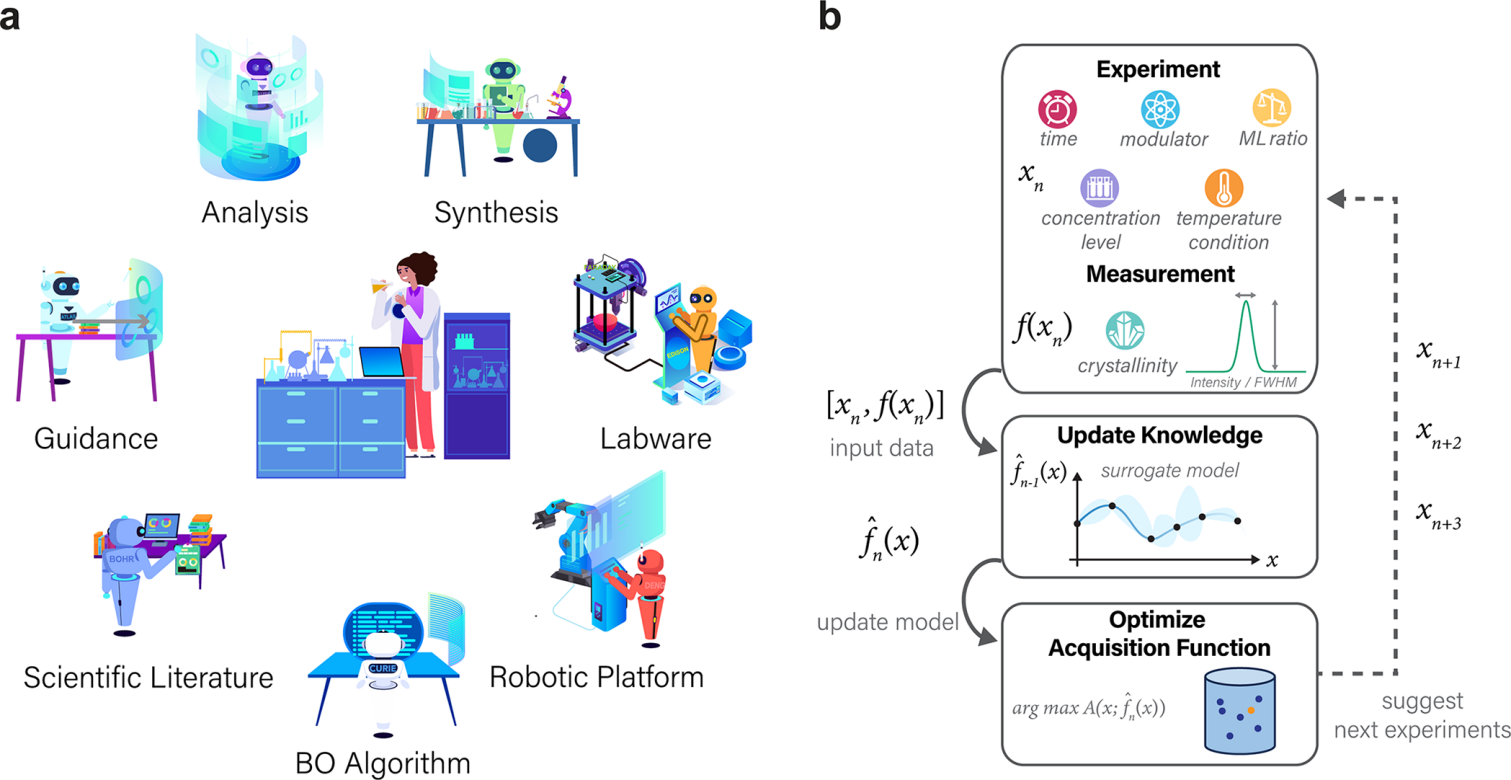
ChatGPT research group. (a) Assigned roles of seven ChatGPT-based assistants, each collaborating to assist human researchers and contributing to diverse research tasks at different stages of the synthesis optimization. (b) Flowchart outlining the closed-loop Bayesian optimization process. Each iteration involves three proposed experiments, their execution, data analysis, and integration of the new data into the existing data set to update the surrogate model, upon which the acquisition function is optimized to suggest the next three experiments.

Our AI-assisted strategy for optimizing the green synthesis of crystalline compounds integrates two critical elements: the LLM-based assistants and the ML algorithm (Figure 2a and b). The former is designed to facilitate routine laboratory work, aiding researchers in various time-consuming tasks by leveraging extensive domain knowledge (Figure 2a). In contrast, the latter aims to iteratively suggest new experimental conditions based on existing data, utilizing a Bayesian optimization search that intelligently accelerates a trial-and-error approach (Figure 2b), as this algorithm is known for finding the global optimum of a black box objective function *f*(*x*) in a minimum number of steps^33^ and has shown previous success in property prediction and synthesis optimization for material discovery.^10,14,34−37^

At the outset of this study, to expedite the experimental cycle, we opted for microwave synthesis owing to its reduced reaction time.^38,39^ An iteration comprising three experiments could be run and analyzed within 1–3 h before advancing to the next iteration. The programmability of the microwave system allowed for precise presetting of reaction parameters, facilitating the sequential execution of multiple reactions with minimal human intervention. Besides, microwave synthesis also facilitates the transferability of optimal stoichiometry conditions to conventional and solvothermal synthesis methodologies, enhancing its adaptability.^32,40^ For MOFs, an additional motivation is our interest in green synthesis, as the resulting MOFs have potential applications as sorbents for atmospheric water harvesting.^41−43^ Avoiding toxic solvents such as DMF ensures that the synthesis process is environmentally friendly and cost-effective.^44−46^

We have previously shown that an AI assistant, powered by ChatGPT, can achieve automation in various tasks such as extracting synthesis conditions from literature papers, code generation, research planning, and procedural guidance.^47,48^ Here, we further integrate these abilities to create a dynamic and efficient chemistry laboratory ecosystem that can assist researchers across various tasks, effectively extending its applications to building machine learning models, operating robotic platforms for synthesis preparation, designing 3D printed labware, and more (Supporting Information, Figure S1). These tasks, taken together, represent what we term the ChatGPT Research Group for materials discovery that spans from the initial stages to the end.

Through prompt engineering strategies (Supporting Information, Sections S1 and 2), we created tailored prompts for each of the seven AI assistants (Supporting Information, Figures S2–17), enabling them to focus on their designated tasks and maintain their specialization.^20,25,48−50^ This strategy prevents a single LLM-based assistant from handling a multitude of tasks, which could dilute its efficiency.

Furthermore, this framework allows individual assistants to recall previous human interactions as memory and adapt based on human feedback regarding the task performance. As a result, in our architecture, the workload of human researchers was substantially reduced. The AI provided guidance on task initiation, summarized reaction conditions from the relevant literature, suggested synthesis parameters, coded the BO model, generated the experimental conditions, and even managed the robotic platform and 3D printed the necessary equipment (Supporting Information, Figures S10 and S13). In terms of information exchange, our system relies on prompt engineering strategies and in-context learning (Supporting Information, Sections S2). When one assistant completes a task, its text-based output or findings serve as input for the next assistant. This allows for seamless collaboration and real-time adaptation, further enhancing efficiency and reducing the human workload. These efficiencies mean that a single researcher, even if newly initiated in the field, can achieve the productivity level of a team of research scientists.

Our primary objective is to identify the synthesis conditions under which the MOFs and COFs can achieve optimal crystallinity within a given number of experiment budgets. We hypothesized that the parameters to optimize for this purpose include the stoichiometry of the reactants, the modulator-to-linker ratio, the concentration levels, the duration of the reaction, and the temperature conditions. The complex nature of MOF and COF formation, however, presents a significant challenge due to the narrow window of optimal conditions.^51^ For example, in the quest to optimize the synthesis of MOF-321, given each variable ranging between 10 and 70 variations, the combinations would escalate to 6,101,172 synthesis conditions if a traditional high-throughput method were to be deployed to screen the entire parameter space of synthesis (Supporting Information, Section S4). While a human’s chemical intuition, often derived from previous work, can help reduce the number of experiments, it may also introduce unconscious biases favoring conditions they have used before, potentially overlooking unconventional conditions that could prove effective. Furthermore, human researchers generally struggle with screening multiple variables simultaneously due to the difficulty in quantifying their individual contributions.

**Figure 3:**
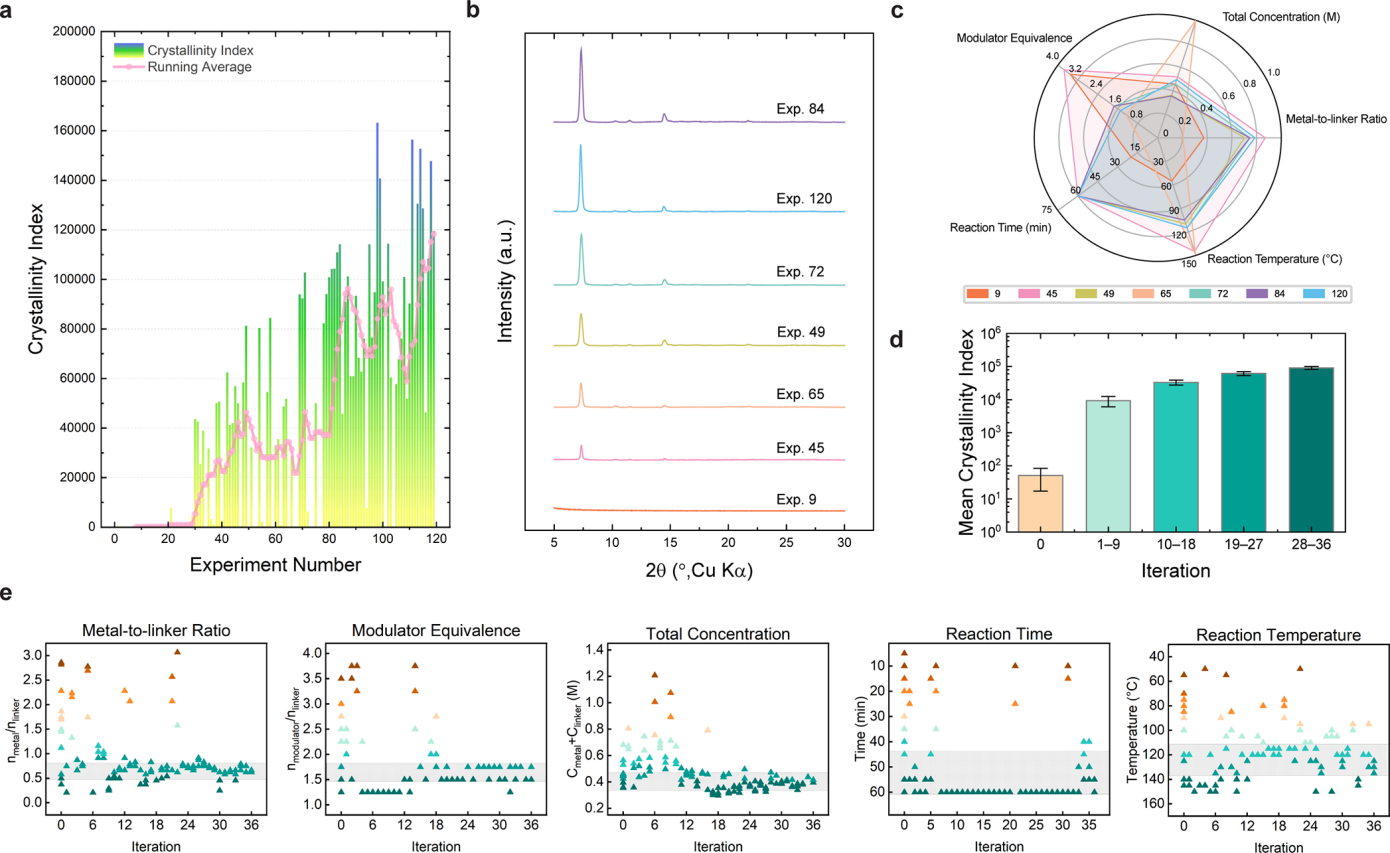
Outcomes of the AI-guided exploration for MOF-321 synthesis. (a) Plot displaying the crystallinity achieved per experiment across a total of 120 reactions, summing to 6,235 min, which is approximately 4.5 days with each experiment lasting 52 min on average. The initial 12 experiments utilized randomly selected conditions, while the subsequent 108 experiments were conducted across 36 iterations, with each iteration comprising 3 experiments. The running average of the crystallinity index, calculated over windows of 3 iterations (9 experiments), is displayed as a pink line. (b) PXRD patterns obtained from representative experimental samples and (c) detailed synthesis parameter distribution for these selected experiments displayed via a radar plot, revealing that the Bayesian search initially covers a broad variety space, later narrowing for fine-tuning. (d) Bar plot illustrating the mean and standard deviation of the crystallinity index for initial experiments (iteration 0) and subsequent iterations grouped into quartiles (iterations 1–9, 10–18, 19–27, and 28–36). The experiments suggested by the BO process significantly improve the average crystallinity compared to the initial 12 random experiments, and an increase in iteration numbers leads to better performance in later iterations. (e) Five scatter plots displaying the evolution of each synthesis parameter suggested by the BO algorithm as a function of iteration number.

In contrast, our approach employs Bayesian optimization, which suggests a set of three experimental conditions at a time by varying all five parameters simultaneously (Supporting Information, Section S9) and allowed us to effectively optimize the synthesis condition of MOF-321 within 120 experiments (Figure 3a and Table S1), thereby saving time and labor for running the rest of the 99.998% of the total ∼6 million potential combinations. To guide the iterative ML algorithm to search for the optimal condition, we define the objective variable, the crystallinity index (CI), as the height of the primary peak over its full width at half-maximum (FWHM). A sharper, narrower peak corresponds to a higher crystallinity index (Supporting Information, Figure S29). As shown in Figure 3b and c, through this process, our machine learning algorithm was able to evolve from a position of limited knowledge about the synthesis to determining the most suitable conditions for producing high-crystallinity MOFs. The ML model was initiated with 12 experiments (iteration 0) featuring randomly chosen synthesis parameters within the search space (Supporting Information, Section S4), providing a starting data set that displayed relatively low average CI values (Figure 3d).

Predominantly, these initial experiments resulted in MOFs with very poor or no crystallinity (Supporting Information, Figure S30). This is not surprising due to the vast size of the search space and the random nature of selecting initial conditions, resulting in low probabilities for identifying ideal synthetic conditions. This situation mirrors the challenges faced by researchers when initiating the synthesis parameter search for MOFs, as data interpretation can be challenging and choosing the subsequent experiment direction often proves difficult.

Nevertheless, as the BO model accrued more data points from subsequent iterations, the average CI values exhibited a consistent upward trend from iteration 1 to iteration 36. This improvement can be attributed to the nature of the ML-driven approach, which is not restricted to a specific combination of the synthesis parameters. Unlike human-driven attempts that usually focus on fine-tuning existing conditions, the ML model aims to explore a broad variety of synthesis conditions within as few experimental iterations as possible, maintaining a balance for the fine-tuning of specific parameters (Supporting Information, Section S9). This combination of exploration and exploitation within the synthesis condition domain progressively improved the average CI throughout the process and led to the identification of multiple optimal conditions, demonstrating the advantages of ML-driven optimization.

In MOF synthesis, subtle alterations in linker structure often necessitate drastically different optimal synthesis conditions.^52−55^ Overcoming human biases in experimental condition selection is a significant challenge in new crystalline materials discovery, and our AI-guided approach provides an opportunity to tackle this hurdle.^56^ Encouraged by the success of MOF-321 optimization, we extended our approach to a completely new MOF, using the organic linker H_2_TVDC instead of H_2_PZVDC and a different PXRD instrument. Success in this case would suggest that the process is (i) effectively generalizable to other MOFs and (ii) the approach is reproducible with PXRD instrument variations. As a result, we successfully obtained the optimal synthesis conditions for this new MOF-322 within 36 iterations, representing a total of 120 experiments (Supporting Information, Figures S40–S49 and Table S2). Note that the optimization process for MOF-322 began with a distinct set of 12 initial random experiments within the same search space. Moreover, the synthesis parameters under investigation were intentionally kept consistent with those used for MOF-321. This was done to illustrate that our method can be reliably applied to the different MOF without being overly sensitive to the initial conditions selected.

**Figure 4:**
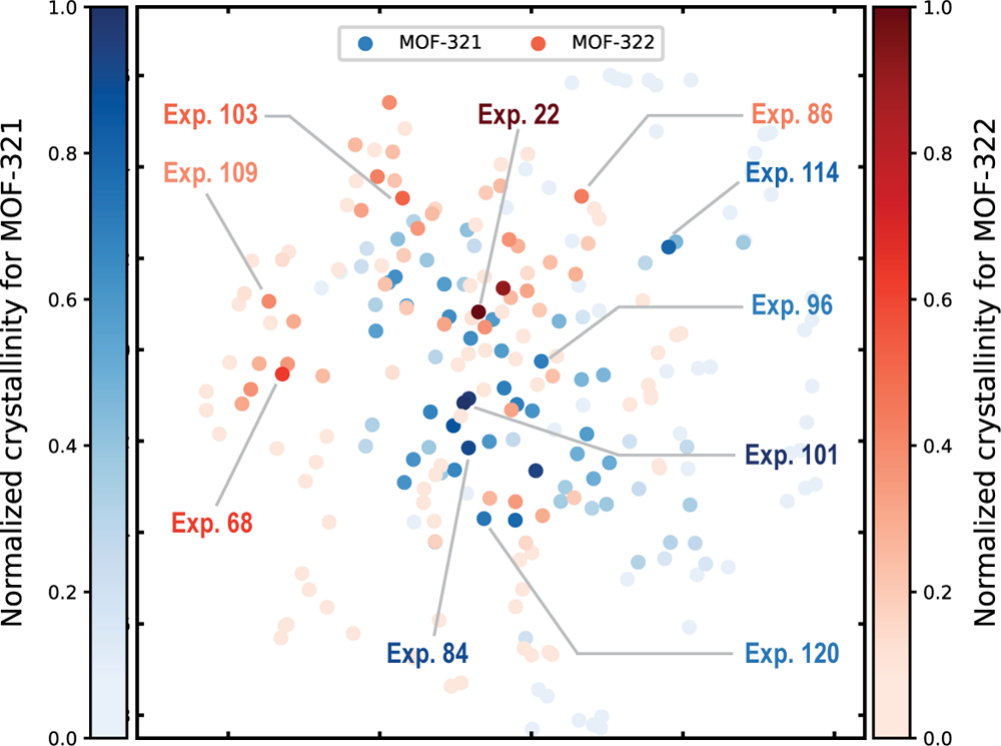
Two-dimensional t-SNE dimension reduction scatter plot representing 120 distinct synthesis conditions for MOF-321 (blue) and MOF-322 (red). Prior to reduction, the synthesis parameters (amount of metal, amount of modulator, solvent volume, reaction time, and temperature) are normalized. The color intensity indicates the crystallinity index, with deeper shades signifying higher values. Labels are provided for five representative synthesis conditions from various regions of the scatter plot, illustrating the distinctiveness of certain conditions and the successful identification of multiple conditions with high crystallinity by the BO process. The plot distinctly indicates that the optimal conditions for MOF-321 and MOF-322 differ.

Importantly, we discovered that this was due to the differences in the organic linker’s chemical and physical properties and the differently *cis*-connected aluminum SBUs, as indicated by the PXRD refinement (Supporting Information, Figure S50 and Table S4). MOF-322 has markedly different optimal synthesis conditions compared to MOF-321, as expected (Tables 1 and 2, Figure 4). For instance, while MOF-321 prefers the more traditionally used 120 °C synthesis condition with a metal-to-linker ratio ranging from 1:2 to 2:3 and 1.5 to 1.75 equivalence of the base modulator,^46,57^ MOF-322 requires a different set of conditions. Notably, while the experiment and ML algorithm for these compounds were independently executed, occasionally a condition yielding a highly crystalline MOF-321 sample was suggested for MOF-322, which sometimes, surprisingly, resulted in MOF-322 with low crystallinity or a side phase. Conversely, when a condition deemed favorable for MOF-322 is applied to MOF-321, surprisingly, the resulting compound may exhibit low crystallinity (Supporting Information, Tables S1 and S2). This suggests that the optimal conditions and screening windows for these two compounds greatly differ, and copying the best condition from one to the other is not an effective technique.

**Table 1:** Representative Conditions for the Microwave-Assisted Synthesis of High-Crystallinity MOF-321

Exp.	H_2_PZVDC (mmol)	Al^3+^ (mmol)	OH^–^ (mmol)	H_2_O (mL)	Time (min)	Temp. (°C)
84	1.0	0.75	1.75	4.7	60	125
96	1.0	0.70	1.5	4.0	60	105
101	1.0	0.46	1.75	3.6	60	120
114	1.0	0.66	1.75	4.3	45	120
120	1.0	0.66	1.5	4.0	55	135

**Table 2:** Representative Conditions for the Microwave-Assisted Synthesis of High-Crystallinity MOF-322

Exp.	H_2_TVDC (mmol)	Al^3+^ (mmol)	OH^–^ (mmol)	H_2_O (mL)	Time (min)	Temp. (°C)
22	1.0	0.46	2.0	3.6	40	145
68	1.0	0.21	1.75	1.5	35	145
86	1.0	0.41	1.5	4.3	40	150
103	1.0	0.46	2.0	3.4	60	140
109	1.0	0.99	2.0	3.5	50	150

Each new MOF to be optimized requires courage to explore new conditions; one cannot always rely solely on chemical intuition or stay within the comfort zone. As illustrated in the t-distributed stochastic neighbor embedding (t-SNE) dimension reduction scatter plot (Figure 4), the top five best conditions for MOF-321 and MOF-322 are markedly different, indicating their distinct synthesis conditions and different positions within the search space. This also indicates the validity and reproducibility of our approach in screening for good crystallinity conditions when a different MOF is selected.

To further demonstrate the efficiency of this ML-driven method in optimizing crystallinity, which is not only applicable to MOFs but also has broader applications, we applied this approach to COF-323 (Supporting Information, Table S3 and Figures S50–S58). This COF was considered to be a strong candidate for water harvesting due to its large pore volume and β-ketonenamine linkages.^58,59^ However, the significant chemical reactivity of 1,3,5-triformylphloroglucinol enables robust interactions with amine linkers, leading to the swift formation of amorphous solids.^60^ Consequently, the reported surface areas of this COF have been considerably below the theoretical value, accounting for merely 23–47% of the maximum theoretical value of 1550 m^2^/g.^61−63^ To surmount this challenge and circumvent laborious screening, we demonstrated that the BO process, mirroring its success in MOFs, efficiently identified several optimal conditions within 24 iterations, yielding highly crystalline COF-323 (Supporting Information, Figure S107). Importantly, throughout the ML-based closed-loop synthesis condition screening, the proposed screened conditions included not only those aligned with the traditional human approach but also those completely distinct from the conventional synthesis conditions for this type of COF (Supporting Information, Table S3 and Figure S60). These findings substantiate our hypothesis that ML can be used to transcend human biases about chemical behaviors.

As we progressed, having obtained several sets of conditions that yield high-crystallinity MOF-321, MOF-322, and COF-323, we became interested in conditions leading to optimal MOFs and COFs with maximized pore volumes for atmospheric water harvesting.^42,64^ We first evaluated the gas sorption behaviors of MOFs to show how the evolution of optimal synthesis conditions leads to enhanced porosity and water uptake. As demonstrated in Figure 5, we selected six different synthesis conditions of varying crystallinity index values for each MOF. Generally, samples with better crystallinity have a higher likelihood of exhibiting larger BET surface areas and pore volumes (Supporting Information, Figures S63–S74), resulting in greater water capacity (Supporting Information, Figures S86–S97).

**Figure 5:**
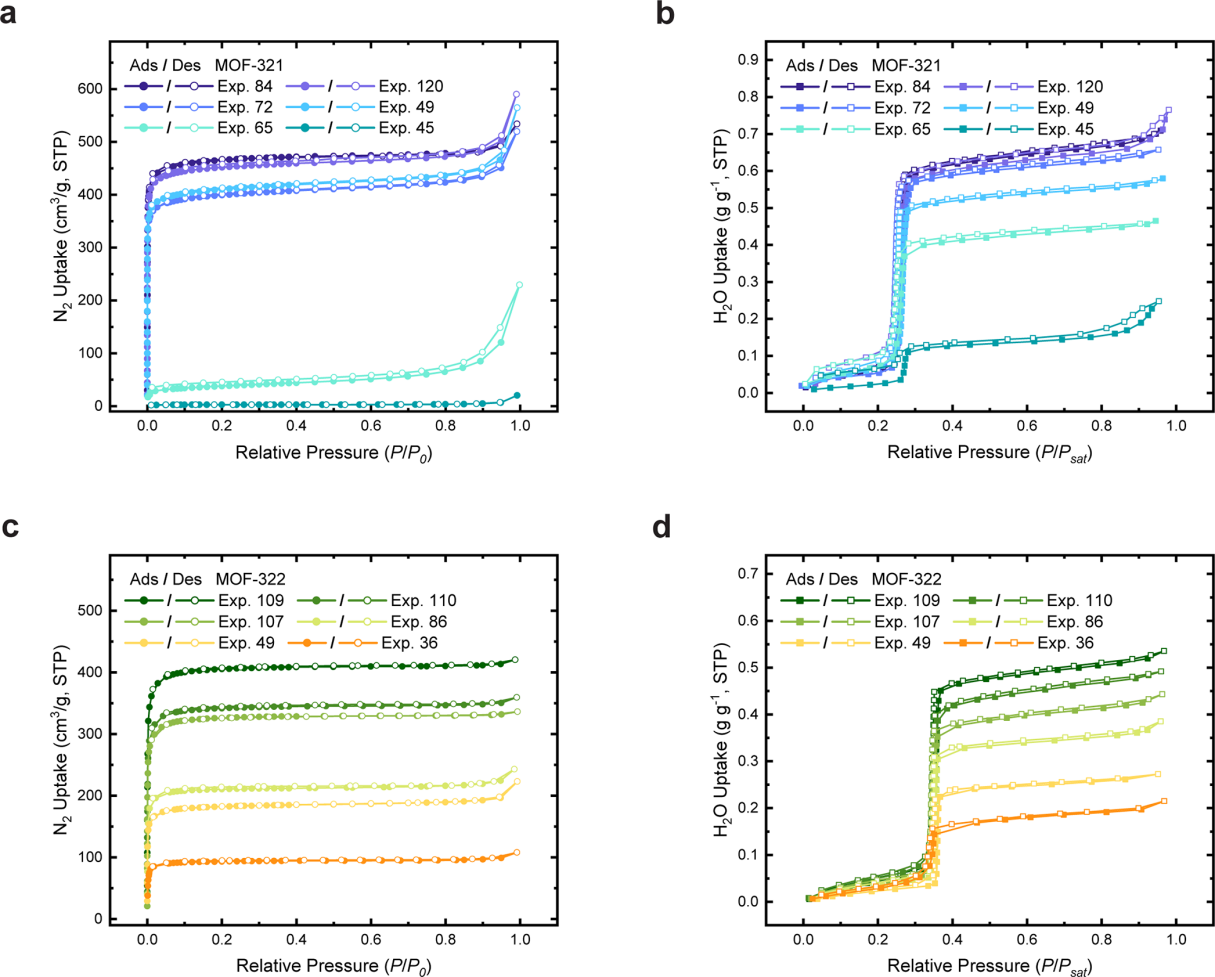
Overlay of gas adsorption–desorption isotherms of MOF-321 and MOF-322, prepared under varying synthesis conditions with different CI values, showing the evolution of optimal synthesis conditions within the search space. (a) Nitrogen sorption isotherms for MOF-321 samples obtained at 77 K. (b) Water vapor sorption isotherms for MOF-321 samples measured at 298 K, demonstrating different sorption capacities. (c) Nitrogen sorption isotherms for MOF-322 samples obtained at 77 K. (d) Water vapor sorption isotherms for MOF-322 samples measured at 298 K, showcasing different sorption capacities. Each panel presents data for six distinct samples of each MOF, underscoring the impact of synthesis conditions on the crystallinity and consequent gas adsorption properties of these MOFs. *P*, nitrogen or water vapor pressure; *P*_0_, 1 atm; and *P*_*sat*_, saturation water vapor pressure. Symbols of filled circles denote the adsorption branch, while empty circles denote the desorption branch.

However, it is important to note that while our aim for the BO algorithm is to find high-crystallinity compound synthesis conditions, higher CI values do not necessarily indicate high porosity and water capacity. This is because the CI is associated with the shape of the primary peak, while factors such as the presence of a side phase, unreacted starting materials, or linkers trapped in the pores could decrease the measured porosity and water uptake. This challenge exists for both human-dominated synthesis condition screening and ML-driven synthesis optimization. Nevertheless, while high CI values do not guarantee high water uptake, compounds with high water uptake invariably have high CI values. In our case, the BO process was remarkably effective, successfully identifying more than 10 combinations of conditions that yield MOF-321 and MOF-322 with sharp, narrow peaks (Supporting Information, Section S5). Upon verifying the porosity and water uptake of these promising candidates, we were able to find the most optimal conditions of 120 experiments to obtain the best sorption performance for each compound.

For MOF-321, the optimized Brunauer–Emmett–Teller (BET) surface area was determined to be 1875 m^2^/g, with an experimentally determined pore volume of 0.67 cm^3^/g. These measurements are close to the calculated theoretical values^65^ of 2025 m^2^/g for BET surface area and 0.72 cm^3^/g for pore volume. Moreover, a notable water uptake capacity of 0.66 g/g was observed, reflecting that exceptional porosity and desirable water sorption behavior of this compound were achieved. Similarly, the optimized MOF-322 demonstrated a BET surface area of 1584 m^2^/g, which accounts for 94% of the theoretical maximum BET surface area of 1686 m^2^/g. Additionally, the experimental measurement of the pore volume registered at 0.57 cm^3^/g, nearly paralleling the calculated volume of 0.61 cm^3^/g. This particular MOF demonstrated a water uptake of 0.53 g/g, further corroborating its efficient capacity. Collectively, these outcomes underscore the effectiveness of the human–AI collaboration in our system. It not only fosters discovery under synthesis conditions for high crystallinity, porosity, and water capacity but also drives these metrics toward an almost ideal benchmark, thus realizing our desired objectives with high productivity and reduced human labor

Concerning COF-323, our approaches helped identify five conditions with high BET surface areas ranging from 926 to 1459 m^2^/g among 82 conditions screened (Supporting Information, Figure S85). These conditions represent a diverse combination of synthesis parameters and demonstrate nearly twice the highest reported BET surface area of this COF in the literature,^63^ reaching 94% of the theoretical surface area (Supporting Information, Figures S83 and S84). The working capacity of the COF-323, synthesized under conditions recommended by BO, with respect to the 10 to 40% relative humidity (RH) range, reaches 440 cm^3^/g (0.35 g/g). This surpasses the performance of the human-synthesized COF (Supporting Information, Figure S98) and is comparable to that of other high-performing COFs such as AB-COF,^66^ COF-480-hydrazide,^67^ and others.^68−71^

## Concluding Remarks

We have developed a user-friendly AI-guided system that efficiently optimizes MOF and COF synthesis and requires no prior knowledge of coding. Our seven LLM-based assistants can facilitate various aspects of chemistry research, including planning, literature searching, ML model code writing, robotic operation, labware design and 3D printing, synthesis guidance, and experiment data extraction and analysis. While the Bayesian optimization algorithm, programmed by one of the assistants, plays a pivotal role in guiding researchers through the synthesis condition space, the contributions of the other LLM-based assistants are by no means negligible. They facilitate a wide array of wet laboratory activities, underscoring their broad adaptability. Together, these advancements led to the successful optimization of the green synthesis of MOF-321 and MOF-322 and the synthesis of COF-323, respectively, using microwave synthesis. Starting with no prior knowledge of the synthesis conditions, the ML model was able to precisely locate the narrow optimal synthesis window for these compounds to optimize crystallinity. This integrated system overcomes significant challenges, such as the difficulty of simultaneous parameter manipulation and human bias under synthesis conditions.

The increased number of successful trials led to the accelerated identification of optimal porosity and water adsorption capacity. Under microwave conditions, it took approximately 4 days (6,235 min) for 120 reactions to optimize the synthesis condition of 1 compound among over 6 million combinations of synthesis variables. Leveraging natural language to instruct LLM-based assistants and set up ML models, the integrated AI system in our laboratory took less than a month to build. Although the system is not yet fully automated, it can be significantly improved with more advanced robotic platforms. The recent development of function calling provides the potential for further upgrades, minimizing human interference and establishing a more automated system for synthesis optimization. This serves as a proof of concept to show the future blueprint of a chemistry laboratory: a team of AI will serve as assistants in different aspects and work together to greatly accelerate the discovery and optimization of new compounds in chemistry research; with minimal manual labor required, researchers can concentrate on innovative aspects.

## Supporting Information Available

Design and experimental details for microwave-assisted synthesis of MOFs and COF based on the Bayesian optimization method; additional descriptions of the prompt for ChatGPT assistants and information on the BO algorithm; synthetic procedures of organic linkers and NMR spectroscopy; and characterization details of the MOF and COF compounds, including elemental analysis, PXRD patterns, TGA curves, nitrogen sorption isotherms, and water sorption isotherms (PDF)
