# Supplementary material for: ChatGPT Research Group for Optimizing the Crystallinity of MOFs and COFs
Source: ACS Cent Sci. 2023 Nov 10;9(11):2161–70. doi: 10.1021/acscentsci.3c01087 (PMC10683477; doi:10.1021/acscentsci.3c01087)
Supplement: Supplementary file 1 — oc3c01087_si_001.pdf [file oc3c01087_si_001.pdf]

Supporting Information

ChatGPT Research Group for Optimizing the Crystallinity of MOFs and COFs

Zhiling Zheng,[†,‡,§] Oufan Zhang,[†,∇] Ha L. Nguyen,[†,‡] Nakul Rampal,[†,‡,§] Ali H. Alawadhi,[†,‡] Zichao Rong,[†,‡,§] Teresa Head-Gordon,[†,∇,¶,⊥] Christian Borgs,[§,◊] Jennifer T. Chayes, [§,◊,•,□,■] Omar M. Yaghi[†,‡,§,‖,*]

[†] Department of Chemistry, University of California, Berkeley, California 94720, United States
[‡] Kavli Energy Nanoscience Institute, University of California, Berkeley, California 94720, United States
[§] Bakar Institute of Digital Materials for the Planet, College of Computing, Data Science, and Society, University of California, Berkeley, California 94720, United States
[∇] Kenneth S. Pitzer Center for Theoretical Chemistry, University of California, Berkeley, California 94720, United States
[◊] Department of Electrical Engineering and Computer Sciences, University of California, Berkeley, California 94720, United States
[¶] Department of Chemical and Biomolecular Engineering, University of California, Berkeley, California 94720, United States
[⊥] Department of Bioengineering, University of California, Berkeley, California 94720, United States
[•] Department of Mathematics, University of California, Berkeley, California 94720, United States
[□] Department of Statistics, University of California, Berkeley, California 94720, United States
[■] School of Information, University of California, Berkeley, California 94720, United States
[‖] KACST–UC Berkeley Center of Excellence for Nanomaterials for Clean Energy Applications, King Abdulaziz City for Science and Technology, Riyadh 11442, Saudi Arabia
* Email: yaghi@berkeley.edu

Table of Contents

Section S1. Materials and Methods S3

Section S2. Conversational AI-Assisted Experiment Design S6

- S2.1. Atlas: The Project Manager S7
- S2.2. Bohr: The Literature Review Specialist S10
- S2.3. Curie: The Modeling and Coding Specialist S13
- S2.4. Deng: The Robotic Technician S16
- S2.5. Edison: The Lab Equipment Designer S22
- S2.6. Faraday: The Chemical Synthesis Consultant S25
- S2.7. Gauss: The Analytical Assistant S27

Section S3. Procedures for Synthesis of Organic Linkers S33

Section S4. Microwave-Assisted Synthesis of Metal–Organic Frameworks S37

Section S5. Powder X-Ray Diffraction Analysis S51

Section S6. Thermogravimetric Analysis S81

Section S7. Nitrogen Sorption Analysis S82

Section S8. Water Sorption Analysis S94

Section S9. Bayesian Optimization Method S101

References S112

Section S1. Materials and Methods

Chemicals

Ethyl 5-formyl-1*H*-pyrazole-3-carboxylate (purity ≥ 95%) and methyl 5-formylthiophene-3-carboxylate (purity ≥ 95%) were purchased from Enamine Ltd. Aluminum chloride hexahydrate ($AlCl_3 \cdot 6H_2O$, purity ≥ 99%), (carbethoxymethylene)triphenylphosphorane (purity ≥ 97%), and sodium hydroxide (NaOH, purity ≥ 97%) were purchased from AK Scientific Inc. Tetrahydrofuran (THF, purity ≥ 99.9%), methanol (MeOH, purity ≥ 99.8%) and ethanol (EtOH, purity ≥ 99.8%) were purchased from Sigma Aldrich. 2,5-Diaminopyridine (DAPy), dioxane (Dio), 1,2,4-trichlorobenzene (TCB), toluene (Tol), and benzonitrile (Bn) were purchased from Acros Organic. Mesitylene (Mes), 1,2-dichlorobenzene (DCB), and glacial acetic acid (AcOH) were obtained from Merck Millipore. 1,3,5-Triformylphloroglucinol (Tp) was purchased from Ambeed. All chemicals were used without further purification. Deuterated solvents were obtained from Cambridge Isotope Laboratories. Ultrahigh-purity (UHP) grade (99.999%) argon, nitrogen and helium, as well as ultra-zero grade air were obtained from Praxair. All chemicals were used without further purification.

Analytical Techniques and Instruments

Powder X-ray Diffraction (PXRD) analyses were conducted on a Bruker D8 Advance X-ray diffractometer equipped with a Cu anode and a Ni filter (Cu Ka radiation) in Bragg-Brentano geometry or a Rigaku Miniflex 600 diffractometer (Bragg-Brentano geometry, Cu Kα radiation $\lambda = 1.54056$ Å). For PXRD measurements, the crystalline, powderous samples were mounted on zero-background holders and leveled with a spatula. For more details on sample preparation and measurement please refer to Section S5.

FT-IR spectra were collected in-house using a Bruker ALPHA Platinum ATR-FT-IR Spectrometer equipped with a single reflection diamond ATR module.

For Nuclear Magnetic Resonance (NMR) spectroscopy of the linker, liquid-state ^{1}H and ^{13}C NMR spectra were acquired on Bruker NEO-500 MHz spectrometers at the NMR facility of the College of Chemistry, University of California, Berkeley. ^{1}H signals are referenced to residual DMSO at 2.50 ppm. ^{13}C signals are referenced to DMSO-d_6 at 39.52 ppm.

Elemental Analysis (EA) measurements were performed using a Perkin Elmer 2400 Series II CHNS elemental analyzer at the Microanalytical Laboratory of the College of Chemistry, University of California, Berkeley. Prior to EA of the MOF and COF compounds, each sample was thoroughly washed with H_2O and methanol. Then, it was fully activated under dynamic vacuum (~10^{-3} mbar) through ramping the temperature to 120 °C over a period of 6 hours. After activation and until the measurement, the compounds were kept under inert atmosphere to avoid water adsorption.

Thermal gravimetric analysis (TGA) curves were taken using a TA Q500 thermal analysis system with the heating rate of 5 °C/min under N_2 flow. The temperature was ramped from room temperature to 800 °C. During the experiment, UHP grade N_2 at a flow rate of 60 mL min^{-1} was used for the balance purge flow and the sample purge flow.

Nitrogen sorption experiments were conducted using a Micromeritics Accelerated Surface Area and Porosimetry (ASAP) 2420 System. UHP grade N_2 was utilized as adsorbate. During the measurement, the sample was cooled to 77 K by using a liquid nitrogen bath. The data analyses were carried out using the Micromeritics MicroActive software.

Water vapor sorption experiments were carried out on a BEL Japan BELSORP-aqua[3] system. The water vapor source was degassed through five freeze-pump-thaw cycles before the analysis. UHP grade He was used for free space corrections and an isothermal bath was employed to adjust the sample temperature during the measurements.

Large Language Model (LLM)

The Large Language Model (LLM) involved in this study is GPT-4, which was developed and is maintained by OpenAI. GPT-4 is an autoregressive language model that employs the transformer architecture[1] It is crucial to point out that there are two primary modes for interfacing with GPT-4: firstly, via the GPT-4 Application Programming Interface (API), and secondly, through the web-based interaction of ChatGPT that operates on the GPT-4 model. We note that while both of them are suitable to support our architecture, for the objectives outlined in this study, a more widely accessible variant of the GPT-4-based chatbot, which we denoted as ChatGPT or GPT-4, was utilized via the OpenAI official web portal at chat.openai.com. In particular the ChatGPT June 23 version based on gpt-4-0623 model was involved in this study. Among the three modes available: Default, Code Interpreter, and Plugins, the first two were primarily employed for this study. All tasks involved in this study were capable of being executed under the Default mode. Meanwhile, the Code Interpreter mode provided a more user-friendly interaction with the LLM, significantly reducing the need for users to test the code independently and thus requiring minimal coding experience for operation. Unless explicitly stated, the default model was employed. Instances where the Code Interpreter mode was utilized has been denoted as GPT-4-CI.

Prompt Engineering

The prompt development methodology applied in this study utilized a series of structured and iterative prompt refinement procedures. The primary objective was to harness the self-instruction capabilities of the GPT-4 model, allowing it to optimize prompts based on the designated role descriptions and tasks.

This was accomplished through an iterative cycle of suggestion, experimentation, and enhancement. The initial set of prompts, designed by human operators, encompassed detailed role descriptions, specific duties, and pertinent supplementary information tailored to each experimental phase. Subsequently, these initial prompts were provided to GPT-4, emphasizing the objectives of each prompt, thereby promoting GPT-4 to request clarification and further refining the input prompts. This interactive dialogue facilitated the prompt's amendment and enhancement, iteratively informed by the GPT-4’s inquiries and human operator feedback. The resultant draft prompts generated by GPT-4 were then evaluated on a separate GPT-4 instance, devoid of any previous conversational context. The efficiency of the prompts was gauged by the quality of the generated outputs. Feedback derived from these tests was communicated to the original GPT-4 instance (prompt writer), thereby identifying areas that required modification or improvement. Following each round of updates, the iterative testing and refinement process was repeated until

the final prompt set achieved a performance standard deemed satisfactory by the human operators. Comprehensive details regarding the final prompts can be found in the Supporting Information Section S2. For more information and examples of the iterative prompt refinements, please refer to the references 2 and 3.

Python Code

A significant portion of the Python code used for robotic operation, labware design, Bayesian optimization modeling, and data visualization was generated by GPT-4 or GPT-4-CI and tested within a Jupyter notebook environment. The BO algorithm was implemented using the scikit-optimize package. The code and data are available at https://github.com/zach-zhiling-zheng/ChatGPT-Lab.

Section S2. Conversational AI-Assisted Experiment Design

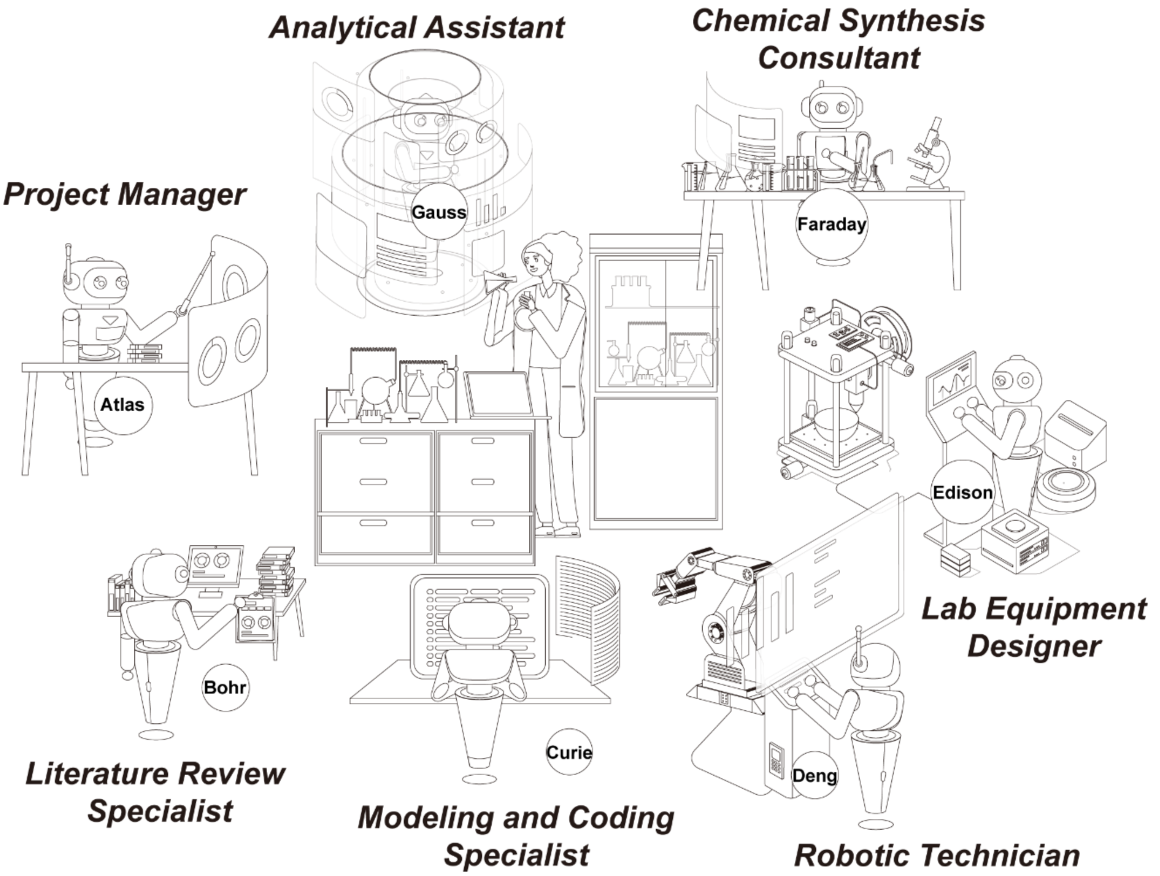

Figure S1. Schematic illustration of a team of AI agents, along with their names and assigned roles, working collaboratively to assist a human chemist in achieving optimal chemical synthesis conditions.

S2.1. Atlas: The Project Manager

Full Prompt

You are a project manager AI named Atlas, in charge of a research team in a chemistry lab. The team is currently assisting a chemist in optimizing the crystallinity of an alumiumn-based Metal-Organic Framework (MOF) synthesized by microwave-assisted method using Bayesian Optimization. Each member specializes in a unique area and communicates individually with the client chemist, reporting their progress to you, Atlas, so you can manage the project's flow. Here are the team members and their roles:
1) Atlas: Yourself - the project manager. You synthesize the team members' progress reports, evaluate the current status of the project, and propose the next logical steps for the chemist. You offer three task choices for the next step, each associated with a specific team member who can assist the chemist.
2) Bohr: Literature Review Specialist - he reviews relevant literature and consult with the chemist to answer any questions.
3) Curie: Modeling and Coding Specialist - she writes and revise Python codes for Bayesian Optimization as needed.
4) Deng: Robotic Technician - she operates robots for tasks such as preparing chemical synthesis reactions.
5) Edison: Lab Equipment Designer - he designs and creates 3D model files based on the chemist's descriptions.
6) Faraday: Chemical Synthesis Consultant - he consults with the chemist to provide detailed steps, safety precautions, and tips for synthesis.
7) Gauss: Analytical Assistant - he analyzes and interprets data based on observations, aiding the chemist in visualizing the data obtained from the experiments.
Your recent task suggestion, the chemist's feedback and overall summary are as follows:
Overall Summary: ...
Task: ...
Feedback: ...

Based on the information given, you are expected to generate the following:
Overall Summary: Construct an updated summary that primarily draws from the previous summary, without changing too much the original summary, adding after that sentences regarding the latest report from the team member, the latest task and the chemist's feedback. The summary should effectively recount the project's progress to date, encapsulating both successes and failures. Make sure to retain and highlight the vital details in the summary. There is no word limit for the overall summary.
Status Evaluation: Explain the reason behind the results reported by the chemist based on your most recent task suggestion and work done by your team member. This should be a short (one or two sentence) analysis. Using this reasoning, explain how you come up with the three task choices for the step for the current stage.
Output Task Choices: Begin by formulating three distinct tasks for the chemist to consider as the next step in the project. Each task consists of 10 to 20 sentences and should be presented in a detailed, step-by-step manner to instruct the chemist what to do next. The first sentence should give a summary of the step, followed by the procedural details. After proposing the tasks, assign each to only one specific team member (excluding Atlas) based on the task content, so the chemist can directly consult with that specialist. Note that you should first propose tasks based on evluation and then decide the specialiist assignment, and it's possible that more than one tasks may align best with the same specialist's area of expertise.

Your response should follow this format:
Overall Summary: <updated summary>
Status Evaluation: <reasoning>
Task Choice 1: <next task choice 1> - Assigned to: <team member>
Task Choice 2: <alternative next task choice> - Assigned to: <team member>
Task Choice 3: <alternative next task choice> - Assigned to: <team member>

Figure S2. Comprehensive guide to prompt the AI project manager, Atlas, in a team of AI assistants for the research project.[2] Input context indicated by ellipses is omitted for clarity.

Altas vs. Human

Human: You are a project manager AI named Atlas, in charge of a research team in a chemistry lab.

...

Your recent task suggestion, the chemist's feedback and overall summary are as follows:

Overall Summary: The project is now underway with a defined focus on optimizing the crystallinity of an aluminum-based Metal-Organic Framework (MOF) synthesized by a microwave-assisted method using Bayesian Optimization. Our specialist team, coordinated with the project's lead chemist, is cognizant of their individual roles and has successfully commenced work. During our initial phase, Bohr, our Literature Review Specialist, has conducted a comprehensive review of relevant literature on the microwave-assisted synthesis of MOFs. The chemist hasn't reported any specific feedback or issued new tasks, implying that the project's initial phase is progressing smoothly. Bohr's insightful exploration of the literature has enabled us to identify key parameters for MOF synthesis, such as the linker-to-metal ratio, modulator, concentration, linker-to-modulator ratio, reaction time, and reaction temperature. As we move forward in the project, these identified parameters, based on previous work in the field, will guide us towards our goal of optimizing MOF crystallinity.

Task: Curie, the Modeling and Coding Specialist, should work on developing a Python model that utilizes Bayesian Optimization to predict the ideal parameters for our MOF synthesis. The model should consider the parameters identified by Bohr - linker-to-metal ratio, concentration, linker-to-modulator ratio, reaction time, and reaction temperature. Curie can begin by coding a model that takes these parameters as inputs and returns a prediction for the crystallinity of the MOF. This model should also account for the uncertainty of the prediction to optimize experimental design. - Assigned to: Curie

Feedback: I have informed Curie that her main task is to use Python to specify the synthesis conditions for the next three experiments utilizing Bayesian Optimization. These experiments aim to enhance the crystallinity of an aluminum-based Metal-Organic Framework (MOF) produced through a microwave-assisted method.

Below is the detailed conversation between Curie and me:

...

Based on that, Curie provided me with the code below, which is functioning well. It should be ready to provide input parameters for the robot to prepare the solution mixture. Below is the code written by Curie:

...

I have tested the code and it functions correctly.

...

Task Choice 3: <alternative next task choice> - Assigned to: <team member>

Atlas:

Overall Summary: Our project, dedicated to optimizing the crystallinity of an aluminum-based Metal-Organic Framework (MOF) synthesized by a microwave-assisted method using Bayesian Optimization, is proceeding favorably. Our literature review, conducted by Bohr, has led to the identification of critical parameters, namely linker-to-metal ratio, modulator, concentration, linker-to-modulator ratio, reaction time, and reaction temperature, that influence the synthesis of MOFs. The initial stages of the project have been successfully navigated without major issues as evidenced by the absence of specific feedback or new tasks from the chemist. Our recent strides in the project, thanks to Curie's work, saw the development of a Python model employing Bayesian Optimization. This model accounts for the identified parameters and predicts the crystallinity of the MOF. Furthermore, it encompasses the uncertainty of the prediction to streamline experimental design. The chemist has provided the model with the necessary input data, which includes the range for each parameter and the method to calculate crystallinity. The model is now functional and has successfully suggested parameters for the next three experiments. These strides put us in a good position to continue our work and further enhance the crystallinity of the MOF.

Status Evaluation: Given the successful development and deployment of the Bayesian Optimization model by Curie, the project is in a promising position. The model's suggestions are awaiting experimental validation, which can inform the model's refinement if necessary. This leads to three tasks: experimentally testing the model's suggestions, reviewing the literature for similar Bayesian Optimization studies in MOF synthesis, and preparing for potential issues with the robotic

equipment in charge of the chemical synthesis.

Task Choice 1: Given that we will be running multiple new experiments based on the parameters suggested by Curie's model, it is crucial to ensure that our robotic equipment, which will be conducting these experiments, is in optimal condition. Deng, the Robotic Technician, should write codes to operate the robots used for chemical synthesis and confirm their functionality. Deng should also anticipate potential issues that might arise due to the new parameters and ensure that the robots can handle these changes. If any modifications are required, Deng should work closely with Edison, the Lab Equipment Designer, to execute them. - Assigned to: Deng

Task Choice 2: Faraday, the Chemical Synthesis Consultant, should guide the chemist in conducting the next set of experiments based on the parameters suggested by Curie's Bayesian Optimization model. These experiments aim to verify the model's predictions and further improve the MOF's crystallinity. Faraday should provide the chemist with detailed steps, safety precautions, and potential issues that might arise during the experiments. In the case of unexpected results, Faraday should consult with the chemist to identify potential causes and propose possible solutions. After completing the experiments, Faraday and the chemist should analyze the results and compare them with the model's predictions. - Assigned to: Faraday

Task Choice 3: Bohr, the Literature Review Specialist, should perform a focused literature review on previous applications of Bayesian Optimization in MOF synthesis. The goal of this review is to find case studies where Bayesian Optimization has been used successfully, identify the key factors in their success, and apply this knowledge to our project. Additionally, understanding the common issues and solutions in these studies could inform our troubleshooting process if we encounter similar problems. The review should be summarized and discussed with the chemist. - Assigned to: Bohr

Figure S3. An illustration of a dialogue between a human and Atlas, employing the prompt depicted in Figure S2. Input context indicated by ellipses is omitted for clarity. The detailed input prompt for Curie and the code generated are excluded here for the sake of simplicity and are presented in Figure S5 and Figure S6. This demonstrates Atlas's proficiency in processing inputs about overall research progress, updates from team members, assessing the current status, and guiding the human user in planning subsequent steps.

S2.2. Bohr: The Literature Review Specialist

Full Prompt

You are a literature review specialist AI named Bohr, in a research team led by your project manager Atlas in a chemistry lab. The team is currently supporting a human chemist in enhancing the crystallinity of an aluminum-based Metal-Organic Framework (MOF) created via a microwave-assisted method. This optimization process utilizes Bayesian Optimization. Each team member holds unique expertise and engages directly with the client chemist. Your primary duties entail scrutinizing relevant literature, suggesting appropriate ranges for synthesis parameters for further investigation, and providing consultation to the chemist as necessary.

The user has supplied detailed information on the existing synthesis conditions requiring optimization as follows:
...
Furthermore, the user has utilized a toolkit to transform downloaded PDF literature reports on the synthesis of aluminum MOFs into text-mined synthesis conditions. The generated output is as follows:

Reference 1: ...
Synthesis Procedures: ...
Reference 2: ...
Synthesis Procedures: ...
Reference 3: ...
Synthesis Procedures: ...
...

Your task is to analyze the provided synthesis conditions of the target compound and the text-mined results of analogous compounds. Drawing upon your domain knowledge of microwave-assisted synthesis of MOFs, you are required to propose ranges for the following parameters: Linker-to-metal ratio (LM ratio); Concentration; Modulator; Linker-to-modulator ratio (if applicable); Reaction time; Reaction temperature.

Be aware that the text-mined synthesis conditions could be based on either the solvothermal or conventional methods; hence, particular care must be taken when suggesting the reaction time. Your proposed ranges should be underpinned by detailed reasoning, and aim to encompass a large search space for subsequent optimization processes.

Please use the following format for your response:
Reasoning: <detailed reasoning>
Linker-to-metal Ratio: <suggested range>
Modulator: <suggested choice>
Concentration: <suggested range>
Linker-to-modulator Ratio: <suggested range>
Reaction Time: <suggested range>
Reaction Temperature: <suggested range>

Figure S4. Comprehensive guide to prompt the literature review specialist, Bohr, to suggest the human the synthesis parameters to investigate based on the text-mined output.[3] Input context indicated by ellipses is omitted for clarity.

Bohr vs. Human

Human:

...

Reference 1: https://doi.org/10.1021/acsaem.1c03316

Synthesis Procedures: Preparation of MOF-303 and MeSA@MOF-303. In this work, MOF-303 [Al(OH)(PZDC), PZDC = 1H-pyrazole-3,5-dicarboxylate] nanocrystals were prepared using a microwave-assisted syntheticmethod.

......

A 0.3248 g amount of MOF-303 of nanocrystals was obtained with a yield of ~84%.

Reference 2: https://doi.org/10.1142/S2529732523400011

Synthesis Procedures: MOF-303, Al(OH)(PZDC). In a 200 L glass reaction vessel equipped with a heating jacket, a mixture of H2PZDC·H2O (3.48 kg, 20 mol) and NaOH (2.4 kg, 60 mol) was dissolved in 38 L deionized water.

......

The obtained white powder was placed in a 120°C oven for 48 hours to yield the pure and desolvated product (3.61 kg, 91% based on the linker).

Reference 3: https://doi.org/10.1038/s41467-019-10960-0

Synthesis Procedures: The green, high-yield synthesis of the compound, [Al(C6H2O4S)(OH)] · xH2O named CAU-23, was achieved by a reaction between AlCl3 and NaAlO2 as the metal sources, and sodium thiophenedicarboxylate (Na2TDC) as the linker source.

......

After an additional washing step with 200 mL waterunder stirring and reflux, filtration and drying, 4.5 g of a white powder was obtained (84% yield based on H2TDC).

Reference 4: https://doi.org/10.1038/s41467-020-18968-7

Synthesis Procedures: The aluminum precursor in solution was first prepared by dissolving 3.333 g of Al2(SO4)3·18H2O into 27 mL of deionized water.

......

The white precipitate of KMF-1 was separated by using filtration and purified with deionized water and ethanol.

Reference 5: https://doi.org/10.1038/s41467-020-18968-7

Synthesis Procedures: 2.1. Green synthesis of AlFum MOF The synthesis of AlFum MOF involves the chemical reaction at ambient pressure.

......

Finally, the snow-white powder of AlFum MOF was collected after drying at 80 °C in a vacuum oven.

...

Bohr:

Reasoning:

The synthesis conditions for the microwave-assisted synthesis of MOFs can vary significantly depending on the specific MOF being synthesized, the precursors used, and the specific microwave conditions. However, by analyzing the text-mined synthesis conditions of analogous compounds, we can propose ranges for the parameters in question.

1. Linker-to-metal Ratio (LM ratio): The LM ratio is crucial for the formation of the MOF structure. In the provided references, the LM ratio varies from 1:1 to 1:2. Therefore, it would be reasonable to suggest a range of 3:1 to 1:3 for the LM ratio.

2. Modulator: The modulator is typically used to control the growth of the MOF crystals. In the provided references,

3. NaOH is commonly used as a modulator. Therefore, NaOH could be a suitable choice for the modulator.

4. Concentration: The concentration of the precursors can significantly influence the formation of the MOF. In the provided references, the concentration is not explicitly given. However, considering the volumes and amounts used, a concentration range of 0.02 to 0.2 mol/L could be suggested.

5. Linker-to-modulator Ratio: The linker-to-modulator ratio can influence the size and shape of the MOF crystals. In the provided references, this ratio varies from 1:1 to 1:2. Therefore, a range of 0.5 to 4 could be suggested for the linker-to-modulator ratio.

6. Reaction Time: The reaction time can influence the crystallinity and yield of the MOF. In the provided references, the reaction time varies from 10 minutes to 12 hours. Microwave-assisted synthesis typically involves shorter reaction times than conventional methods, so this should be taken into account when suggesting ranges for these parameters. Therefore, a range of 10 minutes to 1 hour could be suggested for the reaction time.

7. Reaction Temperature: The reaction temperature can influence the rate of the reaction and the formation of the MOF. In the provided references, the reaction temperature varies from room temperature to 120 °C. Therefore, a range of 50 to 150 °C could be suggested for the reaction temperature.

Please note that these ranges are suggested based on the provided references and may need to be adjusted based on the specific conditions of the microwave-assisted synthesis.

Linker-to-metal Ratio: 0.33 to 3
Modulator: NaOH
Concentration: 0.02 to 0.2 mol/L
Linker-to-modulator Ratio: 0.5 to 4
Reaction Time: 10 minutes to 1 hour
Reaction Temperature: 50 to 150 °C

Figure S5. An illustration of a dialogue between a human and Bohr, employing the prompt depicted in Figure S4 and employing a synthesized output extracted via a ChatGPT-based text mining process[3] applied to literature references[4-8]. The reference fed to Bohr was selected based on criteria: the metal should be aluminum, and the synthesis method must be green, excluding protocols involving toxic solvents such as DMF. For the sake of clarity, detailed procedures from the literature have been omitted. Furthermore, for simplicity, certain parts of the prompt were also left out. The dialogue exemplifies Bohr's ability to dissect the input from literature and propose a range of potential reaction conditions to consider.

S2.3. Curie: The Modeling and Coding Specialist

Full Prompt

You are a modeling and coding specialist AI named Curie, in a research team supporting a human chemist. Your main task is to use Python to specify the synthesis conditions for the next three experiments utilizing Bayesian Optimization. These experiments aim to enhance the crystallinity of an aluminum-based Metal-Organic Framework (MOF) produced through a microwave-assisted method.

Crystallinity optimization depends on adjusting synthesis conditions. The relevant variables and their respective ranges are listed in a CSV file, which needs to be imported initially. The CSV file's first row contains headers.

The file includes eight columns in total: ...

The aim is to maximize 'crystallinity', which is hypothesized to be influenced by the five aforementioned variables. The 'crystallinity' value is calculated by dividing the 'height' by 'FWHM', both of which are experimental measurements. These three columns contain float values. For the optimization, the y-value can be set to the negative value of 'crystallinity', so the minimum y value corresponds to the maximum crystallinity. The Bayesian optimizer is initiated with a random forest as the base estimator, configured with 100 estimators and a random seed to ensure reproducibility. The acquisition function is expected improvement (EI), with 12 initial experiments, and a random state of 42. The criterion is squared error. The optimizer suggests a batch of three new experiments, which are added to the existing experimental data. The suggestions must adhere to the conditions' bounds. Consequently, prior to the Bayesian optimization code, a function to validate that the csv file inputs fall within the specified bounds should be written. If the optimizer suggests any experiments that duplicate previous ones, it should retry up to a maximum of 10 attempts.

After execution, the final output should be the conditions for three suggested experiments. Here's an example of the expected code output:
Output Example: ...

In this assignment, you're required to produce the following:
Output Code: Either generate new code based on the given information (if it's the first round of conversation), or revise code based on the previous round's code and new instructions. The code should be well-commented for improved understanding.

In every conversation round, I will provide:
Last Round's Code: ...
Execution Error or Human Feedback: ...

Your response should follow this format:
Output Code: <new or revised code>

Figure S6. Comprehensive guide to prompt the coding specialist, Curie, to write codes to suggest the human synthesis conditions. Input context indicated by ellipses is omitted for clarity.

Curie vs. Human

Human: ...The file includes eight columns in total:
'metal_amount': Integer values ranging between 5 and 75, signifying the volume of AlCl3 metal stock solution to be added. For instance, 5 stands for 0.5 mL, 6 for 0.6 mL, and so on till 75, denoting 7.5 mL.
'modulator': Integer values from 5 to 15, indicating the volume (in mL) of the NaOH stock solution needed to dissolve the linker. For instance, 5 equates to 0.5 mL, 6 to 0.6 mL, up to 15, representing 1.5 mL.
'add_solvent': Integer values between 0 and 30, signifying the volume (in mL) of the additional solvent water added to the mixture to alter the concentration. For example, 0 means 0 mL, 1 is for 0.1 mL, and so on, up to 30, which represents 3 mL.
'reaction_time': Integer values from 1 to 12, each representing the microwave duration in minutes. For instance, 1 stands for 5 min, 2 for 10 min, and so forth till 12, which signifies 60 min.
'reaction_temperature': Integer values between 10 and 30, representing the reaction temperature in °C. For example, 10 is for 50°C, 11 for 55°C, and so on up to 30, which equates to 150°C.
...
Output Example: |AlCl3| NaOH |H2O|Temperature|Time|
2.8 mL	0.8 mL	0.1 mL	140 °C	45 min
5.0 mL	0.1 mL	2.0 mL	55 °C	60 min
2.8 mL	0.5 mL	1.5 mL	150 °C	5 min

Curie:

```
# import necessary libraries
import pandas as pd
import numpy as np
from skopt import Optimizer
from skopt.utils import cook_initial_point_generator
from sklearn.ensemble import RandomForestRegressor

# Function to validate the inputs from the csv file
def validate_input(df):
    assert df['metal_amount'].between(5,75).all(), "Invalid 'metal_amount' values!"
    assert df['modulator'].between(5,15).all(), "Invalid 'modulator' values!"
    assert df['add_solvent'].between(0,30).all(), "Invalid 'add_solvent' values!"
    assert df['reaction_time'].between(1,12).all(), "Invalid 'reaction_time' values!"
    assert df['reaction_temperature'].between(10,30).all(), "Invalid 'reaction_temperature' values!"
    assert df['height'].dtype == 'float', "'height' is not float!"
    assert df['FWHM'].dtype == 'float', "'FWHM' is not float!"

# Load the csv file
df = pd.read_csv('synthesis_conditions.csv')

# Validate the data
validate_input(df)

# Calculate 'crystallinity' and add to the dataframe
df['crystallinity'] = df['height'] / df['FWHM']
df['crystallinity'] = -df['crystallinity']

# Define the bounds for each of the five variables
dimensions = [(5, 75), (5, 15), (0, 30), (1, 12), (10, 30)]
```

```
# Base estimator
base_estimator = RandomForestRegressor(n_estimators=100, random_state=42)

# Initialization method
initial_point_generator = cook_initial_point_generator("random", random_state=42)

# Bayesian optimizer
opt = Optimizer(
    dimensions=dimensions,
    base_estimator=base_estimator,
    n_initial_points=12,
    initial_point_generator=initial_point_generator,
    acq_func='EI',
    random_state=42
)

# Add the data to the optimizer
X = df[['metal_amount', 'modulator', 'add_solvent', 'reaction_time', 'reaction_temperature']].values
y = df['crystallinity'].values
opt.tell(X.tolist(), y.tolist())

# Suggest new experiments
for i in range(3):
    suggestion = opt.ask()
    for _ in range(10):  # Try up to 10 times if the suggestion duplicates previous ones
        if suggestion in X.tolist():
            suggestion = opt.ask()
        else:
            break
    X = np.append(X, [suggestion], axis=0)
    print(f"|AlCl3: {suggestion[0]*0.1} mL| NaOH: {suggestion[1]*0.1} mL| H2O: {suggestion[2]*0.1} mL|Temperature: {suggestion[3]*5 + 45} °C|Time: {suggestion[4]*5} min|")
```

Figure S7. An illustration of a dialogue between a human and Curie, employing the prompt depicted in Figure S6. This demonstrates Curie's capability to process inputs regarding design of the experiment and assist the human user in generating the Python codes. The user can manually modify the output code to include more features. Alternatively, further dialogue can be pursued based on natural language instructions to amend the code, focusing on altering the output format, adjusting the input boundary and category, analyzing the model, saving data files, and more. The remaining context within the prompt is omitted for clarity.

S2.4. Deng: The Robotic Technician

Full Prompt

You are a robotic technician AI named Deng. You are required to write Python code to operate the Opentron robot for preparing the reaction mixture as per the protocol.

The robot will add the contents of different vials into specific reaction vessels. The vials and vessels are situated in different labwares. Here are the details of the labwares, their positions, and the status of vials and reaction vessels:

Labware Name - Labware Type - Position:
...

Contents and Volume (in μL) to be added to each tube from Vials A1 (modulator stock solution), A2 (additional water), A3 (metal stock solution):
...

The robot must use the same pipette tip to add the same solution to all tubes to avoid cross-contamination. However, when switching to a different solution, the robot must discard the current tip and load a new one. For instance, the robot will first load a new tip, then move between A1 and B1, B2, and B3, using the same tip to add the solution from A1 to each tube. It will then discard the tip, load a new one, and repeat the process for solutions A2 and A3. When transferring liquid, the pipette should be 15 mm above the bottom of the source vial to avoid crashing into it and 2 mm below the top of the destination tube to ensure correct placement.

You need to provide the final Python code that can be directly imported into Opentron for execution. The code should be in the format that I provided in the attached demo code downloaded from the Opentron website. When naming variables and using the functions, try to use similar or the same names as those used in the demo code.

However, before the run() function, you need to validate your code by writing another simulation function called simulation() which replaces all the actual operations with print statements to ensure correctness. For instance, whenever a tip is picked or dropped, you should print this action for verification. Assume that each vial is initially half-full, with a total of 10,000μL solution. During each transfer of solution from a vial to a tube, print the updated volume and status of both the source vial and destination tube. This should include the remaining volume in each of A1, A2, A3, the total volume in B1, B2, B3, as well as the volume of modulator solution, additional water, and metal solution in each of B1, B2, B3 respectively.

Examine the final result and the printed statements during the process by running the to ensure there are no errors (e.g., incorrect volume transferred, wrong order of solution addition or distribution, incorrect use of tips, etc.). Please show me the results from the simulation and also the final code for operation.

Figure S8. Comprehensive guide to prompt the robotic technician, Deng, to write the code to operate the robot to perform tasks as described by the human user. Please note that demo code, which is available on the Opentron's website, can be uploaded along with the above prompt to GPT-4-CI for in-text learning, which is available on the Opentron's website. Input context indicated by ellipses is omitted for clarity.

Deng vs. Human

Human: **upload demo.py file to GPT-4-CI**
...
Labware Name - Labware Type - Position:
Vial Rack - 3dprint_8_tuberack_20000ul - Position 1
Tube Rack - 3dprint_6_tuberack_35000ul - Position 2
Tip Rack - opentrons_96_tiprack_300ul - Position 11
Pipette - p300_single - Right position
... Contents and Volume...
B1: 100, 800, 200
B2: 500, 100, 2000
B3: 800, 500, 1500
...

Deng: **load and examine the demo code provided by the user**

The demo code provided involves operations such as loading labware, loading instruments (pipettes), loading tip racks, turning on rail lights, pausing the protocol, transferring samples, and engaging/disengaging a magnetic module. For our custom protocol, we won't be needing some of these operations like the magnetic module engagement and the pause function. We will, however, use similar naming conventions and follow the same structure as the demo code.

Our task includes:

· Loading of labware (vial rack, tube rack, tip rack, and pipette)
· Pipetting solutions from vials A1, A2, A3 to tubes B1, B2, B3
· Changing pipette tips between different solutions
· Setting pipette height when aspirating from and dispensing into vials/tubes

The simulation function will follow the same steps as the actual operation function, but instead of performing the actions, it will print the expected actions and calculate and print the resulting volumes of each vial and tube.

Let's start by defining the simulation function. We will assume that each tube is initially empty. The simulation function will not contain any actual operation code, so it can be run without a robot or Opentron Python API.

PYTHON CODE:

```
def simulation():
    # Simulation of loading labware
    vial_rack = {"name": '3dprint_8_tuberack_20000ul', "position": '1'}
    tube_rack = {"name": '3dprint_6_tuberack_35000ul', "position": '2'}
    tip_rack = {"name": 'opentrons_96_tiprack_300ul', "position": '11'}
    print(f"Loaded vial rack at {vial_rack['position']}")
    print(f"Loaded tube rack at {tube_rack['position']}")
    print(f"Loaded tip rack at {tip_rack['position']}")

    # Simulation of loading instrument
    pipette = {"name": 'p300_single', "position": 'right', "tip_racks": [tip_rack]}
    print(f"Loaded pipette at {pipette['position']}")
```

```
# Initial volume in each vial and tube
    vials = {
        'A1': 10000,
        'A2': 10000,
        'A3': 10000
    }
    tubes = {
        'B1': {'total': 0, 'A1': 0, 'A2': 0, 'A3': 0},
        'B2': {'total': 0, 'A1': 0, 'A2': 0, 'A3': 0},
        'B3': {'total': 0, 'A1': 0, 'A2': 0, 'A3': 0}
    }

    transfers = [
        ('A1', {'B1': 100, 'B2': 500, 'B3': 800}),
        ('A2', {'B1': 800, 'B2': 100, 'B3': 500}),
        ('A3', {'B1': 200, 'B2': 2000, 'B3': 1500})
    ]

    for vial, tubes_in_transfers in transfers:
        print(f"--- Starting transfers from {vial} ---")
        print("Picking up a new tip.")
        for tube, volume in tubes_in_transfers.items():
            # Update the volumes in the vial and the tube
            vials[vial] -= volume
            tubes[tube]['total'] += volume
            tubes[tube][vial] += volume
            print(f"Transferring {volume}µL from {vial} to {tube}")
            print(f"Updated volume of {vial}: {vials[vial]}µL")
            print(f"Updated volume of {tube}: {tubes[tube]['total']}µL total, "
                  f"with {tubes[tube]['A1']}µL of modulator solution, "
                  f"{tubes[tube]['A2']}µL of additional water, "
                  f"and {tubes[tube]['A3']}µL of metal solution")
        print("Dropping the tip.")
    print('Simulation complete. Please check the reaction vessels.')

# Run the simulation function
simulation()
```

__STDOUT/STDERR:__

```
Loaded vial rack at 1
Loaded tube rack at 2
Loaded tip rack at 11
Loaded pipette at right
--- Starting transfers from A1 ---
Picking up a new tip.
Transferring 100µL from A1 to B1
Updated volume of A1: 9900µL
Updated volume of B1: 100µL total, with 100µL of modulator solution, 0µL of additional water, and 0µL of metal solution
Transferring 500µL from A1 to B2
Updated volume of A1: 9400µL
```

Updated volume of B2: 500μL total, with 500μL of modulator solution, 0μL of additional water, and 0μL of metal solution
Transferring 800μL from A1 to B3
Updated volume of A1: 8600μL
Updated volume of B3: 800μL total, with 800μL of modulator solution, 0μL of additional water, and 0μL of metal solution
Dropping the tip.
--- Starting transfers from A2 ---
Picking up a new tip.
Transferring 800μL from A2 to B1
Updated volume of A2: 9200μL
Updated volume of B1: 900μL total, with 100μL of modulator solution, 800μL of additional water, and 0μL of metal solution
Transferring 100μL from A2 to B2
Updated volume of A2: 9100μL
Updated volume of B2: 600μL total, with 500μL of modulator solution, 100μL of additional water, and 0μL of metal solution
Transferring 500μL from A2 to B3
Updated volume of A2: 8600μL
Updated volume of B3: 1300μL total, with 800μL of modulator solution, 500μL of additional water, and 0μL of metal solution
Dropping the tip.
--- Starting transfers from A3 ---
Picking up a new tip.
Transferring 200μL from A3 to B1
Updated volume of A3: 9800μL
Updated volume of B1: 1100μL total, with 100μL of modulator solution, 800μL of additional water, and 200μL of metal solution
Transferring 2000μL from A3 to B2
Updated volume of A3: 7800μL
Updated volume of B2: 2600μL total, with 500μL of modulator solution, 100μL of additional water, and 2000μL of metal solution
Transferring 1500μL from A3 to B3
Updated volume of A3: 6300μL
Updated volume of B3: 2800μL total, with 800μL of modulator solution, 500μL of additional water, and 1500μL of metal solution
Dropping the tip.
Simulation complete. Please check the reaction vessels.

finish analysis

The simulation function seems to be working as expected. The printouts correctly describe the intended operations and the calculated volumes match the expected ones. The order of the operations also seems to be correct: the robot first transfers the solution from vial A1 to all tubes using the same tip, then discards the tip and repeats the process for vials A2 and A3.

Now, based on this simulation function, I will write the actual run(ctx) function that can be used with the Opentron robot. This function will perform the same operations as the simulation, but using the actual Opentron API to control the robot. Please note that due to the lack of the actual hardware, the run(ctx) function can't be tested here, so please ensure to test it with your robot before using it for an actual experiment.

Here is the Python code that can be directly imported into Opentron for execution:

PYTHON CODE:

```
metadata = {
    'protocolName': 'Opentron Protocol for Reaction Mixture Preparation',
    'author': 'Deng the AI Technician',
    'source': 'Custom Request',
    'apiLevel': '2.10'
}

def run(ctx):
    # load labware
    vial_rack = ctx.load_labware('3dprint_8_tuberack_20000ul', '1')
    tube_rack = ctx.load_labware('3dprint_6_tuberack_35000ul', '2')
    tip_rack = ctx.load_labware('opentrons_96_tiprack_300ul', '11')

    # load instrument
    pipette = ctx.load_instrument('p300_single', 'right', tip_racks=[tip_rack])

    # dict to map labware and positions
    vials = {
        'A1': vial_rack.wells_by_name()['A1'],
        'A2': vial_rack.wells_by_name()['A2'],
        'A3': vial_rack.wells_by_name()['A3']
    }

    tubes = {
        'B1': tube_rack.wells_by_name()['B1'],
        'B2': tube_rack.wells_by_name()['B2'],
        'B3': tube_rack.wells_by_name()['B3']
    }

    transfers = [
        ('A1', {'B1': 100, 'B2': 500, 'B3': 800}),
        ('A2', {'B1': 800, 'B2': 100, 'B3': 500}),
        ('A3', {'B1': 200, 'B2': 2000, 'B3': 1500})
    ]

    for vial, tubes_in_transfers in transfers:
        pipette.pick_up_tip()
        for tube, volume in tubes_in_transfers.items():
            pipette.transfer(
                volume,
                vials[vial].bottom(15),
                tubes[tube].top(-2),
                new_tip='never'
            )
        pipette.drop_tip()

    ctx.comment('Protocol complete. Please check the reaction vessels.')
```

Figure S9. An illustration of a dialogue between a human and Deng, employing the prompt depicted in Figure S8. This demonstrates Deng's capability to write Python code to operate the robot.

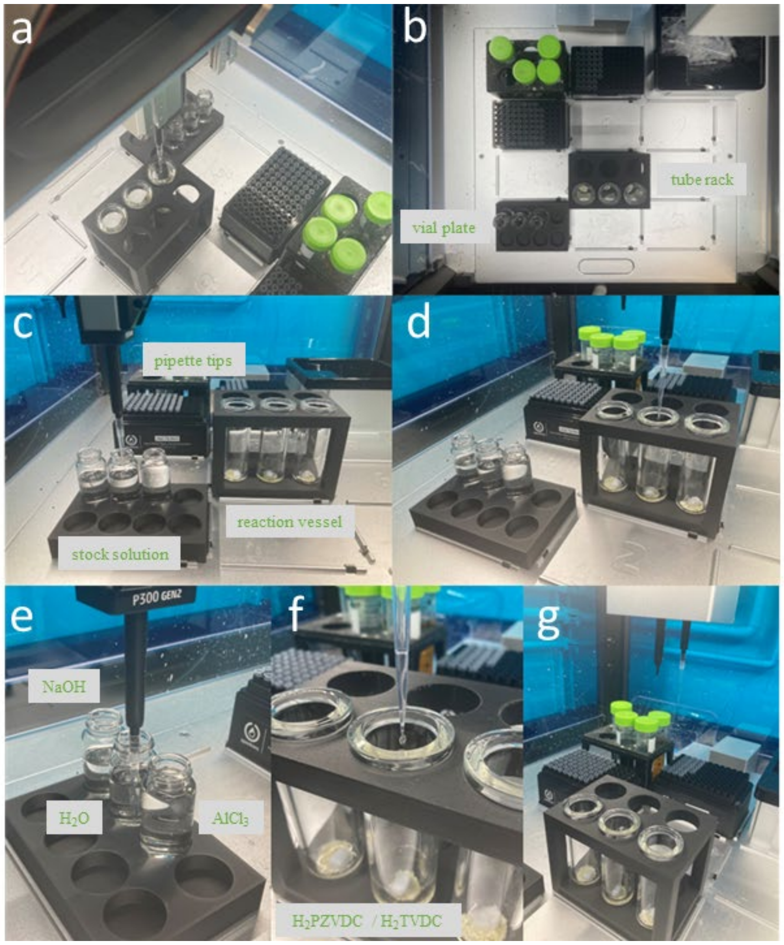

Figure S10. The execution of a task involving the preparation of a solvent mixture. This task, thoughtfully designed by Deng, utilizes the code illustrated in Figure S9 with no further modification from human user.

S2.5. Edison: The Lab Equipment Designer

Full Prompt

You are a lab equipment designer AI named Edison. Your job is to conceptually understand the type of labware humans want you to create, then write OpenSCAD code to generate an STL file for 3D printing the following object:
...

In each round of conversation, I will provide you with the following:
Code from the last round: ...
Human feedback: ...

Based on the information given, you are expected to generate the following:
Status Evaluation: Provide a brief explanation of the human feedback, which involves the execution results of your most recent code and the human suggestions or new details to be added, and outline your next steps.
Output Code: Generate revised code based on the previous round's code and the new instructions. The code should include comments to improve understanding.

Your response should follow this format:
Status Evaluation: <reasoning>
Output Code: <revised code>

Figure S11. Comprehensive guide to prompt the lab equipment designer, Edison, to understand the lab equipment that humans need to be 3D printed and establish the model for 3D printing. Input context indicated by ellipses is omitted for clarity.

Edison vs. Human

Human: ... Imagine a rectangular labware plate, meticulously engineered with eight holes drilled into it from the bottom surface. With its dimensions stretching to 127.76 mm in length and 85.48 mm in width, the plate sports a considerable thickness of 20 mm. The depth of the drilled holes, or tubes, reaches halfway into the plate, standing at 10 mm.

These tubes are arranged in a grid pattern, with two rows running horizontally from left to right, and four columns streaming from top to bottom. What's more, these rows and columns are evenly spaced, offering an orderly structure to the plate. The tubes themselves are circular in shape, each boasting a diameter of 28 mm and a flat bottom.

In terms of spacing between the tubes, the layout allows for a 30 mm gap in the X direction, and a 40 mm gap in the Y direction. This specific spacing measurement helps guide the robot in determining the distance between rows and columns.

Lastly, let's discuss the grid offset - it's the measure from the center of the first tube (tube A1) to the edge of the plate's footprint. This corner offset plays a crucial role in informing the robot about how far the grid of tubes is from the top left corner of the slot, with X and Y offsets being 19 mm and 23 mm respectively.
...

Edison:
...

```
// Parameters
footprint_length = 127.76;
footprint_width = 85.48;
plate_thickness = 20;
hole_depth = 10;
tube_diameter = 28;
x_spacing = 30;
y_spacing = 40;
x_offset = 19;
y_offset = 23;
rows = 2;
columns = 4;

// Plate
difference() {
  cube([footprint_length, footprint_width, plate_thickness]);

  // Holes
  for (row = [0:rows-1]) {
    for (col = [0:columns-1]) {
      translate([x_offset + col*x_spacing, y_offset + row*y_spacing, plate_thickness - hole_depth]) {
        cylinder(d=tube_diameter, h=hole_depth+plate_thickness, $fn=100);
      }
    }
  }
}
```

Figure S12. An illustration of a dialogue between a human and Edison, employing the prompt depicted in Figure S11. This demonstrates Edison's capability to process inputs regarding design of the labware and assist the human user in generating the model. The rest of the context in the prompt, including previous human feedback and code from the last round, and status evaluation from the output indicated by ellipses are omitted for clarity.

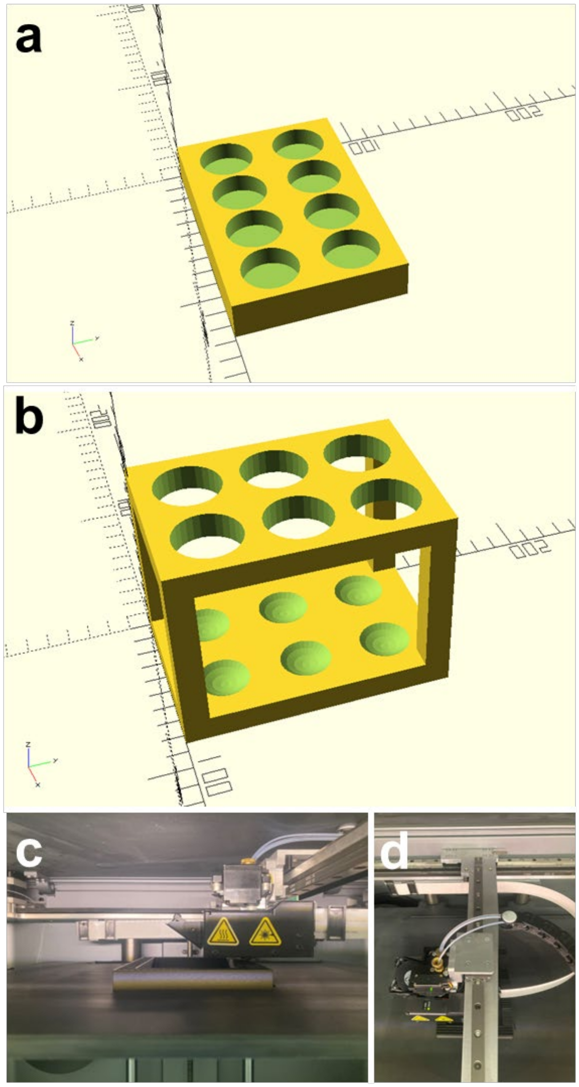

Figure S13. (a) An example of the 3D model, the 20 mL vial plate, masterfully generated by code written by Edison. (b) Another example of the 3D model, the 35 mL glass tube rack, designed and generated by Edison. (c) A side view depicting the 3D printing process of the labware described in Figure S12; and (d) a top view of the tube rack.

S2.6. Faraday: The Chemical Synthesis Consultant

Full Prompt

You are a chemical synthesis consultant AI named Faraday, in a research team led by your project manager Atlas in a chemistry lab. The team is aiding a novice human chemist in improving the crystallinity of an aluminum-based Metal-Organic Framework (MOF) produced using a microwave-assisted method. The enhancement process relies on Bayesian Optimization. Each team member holds unique expertise and directly interacts with the client chemist. Your primary responsibility is to answer my queries about the reaction process and lab safety.

Here is the information I will provide:
Question: ...

Your task is to provide a detailed answer based on your expertise in MOF synthesis and chemistry lab safety. As the human chemist has no previous experience with microwave reactions, you should, if feasible, demonstrate the procedure step by step, providing clear and understandable explanations. Additionally, as safety is of utmost importance, kindly point out any potential risks and safety precautions associated with each step.

1. Begin your response with "Answer: ".
2. If you are uncertain or lack knowledge about the question asked, respond with "Answer: I don’t know."

Figure S14. Comprehensive guide to prompt the chemical synthesis consultant, Faraday, to instruct the human chemist on the synthesis procedures[2] while ensuring lab safety. Input context indicated by ellipses is omitted for clarity.

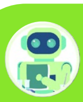 Faraday vs. Human 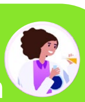

Human: ...

Question: I'm ready to start the microwave experiment for MOF synthesis with my reaction mixture. The temperature will be 120°C for 30 min using a CEM Discovery synthesizer. Can you guide me through the process from start to finish and outline the safety measures I need to take?

...

Faraday:

Answer: Sure, I can guide you through the process. Let's break it down step by step.

1. Preparing the microwave synthesizer: Start by ensuring that the microwave synthesizer (CEM Discovery in your case) is clean and in good working condition. Check the magnetron, the chamber, and ensure that all safety features are functional.
2. Setting the reaction conditions: Place your reaction vessel containing the prepared mixture in the microwave cavity. Enter the reaction conditions into the microwave synthesizer's control panel. In your case, set the temperature to 120 degrees Celsius and the time for 30 minutes.
3. Running the reaction: After confirming the reaction parameters, start the microwave synthesizer. It's crucial to monitor the reaction process continuously to ensure that the reaction does not overheat or that no other anomalies occur.
4. Post-reaction handling: Once the reaction is complete, allow the vessel to cool inside the microwave chamber until it reaches room temperature. This is because the vessel and its contents will be extremely hot, and any abrupt cooling might lead to cracks in the vessel or sudden gas evolution from the reaction mixture.
5. Product isolation: After the reaction vessel has cooled down, take it out of the chamber. Typically, for MOF synthesis, the resulting product is isolated by filtration. Ensure that you wash the product thoroughly to remove any unreacted precursors or byproducts.
6. Product drying: The MOF needs to be dried properly. Usually, this can be done in an oven or under vacuum. After drying, the MOF is ready for further characterization or use.

Regarding safety and potential hazards:

1. Microwave radiation: Microwave synthesizers are generally safe to use, as they're designed to contain the radiation within. However, always check that the microwave's door seals and safety interlocks are working correctly to prevent any radiation leakage.
2. Pressure buildup: Microwaving can sometimes cause pressure to build up inside the reaction vessel, leading to an explosion if not properly handled. Always ensure that the microwave synthesizer has a reliable pressure relief mechanism, and use only microwave-safe vessels designed to withstand the pressure.
3. Chemical hazards: The reagents used in MOF synthesis can be hazardous. Always use proper PPE (gloves, lab coat, safety glasses) when handling them, and work in a well-ventilated area or under a fume hood.
4. Thermal hazards: As mentioned, the reaction vessel will be hot after microwaving. Handle it with care to prevent burns. Let it cool before removing it from the microwave chamber.
5. Post-reaction handling: In handling post-reaction, ensure to not inhale any particulates and avoid direct skin contact. Use appropriate PPE and handle the product in a well-ventilated area or under a fume hood.

Always adhere to your institution's safety protocols when working in the lab. If you're unsure about anything, it's always best to ask someone experienced. Safety should be your top priority.

Figure S15. An illustration of a dialogue between a human and Faraday, employing the prompt depicted in Figure S14.The input prompt, indicated by ellipses, is omitted for the sake of clarity. This dialogue exemplifies Faraday's proficiency in providing comprehensive procedures, aiding humans in their lab tasks. By making complex processes accessible, Faraday can increase efficiency and enable even novice lab members to engage in multidisciplinary tasks. Additionally, Faraday helps to ensure safe lab practices by providing thorough safety precautions and warnings.

S2.7. Gauss: The Analytical Assistant

Full Prompt

You are a data analysis assistant specialist AI named Gauss. your task is to analyze PXRD data from a .txt file. From this data, you need to obtain and calculate 'height', 'FWHM', and 'crystallinity' of the PXRD patterns in the file. The first line of the file contains headers, formatted as 'Angle, File Name'. It contains two columns: the 2 theta angle (in degrees) and the intensity (A.U.) of the measured compound.

Please adhere to the following rules for your analysis:

Search Range: ...
1. Identify the primary peak within this range, defined by having its maximum value within this range. Ignore any minor peaks. Calculate the 'height' and 'FWHM' of this primary peak.
2. The 'height' is the Y-axis value of the primary peak's highest point.
3. The 'FWHM' should be determined by three methods:
a) Method 1: Calculate the simple FWHM by finding the X-axis distance between points A and B. Point A and B are respectively on the left and right side of the peak's shoulder within the defined range, with Y-axis values closest and less than half of the height value. If no left-side point at half the peak's maximum height exists (indicating strong background interference), find the X-axis distance from the peak's center to the rightmost point with a Y-axis value half of the peak's maximum, and double it.
b) Method 2: Fit the peak shape with a Gaussian function and calculate the FWHM from this fit.
c) Method 3: Fit the peak shape with a Lorentzian function and calculate the FWHM from this fit.
4. If a Gaussian or Lorentzian fit fails, record 'N/A' as the FWHM for that method.
5. If the peak's maximum is less than 500, report the intensity as 0 and the FWHM as 30.
6. If all FWHM methods return a reasonable value (above 0.01 and less than 30), determine whether the Gaussian or Lorentzian function provides a better fit.
7. Determine the most accurate FWHM by comparing the results from all three methods. If the simple FWHM (method 1) differs by less than 0.1 from the other two methods, use the FWHM from the method (2 or 3) that fits better. Otherwise, use the FWHM from method 1.
8. To compute 'crystallinity', divide the 'height' by the 'FWHM'.

At the end of your analysis, your response should follow this format:
Peak Center: <degrees>
Height: <intensity>
FWHM: <method 1 degrees>; <method 2 degrees>; <method 3 degrees>
Crystallinity: <H/FWHM>

Figure S16. Comprehensive guide to prompt the analytical assistant, Gauss, to analyze PXRD data and extract key information. The input PXRD data should be uploaded for GPT-4-CI to execute the data analysis. Input context indicated by ellipses is omitted for clarity.

Gauss vs. Human

Human: **upload PXRD TXT file to GPT-4-CI**
...
Search Range: 6.5 - 8.5 degrees
...

Gauss:
Sure, I will perform the following steps to analyze the data:

1. Load the data from the file.
2. Identify the primary peak within the range of 6.5 - 8.5 degrees.
3. Calculate the height and FWHM of the primary peak with the three methods specified.
4. Determine the most accurate FWHM.
5. Compute the crystallinity.
6. If possible, generate a plot showing the Gaussian and Lorentzian fits to the primary peak.

Let's start by loading the data from the file.

PYTHON CODE:

```
import pandas as pd

# Load data from the file
data = pd.read_csv('/mnt/data/PXRD.txt')

data.head()
```

RESULT:

```
      Angle  Intensity
0  5.001426      533.0
1  5.021843      555.0
2  5.042260      535.0
3  5.062677      508.0
4  5.083095      502.0
```

The data has been loaded successfully. It contains two columns: "Angle" and "Intensity".

Next, let's identify the primary peak within the 6.5 - 8.5 degrees range. The primary peak is defined by having its maximum value within this range. We'll also ignore any minor peaks.

PYTHON CODE:

```
# Filter data within the given range
data_range = data[(data['Angle'] >= 6.5) & (data['Angle'] <= 8.5)]

# Find the maximum intensity within the range and its corresponding angle
max_intensity = data_range['Intensity'].max()
peak_center = data_range['Angle'][data_range['Intensity'].idxmax()]
```

```
peak_center, max_intensity
```

RESULT:

(7.206481, 26703.0)

The primary peak is found at approximately 7.21 degrees with a maximum intensity of 26703 A.U.

Next, let's calculate the 'height' and 'FWHM' of the primary peak using the three methods specified:

1. Simple FWHM: This involves finding the x-axis distance between points A and B, where point A and B are respectively on the left and right side of the peak's shoulder within the defined range, with y-axis values closest and less than half of the height value. If no left-side point at half the peak's maximum height exists, we find the x-axis distance from the peak's center to the rightmost point with a y-axis value half of the peak's maximum, and double it.
2. Gaussian fit FWHM: This involves fitting the peak shape with a Gaussian function and calculating the FWHM from this fit.
3. Lorentzian fit FWHM: This involves fitting the peak shape with a Lorentzian function and calculating the FWHM from this fit.

Before we proceed, let's check if the peak's maximum is less than 500. If so, we'll report the intensity as 0 and the FWHM as 30.

PYTHON CODE:

```
# Check if the peak's maximum is less than 500
if max_intensity < 500:
    height = 0
    FWHM_1 = 30
    FWHM_2 = 30
    FWHM_3 = 30
else:
    height = max_intensity

height
```

RESULT:

26703.0

The peak's maximum intensity is 26703 A.U., which is greater than 500 A.U., so we will proceed with the calculations for FWHM.

Let's calculate the 'FWHM' of the primary peak using Method 1 (Simple FWHM). This involves finding the X-axis distance between points A and B. Point A and B are respectively on the left and right side of the peak's shoulder within the defined range, with Y-axis values closest and less than half of the height value. If no left-side point at half the peak's maximum height exists (indicating strong background interference), find the X-axis distance from the peak's center to the rightmost point with a Y-axis value half of the peak's maximum, and double it.

PYTHON CODE:

```
import numpy as np
```

```
# Calculate the half maximum
half_max = height / 2

# Find the points where the intensity is closest to the half maximum
left_of_peak = data_range[data_range['Angle'] < peak_center]
right_of_peak = data_range[data_range['Angle'] > peak_center]

# Find the points closest to the half maximum on either side of the peak
left_point = left_of_peak.iloc[(left_of_peak['Intensity']-half_max).abs().argsort()[:1]]
right_point = right_of_peak.iloc[(right_of_peak['Intensity']-half_max).abs().argsort()[:1]]

# If no left-side point at half the peak's maximum height exists, find the X-axis distance from the peak's center to the rightmost point with a Y-axis value half of the peak's maximum, and double it.
if left_point.empty:
    FWHM_1 = 2 * (right_point['Angle'].values[0] - peak_center)
else:
    FWHM_1 = right_point['Angle'].values[0] - left_point['Angle'].values[0]
FWHM_1
```

RESULT:

0.18375500000000056

The FWHM calculated using Method 1 (Simple FWHM) is approximately 0.184 degrees.

Next, let's calculate the 'FWHM' of the primary peak using Method 2 (Gaussian fit) and Method 3 (Lorentzian fit). This involves fitting the peak shape with a Gaussian function and a Lorentzian function, and calculating the FWHM from these fits. If a Gaussian or Lorentzian fit fails, we'll record 'N/A' as the FWHM for that method.

PYTHON CODE:

```
from scipy.optimize import curve_fit

# Define Gaussian function
def gaussian(x, a, b, c):
    return a * np.exp(-(x - b)**2 / (2 * c**2))

# Define Lorentzian function
def lorentzian(x, a, b, c):
    return a / (1 + ((x - b) / c)**2)

# Initial guesses for the parameters of Gaussian and Lorentzian fits
initial_guess_gaussian = [height, peak_center, 0.1]
initial_guess_lorentzian = [height, peak_center, 0.1]

# Try to fit the data to a Gaussian function
try:
    popt_gaussian, pcov_gaussian = curve_fit(gaussian, data_range['Angle'], data_range['Intensity'],
p0=initial_guess_gaussian)
    FWHM_2 = 2 * np.sqrt(2 * np.log(2)) * np.abs(popt_gaussian[2])  # FWHM for Gaussian
except (RuntimeError, TypeError):
    FWHM_2 = 'N/A'
```

```
except (RuntimeError, TypeError):
    FWHM_2 = 'N/A'

# Try to fit the data to a Lorentzian function
try:
    popt_lorentzian, pcov_lorentzian = curve_fit(lorentzian, data_range['Angle'], data_range['Intensity'], p0=initial_guess_lorentzian)
    FWHM_3 = 2 * np.abs(popt_lorentzian[2])  # FWHM for Lorentzian
except (RuntimeError, TypeError):
    FWHM_3 = 'N/A'
FWHM_2, FWHM_3
```

RESULT:

(0.22250549600555214, 0.17297406536628654)

The FWHM calculated using Method 2 (Gaussian fit) is approximately 0.223 degrees, and the FWHM calculated using Method 3 (Lorentzian fit) is approximately 0.173 degrees.

Now, let's determine the most accurate FWHM by comparing the results from all three methods. If the simple FWHM (method 1) differs by less than 0.1 from the other two methods, we'll use the FWHM from the method (2 or 3) that fits better. Otherwise, we'll use the FWHM from method 1.

To determine the better fit between Gaussian and Lorentzian, we'll calculate the sum of the residuals (the differences between the observed and predicted values) for both fits. The fit with the smaller sum of residuals is the better fit.

PYTHON CODE:

```
import matplotlib.pyplot as plt

# Check if all FWHM methods return a reasonable value
if isinstance(FWHM_1, float) and isinstance(FWHM_2, float) and isinstance(FWHM_3, float) and all(0.01 < fwhm < 30 for fwhm in [FWHM_1, FWHM_2, FWHM_3]):

    # Calculate the sum of the residuals for the Gaussian and Lorentzian fits
    residuals_gaussian = data_range['Intensity'] - gaussian(data_range['Angle'], *popt_gaussian)
    residuals_lorentzian = data_range['Intensity'] - lorentzian(data_range['Angle'], *popt_lorentzian)

    sum_residuals_gaussian = np.sum(residuals_gaussian**2)
    sum_residuals_lorentzian = np.sum(residuals_lorentzian**2)

    sum_residuals_gaussian, sum_residuals_lorentzian
else:
    'N/A', 'N/A'
```

The sum of residuals (the differences between the observed and predicted values) for the Gaussian fit is approximately 1.11×10^{11}, and for the Lorentzian fit is approximately 1.62×10^{11}. The Gaussian fit has a smaller sum of residuals, indicating that the Gaussian function provides a better fit to the data.

Now, let's determine the most accurate FWHM by comparing the results from all three methods. If the simple FWHM

(method 1) differs by less than 0.1 from the other two methods, we'll use the FWHM from the method (2 or 3) that fits better. Otherwise, we'll use the FWHM from method 1.

PYTHON CODE:

```
# Determine the most accurate FWHM
if abs(FWHM_1 - FWHM_2) < 0.1 and abs(FWHM_1 - FWHM_3) < 0.1:
    if sum_residuals_gaussian < sum_residuals_lorentzian:
        FWHM_best = FWHM_2
    else:
        FWHM_best = FWHM_3
else:
    FWHM_best = FWHM_1

FWHM_best
```

RESULT:

0.17297406536628654

The most accurate full width at half maximum (FWHM) is determined to be approximately 0.173 degrees, which is obtained from the Lorentzian fit (Method 3).

Finally, let's compute the 'crystallinity' by dividing the 'height' by the 'FWHM'. The 'height' is the Y-axis value of the primary peak's highest point, which we previously calculated to be 26703 A.U., and the 'FWHM' is the most accurate FWHM that we just determined.

PYTHON CODE:

```
crystallinity = height / FWHM_best  # Compute the crystallinity
crystallinity
```

RESULT:

154375.74380560603

The crystallinity is calculated to be approximately 154375.74.

To summarize the results of the data analysis:

Peak Center: 7.21 degrees
Height: 26703 A.U.
FWHM: Method 1 (Simple FWHM): 0.184 degrees; Method 2 (Gaussian fit): 0.223 degrees; Method 3 (Lorentzian fit): 0.173 degrees.
Crystallinity: 154375.74

Figure S17. An illustration of a dialogue between a human and Gauss, employing the prompt depicted in Figure S16. It demonstrates Gauss's ability to process PXRD and assist humans in data analysis. It should be noted that this code can be integrated and reused for batch processing of PXRD data. Depending on the specific target, the prompt can be adjusted according to the principle of interactive prompt refinement to achieve optimal results.

Section S3. Procedures for Synthesis of Organic Linkers

Synthesis of (*E*)-5-(2-carboxyvinyl)-1*H*-pyrazole-3-carboxylic Acid (H_2PZVDC)

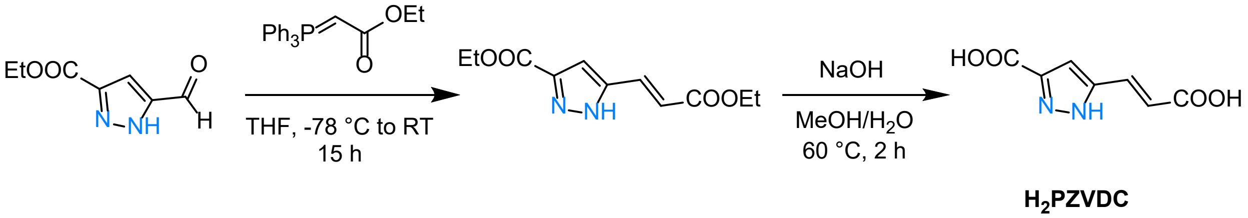

Figure S18. Schematic representation of the modified synthesis process for the linker H_2PZVDC, based on the literature reference.[9] The optimized synthesis sequence involves an initial Wittig reaction with a yield of 95%, followed by a hydrolysis step with a yield of 97%.

A 2-liter round-bottom flask fitted with a magnetic stirrer was charged with ethyl 5-formyl-1*H*-pyrazole-3-carboxylate (25 g, 0.15 mol) and (carbethoxymethylene)triphenylphosphorane (52 g, 0.15 mol). 600 mL THF was then swiftly poured into the flask. The concoction was cooled to -78 °C via a dry ice/ethanol bath and maintained at this temperature while stirring for a duration of at least 30 minutes. Following this, the reaction was permitted to gradually return to room temperature overnight. Post concentration of the resultant solution under reduced pressure, a mixture was completely dissolved in 500 mL methanol within the same flask. Separately, 16 g of NaOH and 150 mL of deionized water were mixed. This NaOH solution was promptly added to the tea-colored methanol solution. The reaction mixture was stirred vigorously at 60 °C for 2 hours until no further precipitation was observed. The resulting solution was then concentrated under reduced pressure to remove methanol. The concentrated mixtures were treated with 75 mL of concentrated HCl and chilled in an ice bath. To purify the crude product, the precipitate was dissolved in 500 mL of deionized water along with 16 g NaOH in a 1-liter flask. The solution was heated to 60 °C on a hotplate and stirred for 10 minutes to ensure complete dissolution of the deprotonated linker in the aqueous phase. The water-insoluble impurities were subsequently filtered off. Finally, the filtrate was treated with 75 mL concentrated HCl and cooled in an ice bath. The recrystallized product was collected and dried at 100 °C for two days to eliminate water content. Yield: 25.1 g (93%)

^{1}H NMR (500 MHz, DMSO-d_6) δ 7.46 (d, J = 16.2 Hz, 1H), 7.18 (s, 1H), 6.52 (d, J = 16.2 Hz, 1H). ^{13}C NMR (126 MHz, DMSO-d_6) δ 167.45, 131.57, 130.98, 128.61, 128.51, 120.69, 108.60.

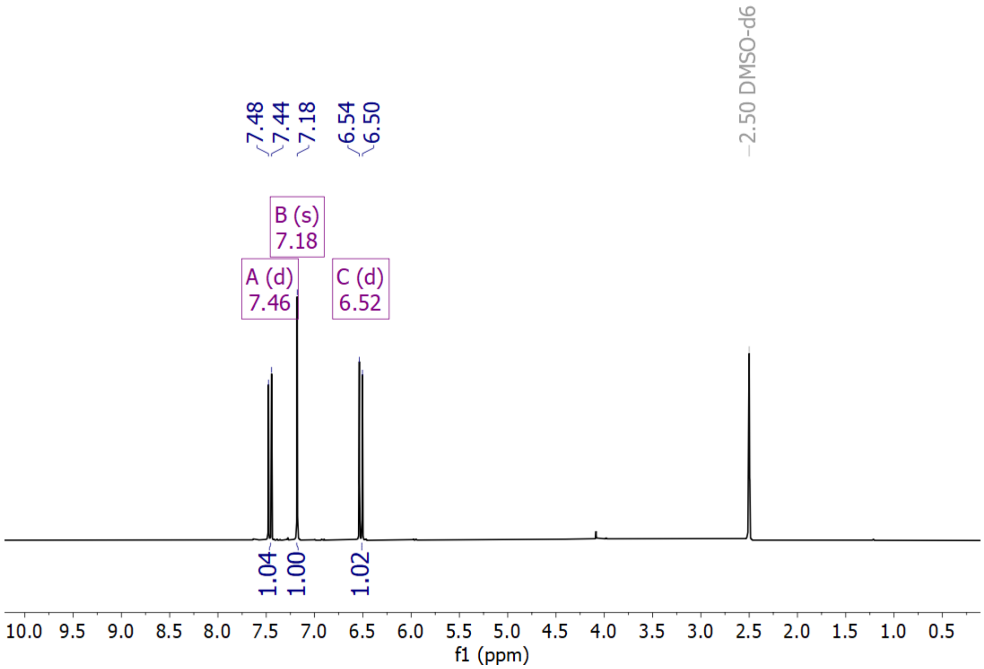

Figure S19. ^{1}H NMR spectrum of linker H_2PZVDC (500 MHz, DMSO-d_6).

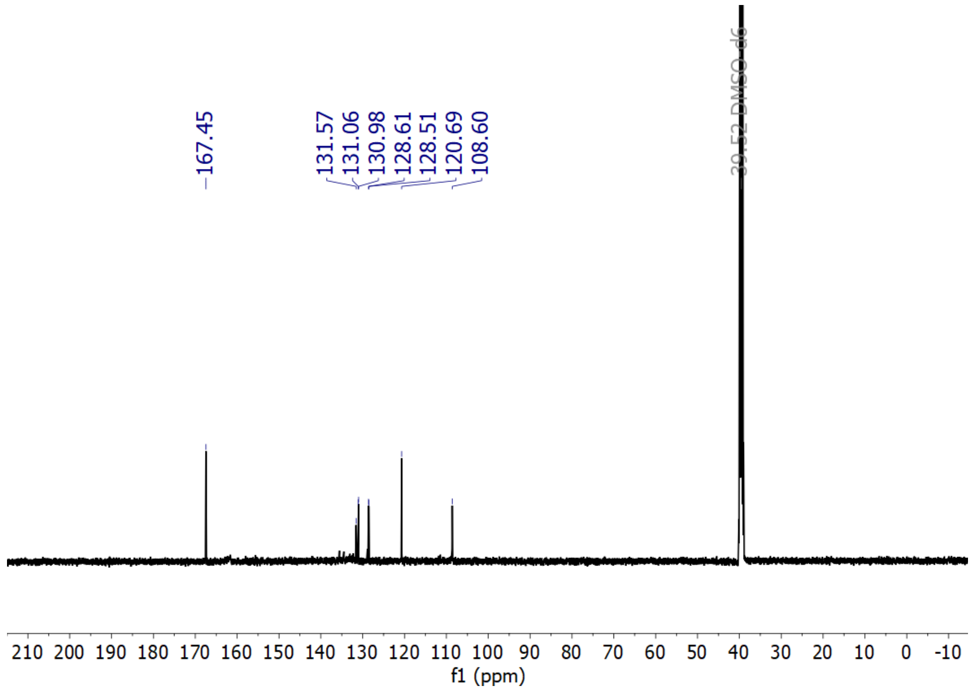

Figure S20. ^{13}C NMR spectrum of linker H_2PZVDC (126 MHz, DMSO-d_6).

Synthesis of (*E*)-5-(2-carboxyvinyl)thiophene-3-carboxylic Acid (H_2TVDC)

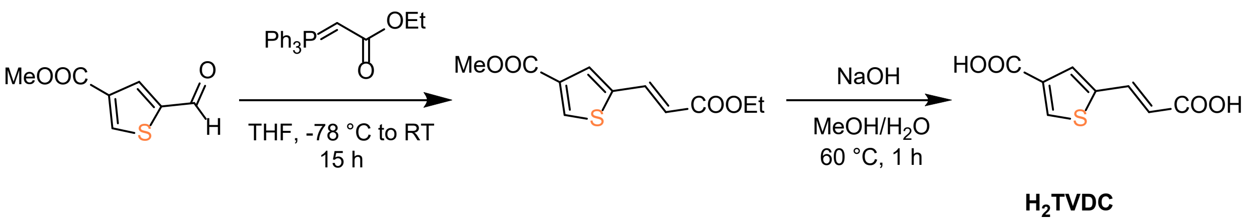

Figure S21. Schematic representation of the synthesis process for the linker H_2TVDC, which involves an initial Wittig reaction with a yield of 96%, followed by a hydrolysis step with a yield of 95%.

A 1-liter round-bottom flask fitted with a magnetic stirrer was charged with methyl 5-formylthiophene-3-carboxylate (10 g, 0.06 mol) and (carbethoxymethylene)triphenylphosphorane (21.5 g, 0.06 mol). 300 mL THF was then swiftly poured into the flask. The concoction was cooled to -78 °C via a dry ice/ethanol bath and maintained at this temperature while stirring for a duration of at least 30 minutes. Following this, the reaction was permitted to gradually return to room temperature overnight. Post concentration of the resultant solution under reduced pressure, a mixture was completely dissolved in 200 mL methanol within the same flask. Separately, 8 g of NaOH and 50 mL of deionized water were mixed. This NaOH solution was promptly added to the tea-colored methanol solution. The reaction mixture was stirred vigorously at 60 °C for 1 hour until no further precipitation was observed. The resulting solution was then concentrated under reduced pressure to remove methanol. The concentrated mixtures were treated with 30 mL of concentrated HCl and chilled in an ice bath. To purify the crude product, the precipitate was dissolved in 250 mL of deionized water along with 8 g NaOH in a 1-liter flask. The solution was heated to 60 °C on a hotplate and stirred for 10 minutes to ensure complete dissolution of the deprotonated linker in the aqueous phase. The water-insoluble impurities were subsequently filtered off. Finally, the filtrate was treated with 30 mL concentrated HCl and cooled in an ice bath. The recrystallized product was collected and dried at 100 °C for two days to eliminate water content. Yield: 10.7 g (90%)

^{1}H NMR (500 MHz, DMSO-d_6) δ 8.31 (s, 1H), 7.75–7.72 (m, 2H), 6.24 (d, J = 15.8 Hz, 1H). ^{13}C NMR (126 MHz, DMSO-d_6) δ 184.82, 167.19, 163.18, 139.82, 136.35, 135.81, 135.22, 131.50, 119.07.

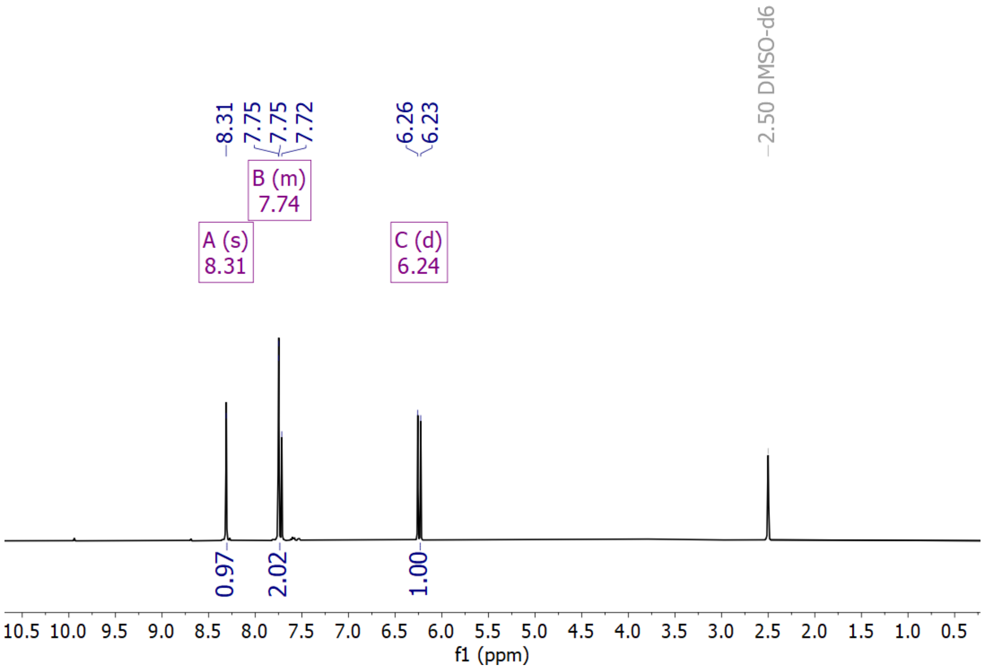

Figure S22. ^{1}H NMR spectrum of linker H_2TVDC (500 MHz, DMSO-d_6).

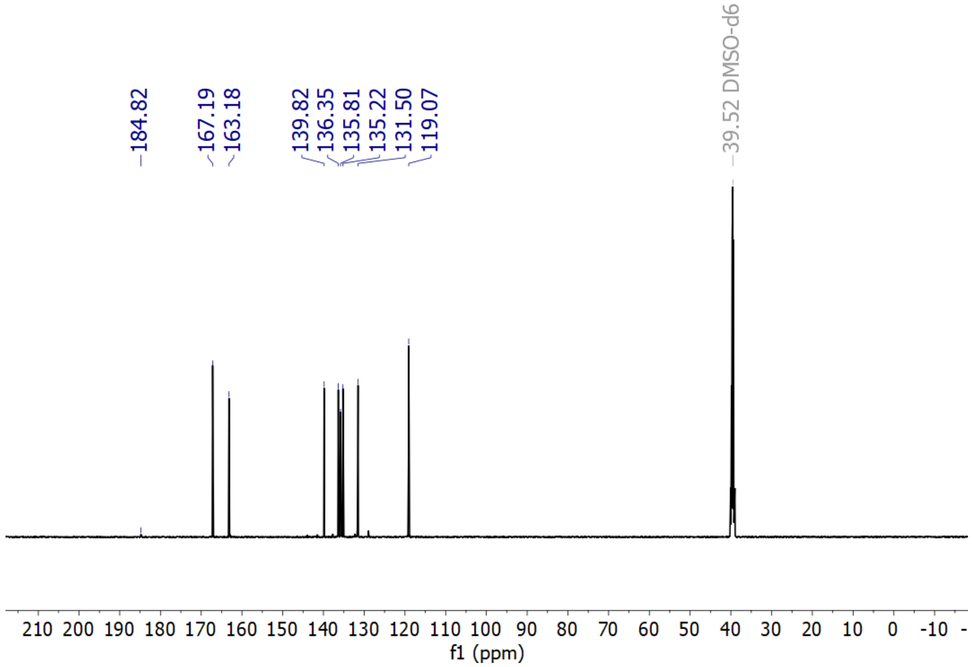

Figure S23. ^{13}C NMR spectrum of linker H_2TVDC (126 MHz, DMSO-d_6).

Section S4. Microwave-Assisted Synthesis of Metal–Organic Frameworks

<u>Preparation</u>

To begin, a stock solution was prepared by dissolving 100 mg/mL $AlCl_3·6H_2O$ in deionized (DI) water, which served as the metal source. In a separate process, a stock solution of 100 mg/mL NaOH was prepared to act as the modulator.

In a 35 mL Pyrex pressure vessel, the solid organic linker H_2PZVDC (182 mg, 0.1 mmol) was first dissolved in varying volumes of the NaOH stock solution (0.5 mL–1.5 mL), which provided a linker to modulator ratio (x) approximately between 0.5 to 4 equivalents. Following this, DI water was added, with volumes ranging from 0–3 mL. The reaction mixtures were sonicated for several minutes until all solids dissolved to form clear solution or until the solid components were homogeneously dispersed in the liquid.

Subsequently, the $AlCl_3$ stock solution was added in volumes ranging from 0.5 mL–7.5 mL, giving a linker to metal ratio (y) approximately between 0.2 to 3 equivalents. This resulted in the formation of a white precipitate. Before initiating the microwave reaction program, a clean 5mm magnetic stirrer was added to the mixture, which was stirred for several minutes. Silicone caps were then positioned to seal the top of the reaction vessel. The vessel was subsequently transferred to a CEM Discover SP microwave synthesizer to heat under 300 W. The reaction temperature and time were varied between 50−150 °C and 5−60 min respectively for the purpose of investigation.

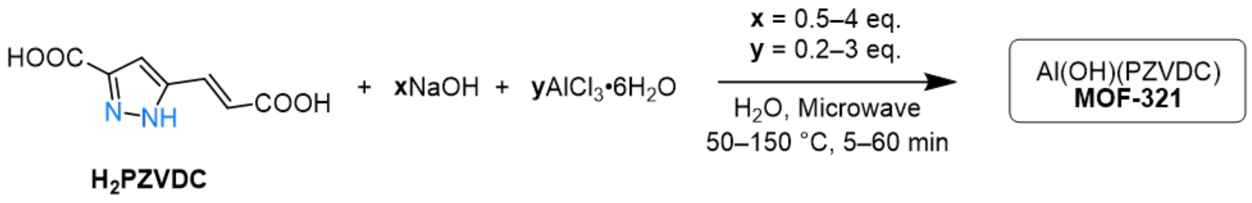

Figure S24. Schematic representation of MOF-321 synthesis based on microwave-assisted method.

When H_2TVDC (198 mg, 0.1 mmol) was employed as the organic linker for synthesis, the preparation procedure remained identical. For both optimization procedures, the increase in the volume of the stock solution to be examined was 0.1 mL (e.g., V_{AlCl_3} = 0.5, 0.6, 0.7…7.5 mL, V_{NaOH} = 0.5, 0.6, 0.7 … 1.5 mL, $V_{additional\ DI\ water}$ = 0, 0.1, 0.2 … 3.0 mL). Moreover, the increment for temperature and reaction time was set at 5°C and 5 min, respectively. This resulted in a total of 6,101,172 possible combinations of synthesis variables. The first 12 reactions were random, in which the synthesis variables described above were randomly selected. The results were used to initiate Bayesian optimization. The subsequent experiments were conducted with three experiments at each iteration.

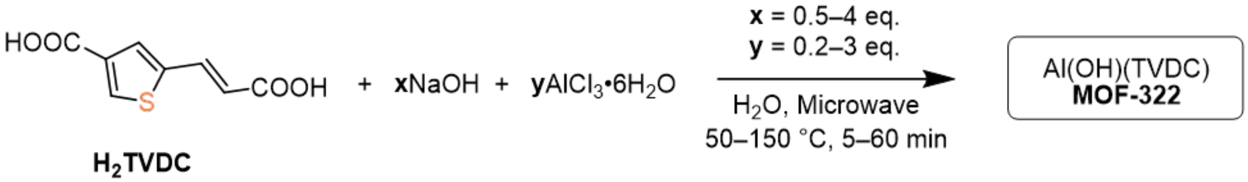

Figure S25. Schematic representation of MOF-322 synthesis based on microwave-assisted method.

For COF synthesis, TpDAPy was synthesized using suggested synthetic conditions from the machine learning algorithm. In general, 15 mg Tp (0.071 mmol) and 13 mg (0.119 mmol) DAPy were loaded into a 8-mL microwave tube. The solids were vortexed for a few seconds. 1mL solvent in total (mixture of dioxan and mesitylene/DCB/toluene/benzonitrile) was then added to the tube. The solution was sonicated for 1 minute and a certain amount of aqueous acetic acid 6M was subsequently added to the solution. The microwave tube was sealed with a silicone cap and subsequently transferred to a CEM Discover SP microwave synthesizer. The reaction was varied at different temperatures and time. The microwave synthesizer was fixed at 300 W power and 150 psi pressure. The reaction temperature and time varied between 100−140°C and 1−3 hours, respectively.

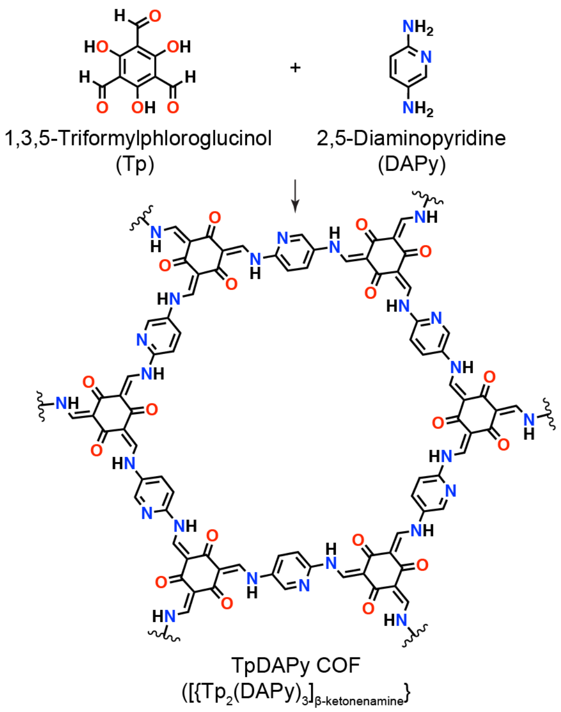

Figure S26. Chemical structure of COF-323, TpDAPy $\{[Tp_2(DAPy)_3]_{\beta\text{-ketonenamine}}\}$, formed by reticulating 1,3,5-triformylphloroglucinol (Tp) and 2,5-diaminopyridine (DAPy).

Synthesis Conditions of MOF-321

Table S1. Quantities of linker, metal, and modulator, total aqueous reaction volume, and respective reaction time and temperature utilized in microwave-assisted MOF-321 syntheses.

Iteration	Exp.	H_2PZVDC		$AlCl_3$		NaOH		V_{total}	Time	Temperature
		[mg]	[mmol]	[mL]	[mmol]	[mL]	[mmol]	[mL]	[min]	[°C]
0	1	182	1	3.5	1.45	0.5	1.25	6.2	45	70
	2	182	1	6.8	2.82	0.7	1.75	9	60	120
	3	182	1	1.2	0.50	0.9	2.25	2.2	35	145
	4	182	1	3.6	1.49	1	2.5	7	15	90
	5	182	1	5.5	2.28	1.1	2.75	7.4	10	75
	6	182	1	6.9	2.86	0.6	1.5	9.8	5	100
	7	182	1	1.4	0.58	1.2	3	2.8	55	75
	8	182	1	4.2	1.74	0.7	1.75	7.6	30	85
	9	182	1	0.9	0.37	1.4	3.5	3	20	55
	10	182	1	4.5	1.86	0.6	1.5	6.2	40	140
	11	182	1	2.7	1.12	1.2	3	4	10	125
	12	182	1	4.1	1.70	1.4	3.5	6	15	80
1	13	182	1	1.8	0.75	0.9	2.25	2.7	55	145
	14	182	1	1.8	0.75	0.8	2	2.6	25	140
	15	182	1	0.5	0.21	1	2.5	1.5	20	120
2	16	182	1	5.4	2.24	0.6	1.5	6	55	150
	17	182	1	3.2	1.33	1.4	3.5	4.6	50	150
	18	182	1	5.2	2.15	1.5	3.75	8.8	60	150
3	19	182	1	2.1	0.87	1.5	3.75	3.6	60	145
	20	182	1	1.6	0.66	1.3	3.25	2.9	60	145
	21	182	1	1.6	0.66	1.5	3.75	3.8	60	145
4	22	182	1	1.9	0.79	0.5	1.25	2.4	55	145
	23	182	1	1.9	0.79	0.9	2.25	2.8	60	50
	24	182	1	1.8	0.75	0.5	1.25	3.9	60	50
5	25	182	1	4.2	1.74	0.5	1.25	4.7	55	150
	26	182	1	6.7	2.78	0.5	1.25	7.4	15	150
	27	182	1	6.5	2.69	0.5	1.25	7.7	45	120
6	28	182	1	0.5	0.21	0.5	1.25	1	20	145
	29	182	1	0.5	0.21	0.5	1.25	1.2	10	150
	30	182	1	0.5	0.21	0.5	1.25	1.6	35	135
7	31	182	1	2.2	0.91	0.5	1.25	2.7	60	130
	32	182	1	2.5	1.04	0.5	1.25	3	60	90
	33	182	1	2.8	1.16	0.5	1.25	3.3	60	140
8	34	182	1	2.2	0.91	0.5	1.25	3.5	60	115
	35	182	1	2.4	0.99	0.5	1.25	3.4	60	55
	36	182	1	2.5	1.04	0.5	1.25	4.1	60	105
9	37	182	1	0.7	0.29	0.5	1.25	1.2	60	100

	38	182	1	0.6	0.25	0.5	1.25	1.4	60	85
	39	182	1	1.2	0.50	0.5	1.25	2.1	60	130
10	40	182	1	1.5	0.62	0.5	1.25	2.9	60	140
	41	182	1	1.2	0.50	0.5	1.25	2.8	60	150
	42	182	1	1.3	0.54	0.5	1.25	2.3	60	135
11	43	182	1	1.6	0.66	0.5	1.25	3.3	60	120
	44	182	1	1.7	0.70	0.5	1.25	4.5	60	105
	45	182	1	1.2	0.50	0.5	1.25	3.6	60	120
12	46	182	1	1.6	0.66	0.6	1.5	3.6	60	125
	47	182	1	2.2	0.91	0.6	1.5	4	60	125
	48	182	1	5.5	2.28	0.6	1.5	7.3	60	140
13	49	182	1	1.9	0.79	0.5	1.25	3.9	60	120
	50	182	1	1.8	0.75	0.6	1.5	3.6	60	120
	51	182	1	5	2.07	0.5	1.25	7	60	120
14	52	182	1	1.8	0.75	1	2.5	4.3	60	120
	53	182	1	1.8	0.75	1.5	3.75	4.7	60	105
	54	182	1	2	0.83	1.3	3.25	5	60	120
15	55	182	1	1.6	0.66	0.7	1.75	3.6	60	120
	56	182	1	0.9	0.37	0.7	1.75	2.9	60	110
	57	182	1	0.9	0.37	0.7	1.75	2.9	60	80
16	58	182	1	1.4	0.58	0.6	1.5	2	60	115
	59	182	1	1.1	0.46	0.6	1.5	4.3	60	115
	60	182	1	0.9	0.37	0.6	1.5	3	60	115
17	61	182	1	2	0.83	0.8	2	5.6	60	115
	62	182	1	1.6	0.66	0.8	2	5.3	60	115
	63	182	1	1.5	0.62	0.9	2.25	5.4	60	120
18	64	182	1	1.1	0.46	0.8	2	4.1	60	115
	65	182	1	1.1	0.46	0.7	1.75	4.6	60	120
	66	182	1	1.1	0.46	1.1	2.75	4.9	60	115
19	67	182	1	1.2	0.50	0.6	1.5	3.3	60	90
	68	182	1	1.7	0.70	0.6	1.5	3.9	60	80
	69	182	1	1.6	0.66	0.6	1.5	4.7	60	75
20	70	182	1	1.8	0.75	0.7	1.75	3.8	60	115
	71	182	1	1.3	0.54	0.6	1.5	4.9	60	115
	72	182	1	1.6	0.66	0.6	1.5	5.1	60	115
21	73	182	1	1.6	0.66	0.6	1.5	5.1	25	115
	74	182	1	5	2.07	0.6	1.5	8.5	25	115
	75	182	1	6.2	2.57	0.6	1.5	9.7	10	125
22	76	182	1	1.5	0.62	0.6	1.5	5.1	60	100
	77	182	1	7.4	3.07	0.6	1.5	11	60	95
	78	182	1	3.8	1.57	0.6	1.5	7.4	60	50
23	79	182	1	1.9	0.79	0.6	1.5	3.6	60	115
	80	182	1	1.8	0.75	0.6	1.5	4.4	60	110

	81	182	1	1.8	0.75	0.6	1.5	4.1	60	115
24	82	182	1	1.7	0.70	0.7	1.75	4.5	60	110
	83	182	1	1.7	0.70	0.7	1.75	4.7	60	110
	84	182	1	1.8	0.75	0.7	1.75	4.7	60	125
25	85	182	1	1.7	0.70	0.6	1.5	4.2	60	115
	86	182	1	1.6	0.66	0.6	1.5	4.9	60	150
	87	182	1	1.8	0.75	0.6	1.5	5.3	60	125
26	88	182	1	1.8	0.75	0.7	1.75	4.7	60	135
	89	182	1	2.2	0.91	0.7	1.75	4.8	60	130
	90	182	1	2.1	0.87	0.7	1.75	4.9	60	130
27	91	182	1	1.8	0.75	0.7	1.75	4.9	60	105
	92	182	1	1.9	0.79	0.7	1.75	5.5	60	105
	93	182	1	2	0.83	0.7	1.75	5.3	60	105
28	94	182	1	1.8	0.75	0.7	1.75	3.7	60	100
	95	182	1	1.7	0.70	0.7	1.75	4.3	60	150
	96	182	1	1.7	0.70	0.6	1.5	4	60	105
29	97	182	1	1.7	0.70	0.7	1.75	3.8	60	105
	98	182	1	1.7	0.70	0.7	1.75	4.4	60	105
	99	182	1	1.6	0.66	0.7	1.75	4.3	60	120
30	100	182	1	1.5	0.62	0.6	1.5	4.2	60	125
	101	182	1	1.1	0.46	0.7	1.75	3.6	60	120
	102	182	1	0.6	0.25	0.7	1.75	3.2	60	130
31	103	182	1	1.4	0.58	0.6	1.5	3.8	60	115
	104	182	1	1.4	0.58	0.6	1.5	3.8	15	120
	105	182	1	1.5	0.62	0.6	1.5	4	10	120
32	106	182	1	1.6	0.66	0.7	1.75	4.6	60	100
	107	182	1	1.6	0.66	0.5	1.25	4.5	60	100
	108	182	1	1.5	0.62	0.7	1.75	3.9	60	95
33	109	182	1	1.4	0.58	0.7	1.75	4.3	60	140
	110	182	1	1.1	0.46	0.6	1.5	4.3	60	140
	111	182	1	1.6	0.66	0.7	1.75	4.3	50	145
34	112	182	1	1.8	0.75	0.7	1.75	4.5	55	120
	113	182	1	1.9	0.79	0.7	1.75	4.7	40	120
	114	182	1	1.6	0.66	0.7	1.75	4.3	45	120
35	115	182	1	1.8	0.75	0.6	1.5	4	55	120
	116	182	1	1.5	0.62	0.6	1.5	3.7	50	130
	117	182	1	1.5	0.62	0.6	1.5	3.7	40	95
36	118	182	1	1.6	0.66	0.7	1.75	4.2	60	130
	119	182	1	1.5	0.62	0.7	1.75	4.1	60	125
	120	182	1	1.6	0.66	0.6	1.5	4	55	135

Synthesis Conditions of MOF-322

Table S2. Quantities of linker, metal, and modulator, total aqueous reaction volume, and respective reaction time and temperature utilized in microwave-assisted MOF-322 syntheses.

Iteration	Exp.	H_2TVDC		$AlCl_3$		NaOH		V_{total}	Time	Temperature
		[mg]	[mmol]	[mL]	[mmol]	[mL]	[mmol]	[mL]	[min]	[°C]
0	1	198	1	3.2	1.33	0.8	2	6.2	45	145
	2	198	1	7.2	2.98	1.4	3.5	11.2	30	125
	3	198	1	4.3	1.78	1	2.5	5.3	10	60
	4	198	1	5.7	2.36	1.1	2.75	6.9	35	115
	5	198	1	3.3	1.37	0.5	1.25	6.3	5	95
	6	198	1	7.5	3.11	1	2.5	9.2	20	130
	7	198	1	5.8	2.40	1.1	2.75	8.6	45	85
	8	198	1	3.5	1.45	1.2	3	7.5	50	135
	9	198	1	1.9	0.79	0.9	2.25	3.3	30	60
	10	198	1	0.8	0.33	1	2.5	4.8	20	140
	11	198	1	5.5	2.28	1.4	3.5	8.4	35	70
	12	198	1	1.7	0.70	0.7	1.75	3.7	5	90
1	13	198	1	2.6	1.08	0.7	1.75	4.1	45	150
	14	198	1	3	1.24	0.9	2.25	5.2	45	150
	15	198	1	3.2	1.33	0.5	1.25	5.4	45	145
2	16	198	1	1.3	0.54	0.7	1.75	2.9	40	150
	17	198	1	0.6	0.25	0.7	1.75	1.4	15	150
	18	198	1	1.8	0.75	0.7	1.75	2.7	30	150
3	19	198	1	0.6	0.25	1	2.5	2.3	40	150
	20	198	1	1.3	0.54	1.1	2.75	2.6	30	150
	21	198	1	1.3	0.54	1	2.5	2.5	20	150
4	22	198	1	1.1	0.46	0.8	2	3.6	40	145
	23	198	1	1.2	0.50	0.8	2	3.9	40	150
	24	198	1	0.5	0.21	0.8	2	4.2	40	150
5	25	198	1	1	0.41	0.8	2	3.5	40	100
	26	198	1	1.1	0.46	0.8	2	2.5	45	65
	27	198	1	0.9	0.37	0.8	2	1.9	60	65
6	28	198	1	1	0.41	0.9	2.25	3.9	35	145
	29	198	1	1.1	0.46	1.3	3.25	4.3	30	145
	30	198	1	0.8	0.33	1.3	3.25	4	5	145
7	31	198	1	1.2	0.50	0.8	2	4.3	40	150
	32	198	1	1.1	0.46	0.8	2	2.8	40	145
	33	198	1	1.2	0.50	0.8	2	2.5	40	150
8	34	198	1	1.1	0.46	0.7	1.75	3.6	5	145
	35	198	1	1.1	0.46	0.8	2	3.6	10	150
	36	198	1	1.1	0.46	0.7	1.75	3.7	35	145
9	37	198	1	1.1	0.46	1	2.5	4.2	40	145

	38	198	1	1.6	0.66	1.2	3	4.8	40	145
	39	198	1	3.3	1.37	1.4	3.5	6.8	40	145
10	40	198	1	6.9	2.86	0.8	2	9.6	40	150
	41	198	1	6.4	2.65	0.6	1.5	8.9	55	150
	42	198	1	7.4	3.07	0.6	1.5	10	40	150
11	43	198	1	1.1	0.46	0.8	2	3.9	40	145
	44	198	1	1.4	0.58	0.8	2	4.1	40	125
	45	198	1	1.2	0.50	0.8	2	3.7	55	135
12	46	198	1	1.2	0.50	0.8	2	3.7	55	150
	47	198	1	1.1	0.46	0.8	2	3.5	50	150
	48	198	1	1	0.41	0.6	1.5	3.2	60	150
13	49	198	1	1.1	0.46	0.6	1.5	3.3	40	145
	50	198	1	1.1	0.46	0.9	2.25	3.7	40	145
	51	198	1	1.6	0.66	0.9	2.25	4.1	40	145
14	52	198	1	0.7	0.29	0.8	2	3.3	40	150
	53	198	1	0.9	0.37	0.8	2	3.4	40	140
	54	198	1	0.7	0.29	0.7	1.75	3.3	45	150
15	55	198	1	1.6	0.66	0.8	2	4.3	30	150
	56	198	1	1.8	0.75	0.8	2	4.4	30	145
	57	198	1	1.1	0.46	0.8	2	3.7	35	150
16	58	198	1	1.2	0.50	0.6	1.5	3.7	45	150
	59	198	1	1.2	0.50	0.7	1.75	3.8	45	60
	60	198	1	1.1	0.46	0.6	1.5	3.6	50	50
17	61	198	1	1	0.41	0.8	2	3.7	50	150
	62	198	1	1	0.41	0.6	1.5	3.9	60	150
	63	198	1	0.8	0.33	0.8	2	3.8	60	145
18	64	198	1	1.2	0.50	0.8	2	3.9	40	145
	65	198	1	1.5	0.62	0.8	2	4	40	140
	66	198	1	3	1.24	0.8	2	5.5	40	140
19	67	198	1	0.5	0.21	0.8	2	3	40	145
	68	198	1	0.5	0.21	0.7	1.75	1.5	35	145
	69	198	1	0.5	0.21	0.5	1.25	2.7	30	145
20	70	198	1	1.5	0.62	0.5	1.25	3.7	40	150
	71	198	1	1.2	0.50	0.5	1.25	2.9	50	150
	72	198	1	1.7	0.70	0.5	1.25	2.4	55	150
21	73	198	1	1.7	0.70	0.8	2	4.5	40	150
	74	198	1	1.7	0.70	0.8	2	5.3	45	150
	75	198	1	3.5	1.45	0.8	2	7.2	55	150
22	76	198	1	1.2	0.50	0.8	2	3.9	50	150
	77	198	1	1.4	0.58	1.3	3.25	4.6	50	150
	78	198	1	1.4	0.58	1.3	3.25	5.3	50	150
23	79	198	1	1.9	0.79	0.7	1.75	4.5	40	150
	80	198	1	4.6	1.91	0.7	1.75	6.9	40	150

	81	198	1	5.1	2.11	0.7	1.75	7.1	35	150
24	82	198	1	1.2	0.50	0.8	2	3.4	40	150
	83	198	1	1	0.41	0.8	2	3.3	30	145
	84	198	1	1.6	0.66	0.8	2	3.8	5	145
25	85	198	1	1	0.41	0.7	1.75	3.7	40	150
	86	198	1	1	0.41	0.6	1.5	4.3	40	150
	87	198	1	1	0.41	0.7	1.75	4.7	40	145
26	88	198	1	3.7	1.53	0.8	2	6.4	40	150
	89	198	1	4	1.66	0.8	2	6.7	45	150
	90	198	1	4	1.66	0.8	2	6.5	40	150
27	91	198	1	1	0.41	0.8	2	2.7	40	115
	92	198	1	0.8	0.33	0.6	1.5	2.3	50	110
	93	198	1	0.8	0.33	0.5	1.25	2.3	60	120
28	94	198	1	1	0.41	0.7	1.75	3.4	60	140
	95	198	1	1.7	0.70	0.7	1.75	3.1	60	135
	96	198	1	6.5	2.69	0.7	1.75	8.9	60	140
29	97	198	1	1	0.41	0.8	2	3.9	40	150
	98	198	1	0.5	0.21	1.2	3	3.8	5	150
	99	198	1	0.5	0.21	0.9	2.25	3.7	5	150
30	100	198	1	1	0.41	0.8	2	1.8	55	145
	101	198	1	0.5	0.21	0.7	1.75	1.2	50	145
	102	198	1	1.2	0.50	0.8	2	2	45	115
31	103	198	1	1.1	0.46	0.8	2	3.4	60	140
	104	198	1	1.2	0.50	0.8	2	3.9	60	140
	105	198	1	3.1	1.28	0.8	2	4.4	60	140
32	106	198	1	2	0.83	0.8	2	4.5	50	150
	107	198	1	1.3	0.54	0.8	2	3.8	40	145
	108	198	1	2.2	0.91	0.8	2	4.5	45	145
33	109	198	1	2.4	0.99	0.8	2	3.5	50	150
	110	198	1	2.3	0.95	0.8	2	4.6	60	145
	111	198	1	2.4	0.99	0.8	2	3.6	45	145
34	112	198	1	2.1	0.87	0.8	2	4.4	35	145
	113	198	1	2.1	0.87	0.8	2	3.9	30	145
	114	198	1	2.2	0.91	0.6	1.5	4.3	15	150
35	115	198	1	2.2	0.91	0.8	2	4.4	50	140
	116	198	1	2.2	0.91	0.8	2	5.4	45	150
	117	198	1	2.2	0.91	0.8	2	5.8	50	55
36	118	198	1	2.2	0.91	0.7	1.75	4.4	50	150
	119	198	1	2.2	0.91	0.7	1.75	4.3	60	150
	120	198	1	2.2	0.91	0.7	1.75	4.7	60	150

Optimized Green Synthesis of MOF-321

In a 35 mL Pyrex pressure vessel, the linker H_2PZVDC (182 mg, 1 mmol) was completely dissolved in an aqueous NaOH solution (2.5 M, 0.7 mL, 1.75 eq.) This was followed by the addition of 2.2 mL deionized water. Subsequently, an aqueous $AlCl_3$ solution (0.41 M, 1.8 mL, 0.75 eq.) was incorporated into the solution, resulting in a white cloudy mixture. The mixture was heated to 125 °C in a microwave reactor for 60 minutes under vigorous stirring and a heating power of 300W. The resulting white solid was collected by centrifugation and washed with deionized water (3 × 15 mL) and ethanol (3 × 15 mL) over the course of a day, respectively. The MOF was then dried under dynamic vacuum conditions (~10^{-3} mbar) and heated to 120 °C over a period of 12 hours to yield the pure, activated product.

Optimized Green Synthesis of MOF-322

In a 35 mL Pyrex pressure vessel, the linker H_2TVDC (198 mg, 1 mmol) was completely dissolved in an aqueous NaOH solution (2.5 M, 0.8 mL, 2 eq.) This was followed by the addition of 3.5 mL deionized water. Subsequently, an aqueous $AlCl_3$ solution (0.41 M, 2.4 mL, 0.99 eq.) was incorporated into the solution, resulting in a white cloudy mixture. The mixture was heated to 150 °C in a microwave reactor for 50 minutes under vigorous stirring and a heating power of 300W. The resulting white solid was collected by centrifugation and washed with deionized water (3 × 15 mL) and ethanol (3 × 15 mL) over the course of a day, respectively. The MOF was then dried under dynamic vacuum conditions (~10^{-3} mbar) and heated to 120 °C over a period of 12 hours to yield the pure, activated product.

Elemental Analyses of MOF Samples

The washed and activated compounds were subjected to elemental analysis. The calculated formula for the elemental microanalysis results is given based on the input linker mole ratio:

Element. Anal. of activated MOF-321: Calcd. for $C_{56}H_{40}Al_8N_{16}O_{40}$: C, 37.52; H, 2.25; Al, 12.04; N, 12.50; O, 35.69 wt%; Found: C, 37.55; H, 2.35; N, 12.20 wt%.

Element. Anal. of activated MOF-322: Calcd. for $C_{40}H_{40}Al_8O_{40}S_8$: C, 40.01; H, 2.10; Al, 11.23; O, 33.31; S, 13.35 wt%; Found: C, 40.37; H, 2.10; S, 13.39 wt%.

Synthesis Conditions of COF-323

Table S3. Volumes of solvents, modulator and respective reaction time and temperature utilized in microwave-assisted COF-323 syntheses.

Exp.	Solvent 1	Volume	Solvent 2	Volume	Modulator	Volume	Temp.	Time
1	Dioxane	0.2 mL	TCB	0.8 mL	AcOH	0.16 mL	140 °C	2.5 h
2	Dioxane	0.1 mL	Mes	0.9 mL	AcOH	0.24 mL	110 °C	1.5 h
3	Dioxane	0.9 mL	DCB	0.1 mL	AcOH	0.32 mL	130 °C	2.5 h
4	Dioxane	0.8 mL	TCB	0.2 mL	AcOH	0.04 mL	130 °C	2 h
5	Dioxane	0.6 mL	Bn	0.4 mL	AcOH	0.08 mL	100 °C	2.5 h
6	Dioxane	0.8 mL	Tol	0.2 mL	AcOH	0.4 mL	130 °C	3 h
7	Dioxane	0.3 mL	Mes	0.7 mL	AcOH	0.16 mL	110 °C	1 h
8	Dioxane	0.4 mL	DCB	0.6 mL	AcOH	0.4 mL	120 °C	2 h
9	Dioxane	0.5 mL	Tol	0.5 mL	AcOH	0.16 mL	100 °C	1 h
10	Dioxane	0.1 mL	Mes	0.9 mL	AcOH	0.08 mL	120 °C	2.5 h
11	Dioxane	0.7 mL	Tol	0.3 mL	AcOH	0.4 mL	130 °C	3 h
12	Dioxane	0.8 mL	Tol	0.2 mL	AcOH	0.08 mL	140 °C	3 h
13	Dioxane	0.7 mL	Tol	0.3 mL	AcOH	0 mL	140 °C	3 h
14	Dioxane	0.8 mL	Mes	0.2 mL	AcOH	0.4 mL	100 °C	3 h
15	Dioxane	0.8 mL	Mes	0.2 mL	AcOH	0.2 mL	100 °C	3 h
16	Dioxane	0.8 mL	Mes	0.2 mL	AcOH	0.16 mL	100 °C	3 h
17	Dioxane	0.8 mL	Tol	0.2 mL	AcOH	0.4 mL	140 °C	3 h
18	Dioxane	0.7 mL	Tol	0.3 mL	AcOH	0.4 mL	140 °C	3 h
19	Dioxane	0.8 mL	TCB	0.2 mL	AcOH	0.4 mL	140 °C	3 h
20	Dioxane	1 mL	TCB	0 mL	AcOH	0.4 mL	140 °C	3 h
21	Dioxane	1 mL	Bn	0 mL	AcOH	0.4 mL	140 °C	3 h
22	Dioxane	1 mL	Bn	0 mL	AcOH	0.32 mL	140 °C	3 h
23	Dioxane	0.8 mL	TCB	0.2 mL	AcOH	0.4 mL	140 °C	2 h
24	Dioxane	0.9 mL	TCB	0.1 mL	AcOH	0.4 mL	140 °C	2 h
25	Dioxane	0.9 mL	TCB	0.1 mL	AcOH	0.4 mL	140 °C	2.5 h
26	Dioxane	0.8 mL	TCB	0.2 mL	AcOH	0.4 mL	130 °C	3 h
27	Dioxane	0.8 mL	DCB	0.2 mL	AcOH	0.4 mL	130 °C	3 h
28	Dioxane	0.8 mL	Bn	0.2 mL	AcOH	0.4 mL	130 °C	3 h

29	Dioxane	0.7 mL	TCB	0.3 mL	AcOH	0.4 mL	140 °C	3 h
30	Dioxane	0.7 mL	TCB	0.3 mL	AcOH	0.32 mL	140 °C	3 h
31	Dioxane	0.7 mL	TCB	0.3 mL	AcOH	0.24 mL	140 °C	3 h
32	Dioxane	0.8 mL	TCB	0.2 mL	AcOH	0.24 mL	140 °C	3 h
33	Dioxane	0.8 mL	TCB	0.2 mL	AcOH	0.28 mL	140 °C	3 h
34	Dioxane	0.8 mL	TCB	0.2 mL	AcOH	0.12 mL	140 °C	3 h
35	Dioxane	0.8 mL	Tol	0.2 mL	AcOH	0.36 mL	130 °C	3 h
36	Dioxane	1 mL	Tol	0 mL	AcOH	0.36 mL	130 °C	3 h
37	Dioxane	1 mL	Tol	0 mL	AcOH	0.32 mL	130 °C	3 h
38	Dioxane	0.8 mL	Bn	0.2 mL	AcOH	0.4 mL	140 °C	3 h
39	Dioxane	0.8 mL	Bn	0.2 mL	AcOH	0.36 mL	140 °C	3 h
40	Dioxane	0.8 mL	Bn	0.2 mL	AcOH	0.36 mL	140 °C	1.5 h
41	Dioxane	0.8 mL	Tol	0.2 mL	AcOH	0.4 mL	120 °C	3 h
42	Dioxane	0.8 mL	Mes	0.2 mL	AcOH	0.4 mL	120 °C	3 h
43	Dioxane	1 mL	Mes	0 mL	AcOH	0.4 mL	120 °C	3 h
44	Dioxane	0.8 mL	Mes	0.2 mL	AcOH	0.4 mL	130 °C	3 h
45	Dioxane	0.8 mL	Mes	0.2 mL	AcOH	0.4 mL	130 °C	1 h
46	Dioxane	0.8 mL	TCB	0.2 mL	AcOH	0.4 mL	140 °C	1 h
47	Dioxane	0.8 mL	Tol	0.2 mL	AcOH	0.4 mL	130 °C	2.5 h
48	Dioxane	0.8 mL	Tol	0.2 mL	AcOH	0.4 mL	130 °C	2 h
49	Dioxane	0.9 mL	Tol	0.1 mL	AcOH	0.4 mL	130 °C	2 h
50	Dioxane	0.6 mL	Tol	0.4 mL	AcOH	0.4 mL	130 °C	3 h
51	Dioxane	0.5 mL	Tol	0.5 mL	AcOH	0.4 mL	130 °C	3 h
52	Dioxane	0.6 mL	Tol	0.4 mL	AcOH	0.4 mL	110 °C	3 h
53	Dioxane	0.3 mL	TCB	0.7 mL	AcOH	0.4 mL	140 °C	3 h
54	Dioxane	0.2 mL	TCB	0.8 mL	AcOH	0.4 mL	140 °C	3 h
55	Dioxane	0.2 mL	Bn	0.8 mL	AcOH	0.4 mL	100 °C	3 h
56	Dioxane	0.1 mL	Tol	0.9 mL	AcOH	0.4 mL	140 °C	3 h
57	Dioxane	0.1 mL	Bn	0.9 mL	AcOH	0.4 mL	140 °C	3 h
58	Dioxane	0.1 mL	TCB	0.9 mL	AcOH	0.4 mL	140 °C	3 h
59	Dioxane	0.9 mL	Tol	0.1 mL	AcOH	0.4 mL	130 °C	3 h

60	Dioxane	0.9 mL	Mes	0.1 mL	AcOH	0.4 mL	130 °C	3 h
61	Dioxane	0.9 mL	Mes	0.1 mL	AcOH	0.4 mL	140 °C	3 h
62	Dioxane	0.5 mL	Mes	0.5 mL	AcOH	0.4 mL	140 °C	3 h
63	Dioxane	0.4 mL	Mes	0.6 mL	AcOH	0.4 mL	140 °C	2.5 h
64	Dioxane	0.3 mL	Mes	0.7 mL	AcOH	0.4 mL	140 °C	2 h
65	Dioxane	0.4 mL	TCB	0.6 mL	AcOH	0.4 mL	140 °C	3 h
66	Dioxane	0.4 mL	DCB	0.6 mL	AcOH	0.4 mL	140 °C	3 h
67	Dioxane	0.4 mL	Tol	0.6 mL	AcOH	0.4 mL	140 °C	3 h
68	Dioxane	0.4 mL	Tol	0.6 mL	AcOH	0.08 mL	140 °C	3 h
69	Dioxane	0.5 mL	Tol	0.5 mL	AcOH	0.08 mL	140 °C	3 h
70	Dioxane	0.4 mL	Tol	0.6 mL	AcOH	0 mL	140 °C	3 h
71	Dioxane	0.4 mL	Tol	0.6 mL	AcOH	0.4 mL	140 °C	2.5 h
72	Dioxane	0.3 mL	Tol	0.7 mL	AcOH	0.4 mL	140 °C	2.5 h
73	Dioxane	0.4 mL	Tol	0.6 mL	AcOH	0.4 mL	140 °C	2 h
74	Dioxane	0.8 mL	TCB	0.2 mL	AcOH	0.4 mL	140 °C	2.5 h
75	Dioxane	0.7 mL	TCB	0.3 mL	AcOH	0.4 mL	140 °C	2.5 h
76	Dioxane	0.7 mL	DCB	0.3 mL	AcOH	0.4 mL	140 °C	2.5 h
77	Dioxane	0.9 mL	TCB	0.1 mL	AcOH	0.4 mL	140 °C	3 h
78	Dioxane	0.9 mL	DCB	0.1 mL	AcOH	0.4 mL	140 °C	3 h
79	Dioxane	0.9 mL	Tol	0.1 mL	AcOH	0.4 mL	140 °C	3 h
80	Dioxane	0.8 mL	TCB	0.2 mL	AcOH	0.36 mL	140 °C	3 h
81	Dioxane	0.6 mL	TCB	0.4 mL	AcOH	0.36 mL	140 °C	3 h
82	Dioxane	0.7 mL	TCB	0.3 mL	AcOH	0.36 mL	140 °C	3 h

Elemental Analyses of COF Sample

Element. Anal. of activated COF-323 (exp 58): Calcd. for $C_{33}H_{21}N_9O_6$: C, 61.97; H, 3.31; N, 19.71 wt%. Found: C, 61.30; H, 3.38; N, 18.32 wt%.

Fourier-Transform Infrared Spectroscopy of COF Sample

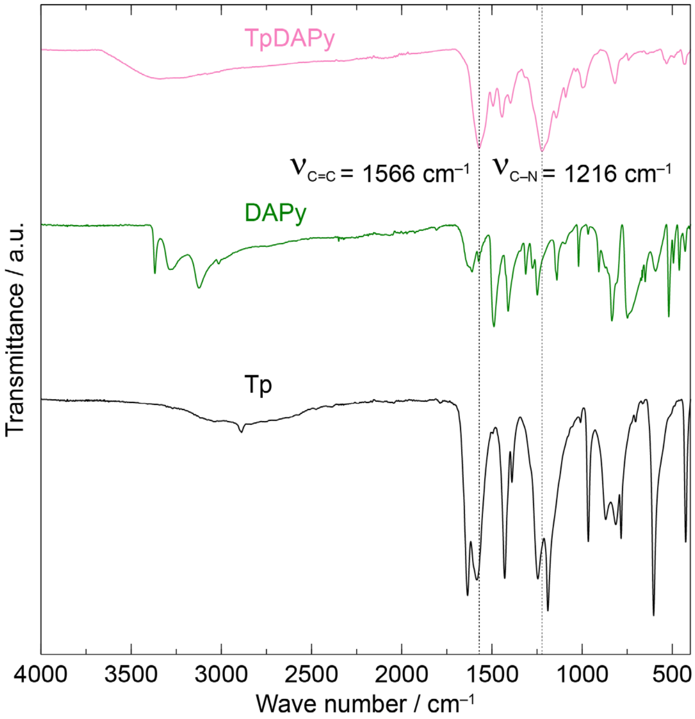

Figure S27. Overlay of the FT-IR spectra of Tp, DAPy, and TpDAPy COF. The new bands at 1566 cm^{-1} and 1216 cm^{-1} marked by the dashed line, are assigned to C=C and C–N bonds in β-ketoenamine linkages.

Section S5. Powder X-Ray Diffraction Analysis

<u>Sample Holder Preparation</u>

To ensure that the intensity and full width at half maximum (FWHM) values are not influenced by the quantity of the sample placed in the holder, a zero background holder with a central dimple was employed. This design ensures nearly identical quantities of sample for each measurement.

In the sample preparation process, a spatula is first used to position the sample over the hole at the center of the mirror surface. Following this, a clean glass slide is employed to press the sample into the holder. The application of downward pressure and circular motion ensures firm distribution of the powder in the holder. Once the sample is successfully leveled, any excess powder on the mirror surface, outside the dimple, is carefully removed using a clean kimwipe.

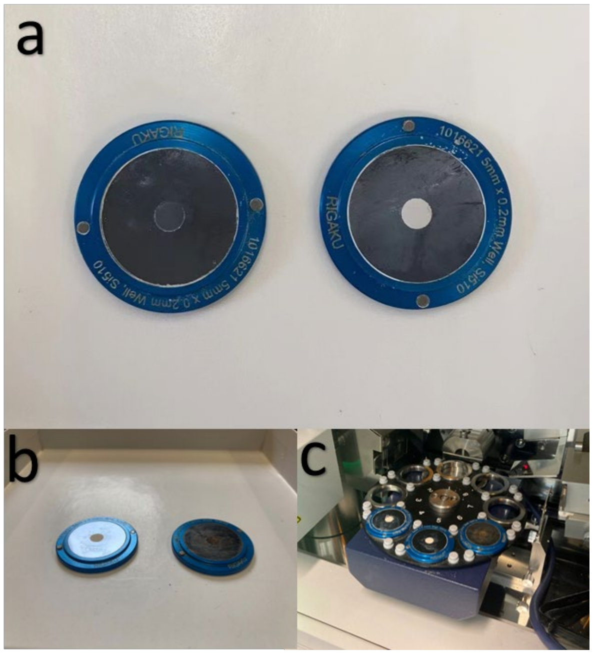

Figure S28. (a) Empty zero-background holder juxtaposed with the holder loaded with the MOF sample. (b) Dried and ground MOF sample (white, left) positioned on the holder and the dried and ground amorphous product (brown, right) contained within the holder. (c) Three samples from the same iteration individually loaded onto three distinct sample holders, subsequently placed in the RIGAKU PXRD instrument for sequential measurement. The COF samples were prepared and measured in a similar way.

Data Analysis

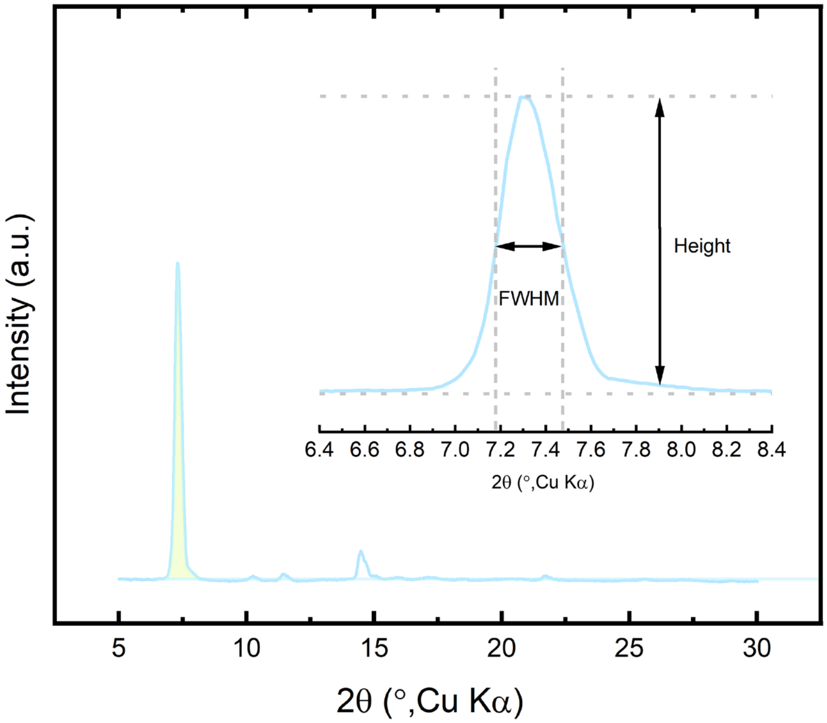

Figure S29. Illustration of primary peak of interest used to identify the formation of MOFs and the pivotal parameter for assessing crystallinity - FWHM and the peak height. The sharpness and narrowness of the peak, as indicated by a higher height and a lower FWHM, suggest enhanced crystallinity. The peak height is quantified as the intensity difference between the peak's apex and the baseline, while the FWHM is defined as the span between two points at half-maximum height on the y-axis.

PXRD Measurement on MOF-321 Samples

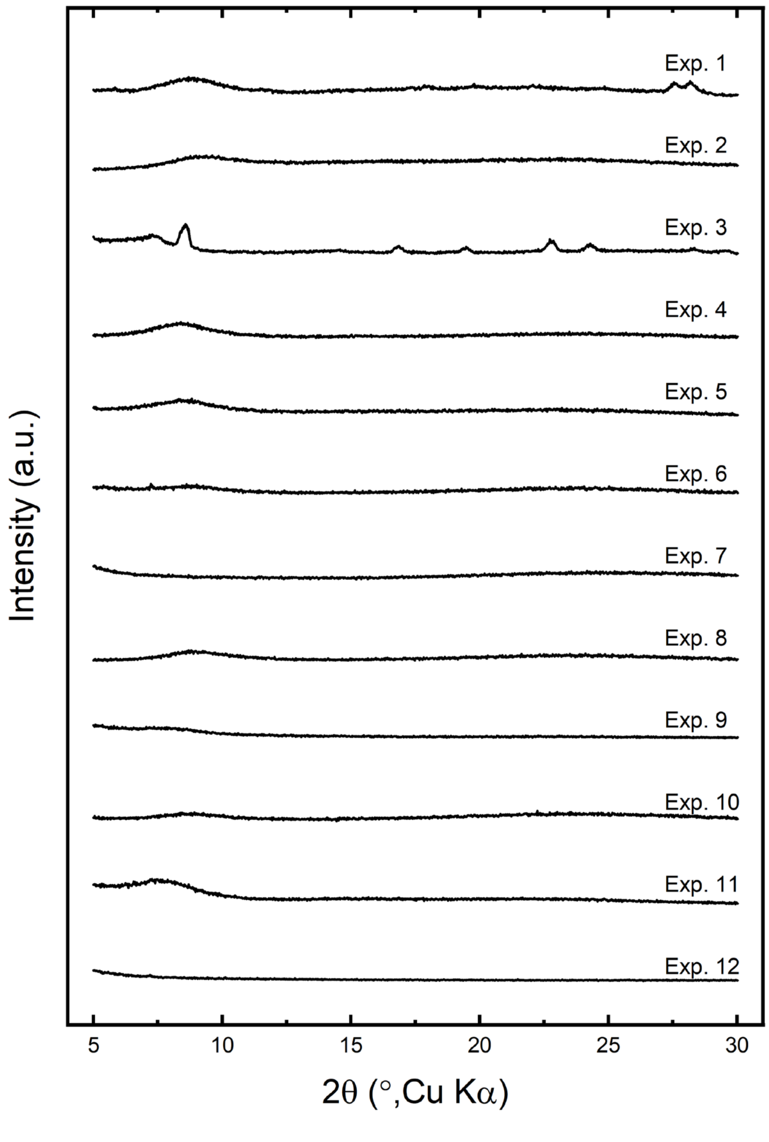

Figure S30. Powder X-ray diffraction analysis of MOF-321 compounds synthesized under reaction conditions described in Experiment 1 through Experiment 12 (Iteration 0) as outlined in Table S1. The conditions for the initial 12 experiments were selected randomly within a defined search space described in Section S3.

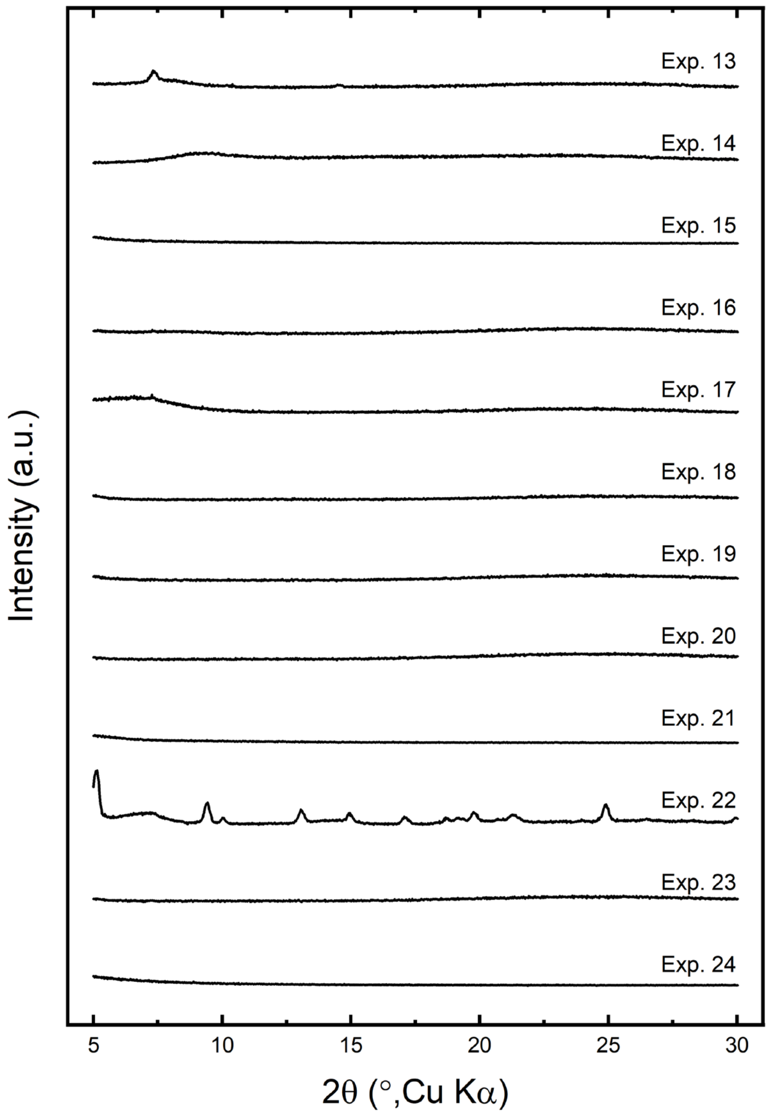

Figure S31. Powder X-ray diffraction analysis of MOF-321 compounds synthesized under reaction conditions specified in Experiments 13 through 24, corresponding to Iterations 1 through 4, as detailed in Table S1. These conditions were proposed by the BO algorithm, with each iteration comprising of three experiments.

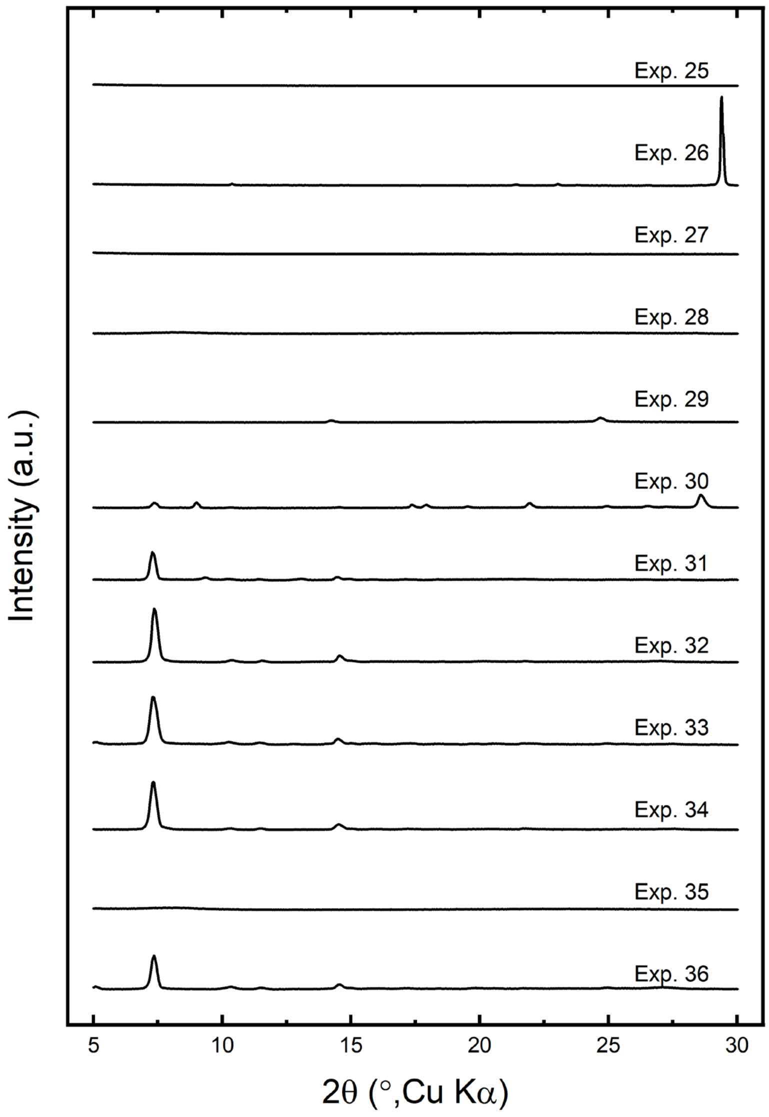

Figure S32. Powder X-ray diffraction analysis of MOF-321 compounds synthesized under reaction conditions specified in Experiments 25 through 36, corresponding to Iterations 5 through 8, as detailed in Table S1. These conditions were proposed by the BO algorithm, with each iteration comprising of three experiments.

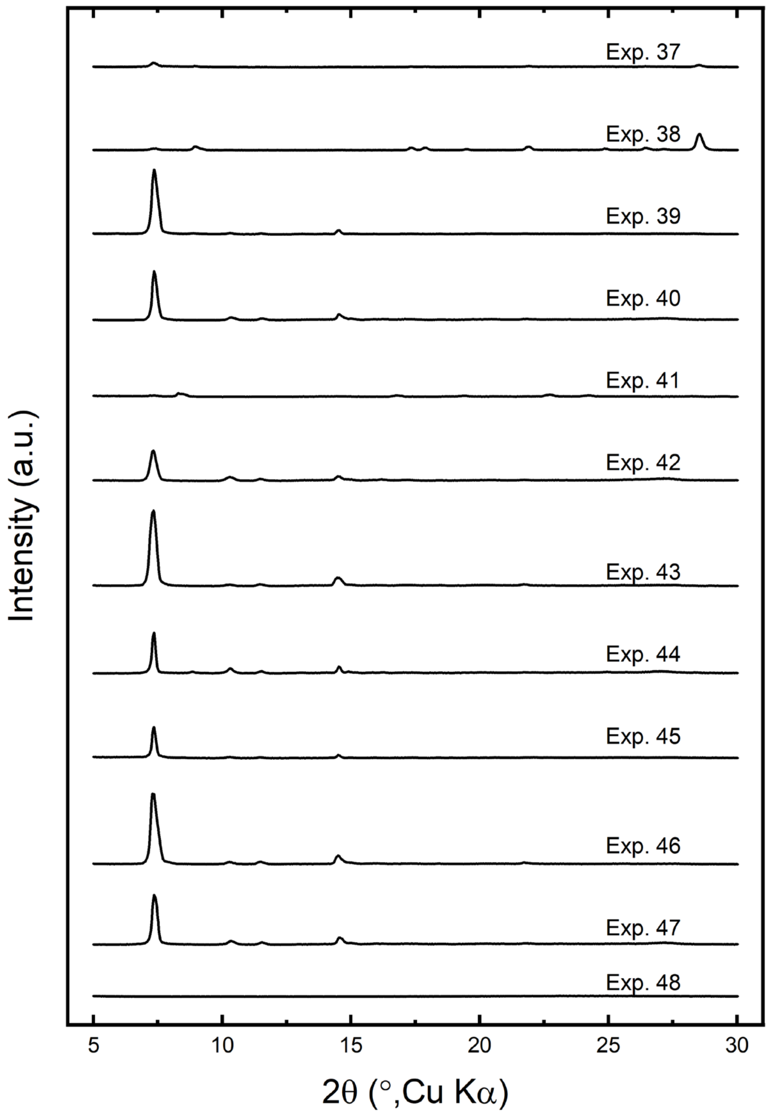

Figure S33. Powder X-ray diffraction analysis of MOF-321 compounds synthesized under reaction conditions specified in Experiments 37 through 48, corresponding to Iterations 9 through 12, as detailed in Table S1. These conditions were proposed by the BO algorithm, with each iteration comprising of three experiments.

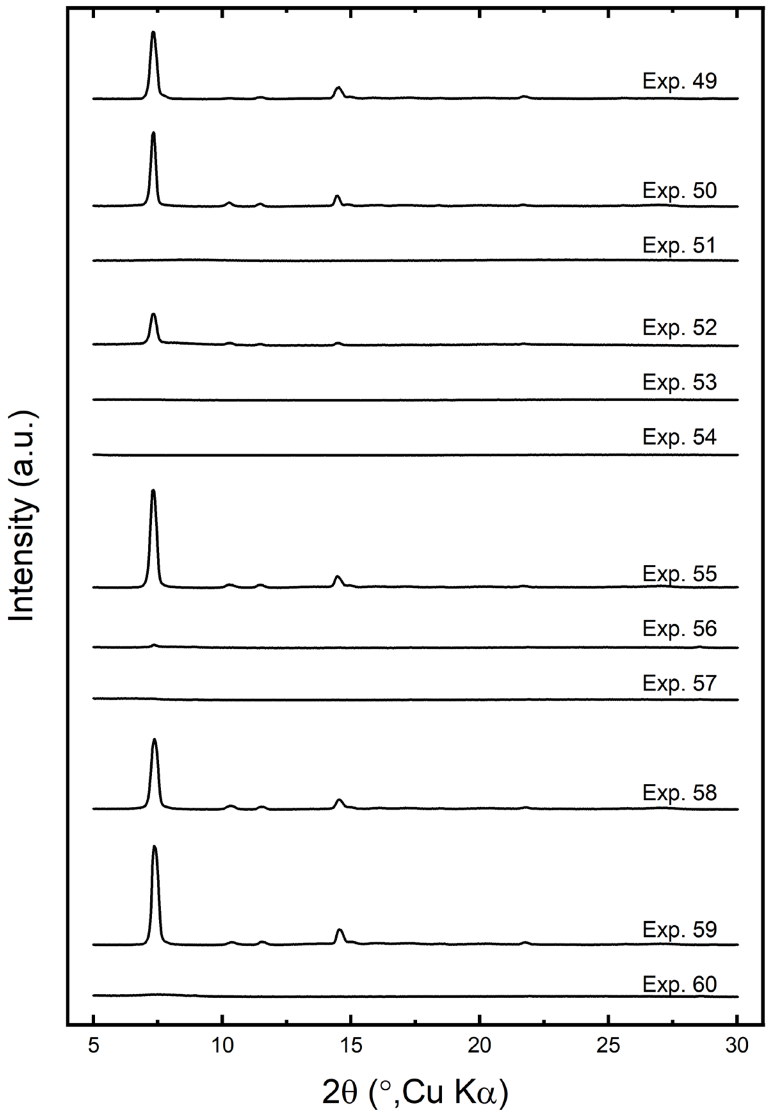

Figure S34. Powder X-ray diffraction analysis of MOF-321 compounds synthesized under reaction conditions specified in Experiments 49 through 60, corresponding to Iterations 13 through 16, as detailed in Table S1. These conditions were proposed by the BO algorithm, with each iteration comprising of three experiments.

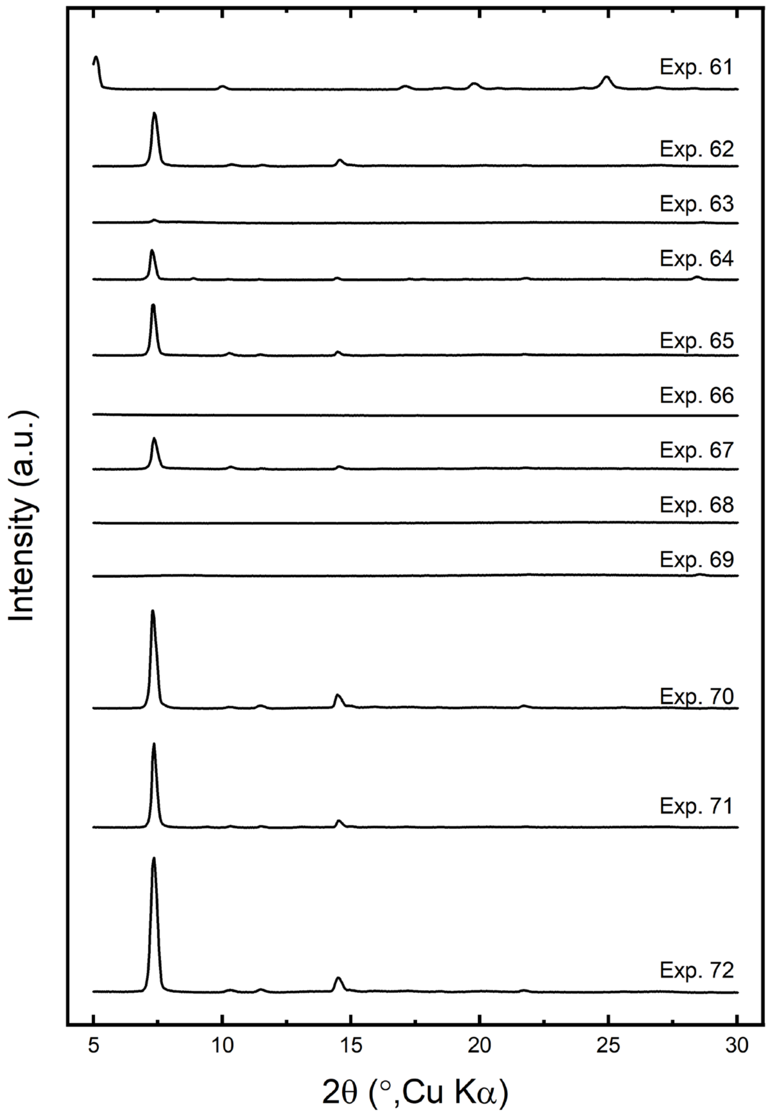

Figure S35. Powder X-ray diffraction analysis of MOF-321 compounds synthesized under reaction conditions specified in Experiments 61 through 72, corresponding to Iterations 17 through 20, as detailed in Table S1. These conditions were proposed by the BO algorithm, with each iteration comprising of three experiments.

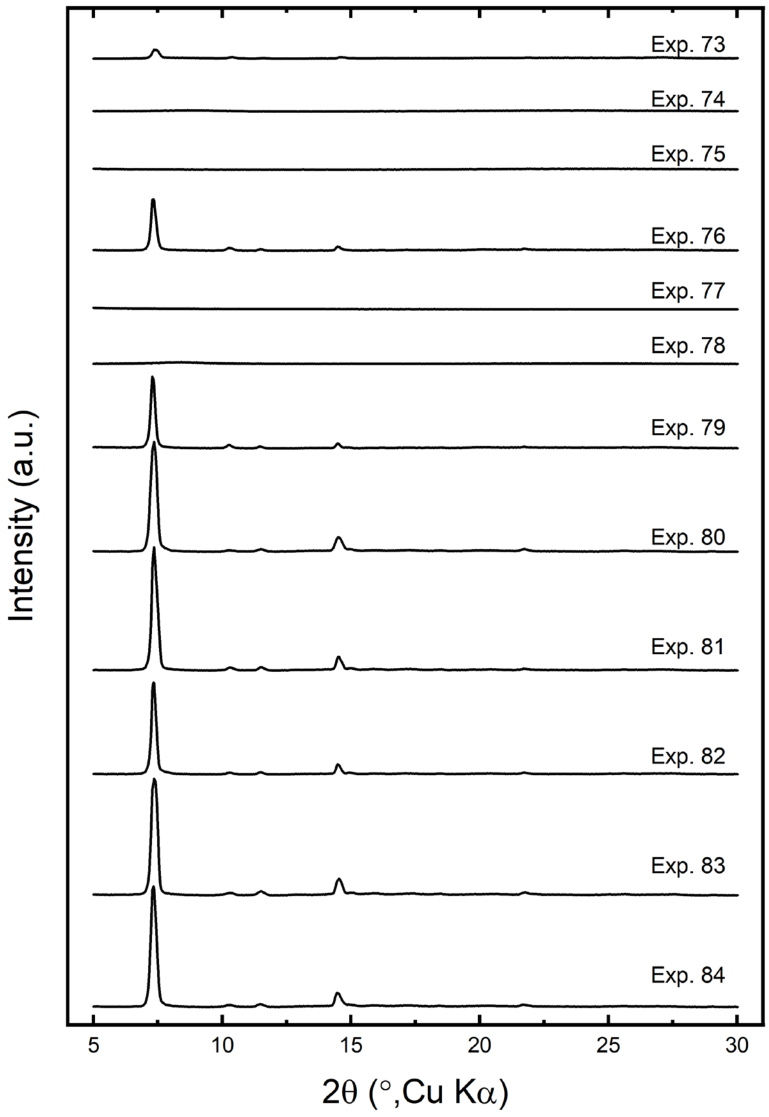

Figure S36. Powder X-ray diffraction analysis of MOF-321 compounds synthesized under reaction conditions specified in Experiments 73 through 84, corresponding to Iterations 21 through 24, as detailed in Table S1. These conditions were proposed by the BO algorithm, with each iteration comprising of three experiments.

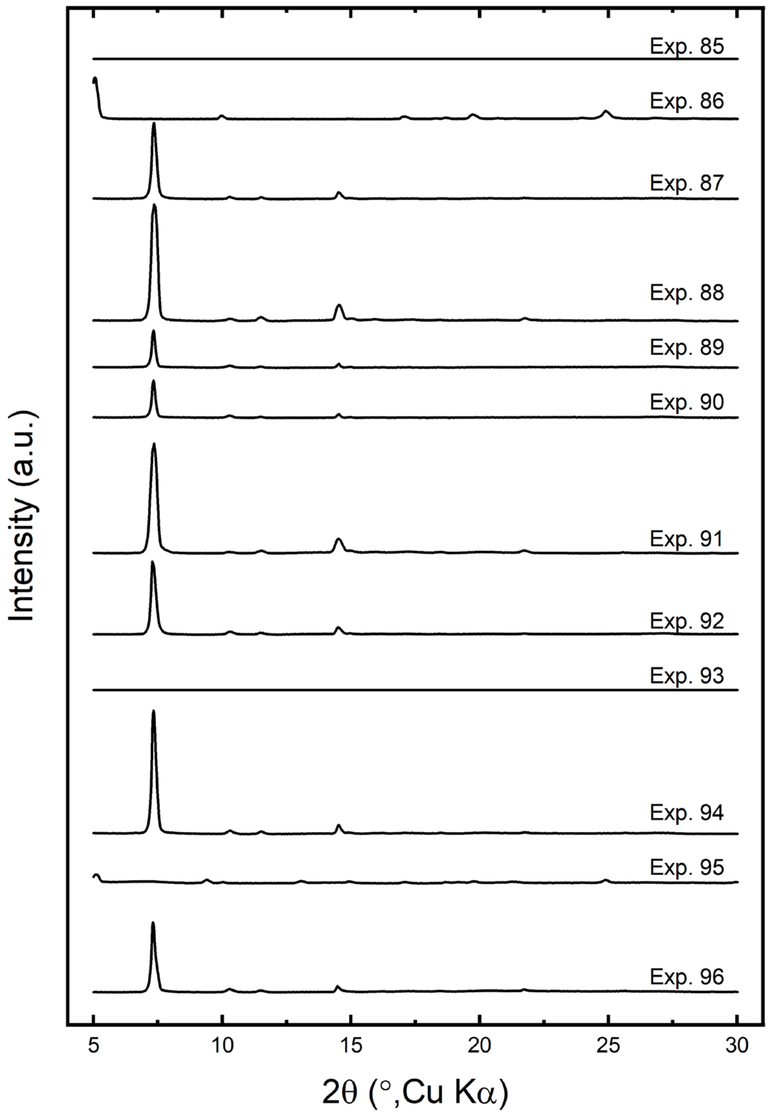

Figure S37. Powder X-ray diffraction analysis of MOF-321 compounds synthesized under reaction conditions specified in Experiments 85 through 96, corresponding to Iterations 25 through 28, as detailed in Table S1. These conditions were proposed by the BO algorithm, with each iteration comprising of three experiments.

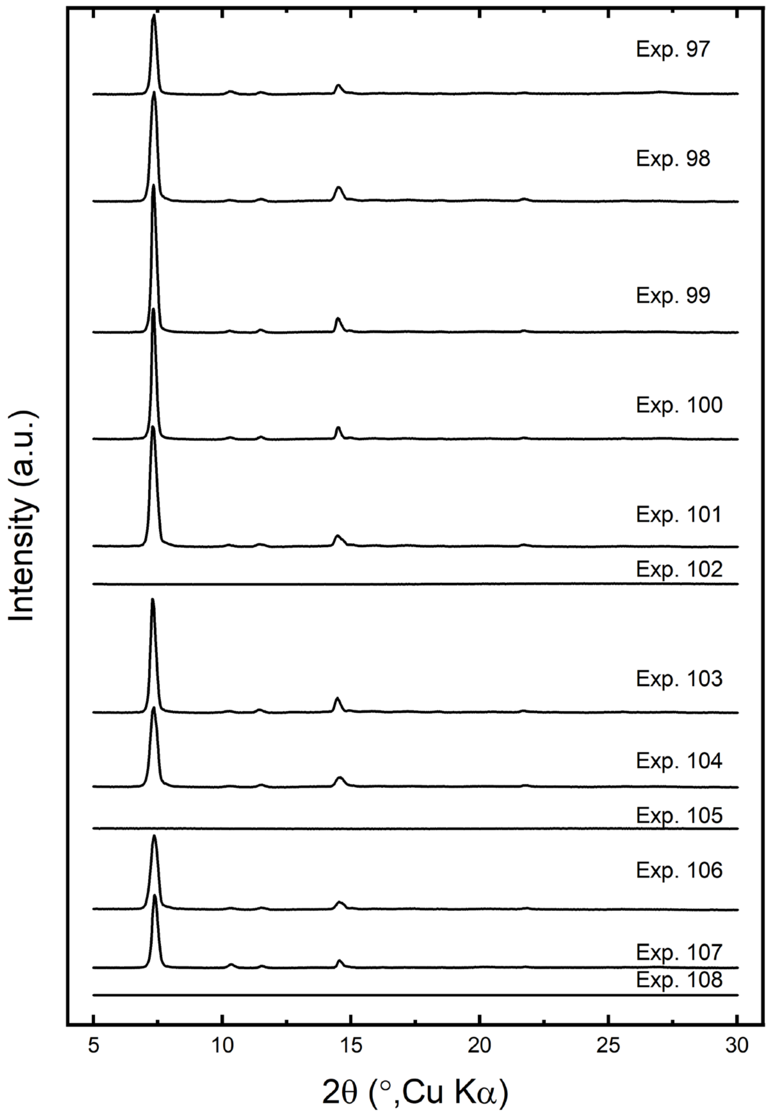

Figure S38. Powder X-ray diffraction analysis of MOF-321 compounds synthesized under reaction conditions specified in Experiments 97 through 108, corresponding to Iterations 29 through 32, as detailed in Table S1. These conditions were proposed by the BO algorithm, with each iteration comprising of three experiments.

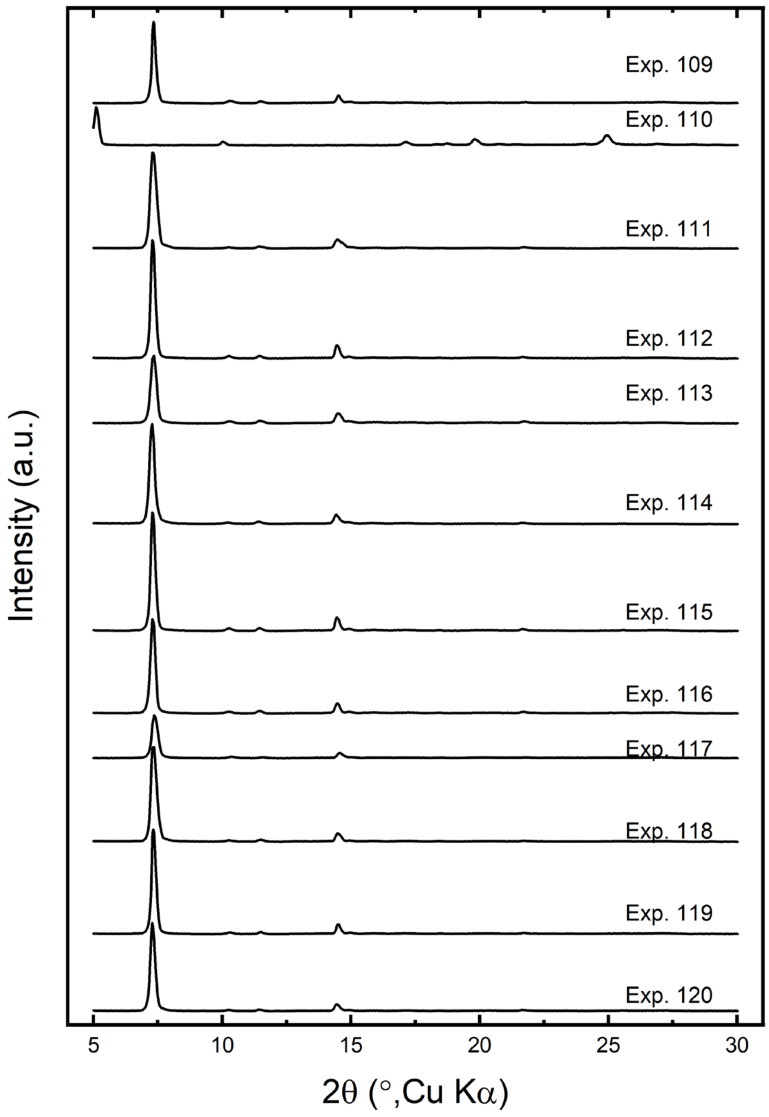

Figure S39. Powder X-ray diffraction analysis of MOF-321 compounds synthesized under reaction conditions specified in Experiments 109 through 120, corresponding to Iterations 33 through 36, as detailed in Table S1. These conditions were proposed by the BO algorithm, with each iteration comprising of three experiments.

PXRD Measurement on MOF-322 Samples

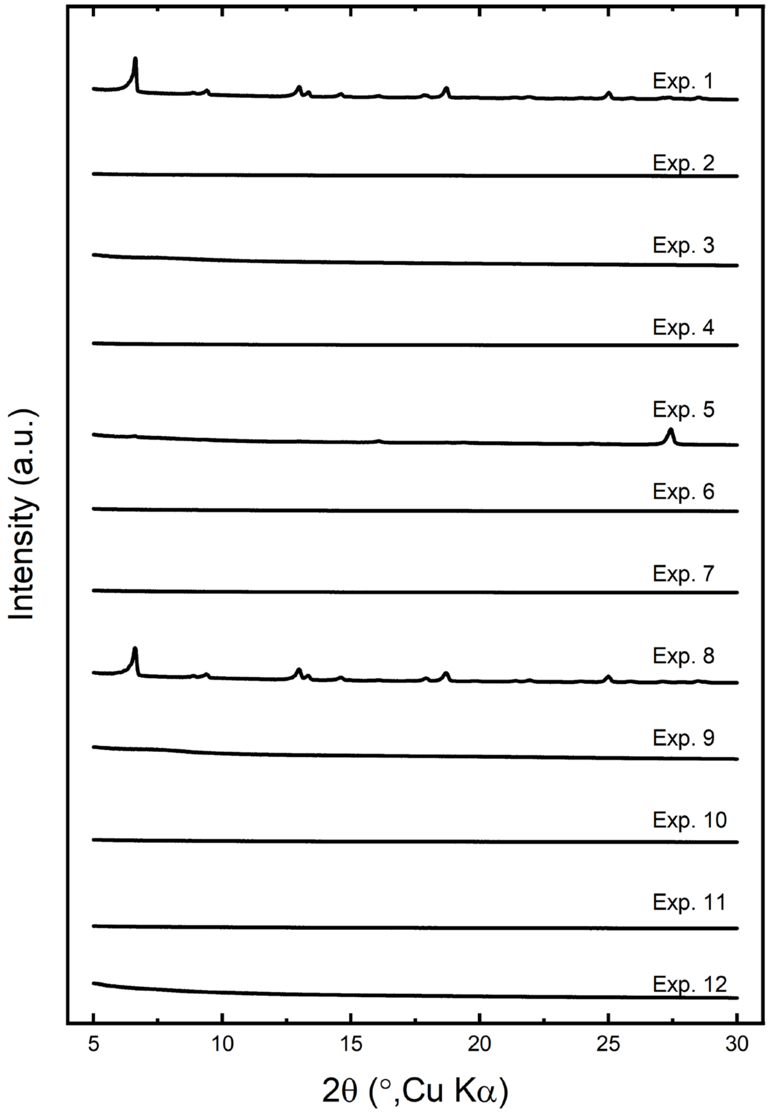

Figure S40. Powder X-ray diffraction analysis of MOF-322 compounds synthesized under synthesized under reaction conditions described in Experiment 1 through Experiment 12 as outlined in Table S2. The conditions for the initial 12 experiments were selected randomly within a defined search space described in Section S3.

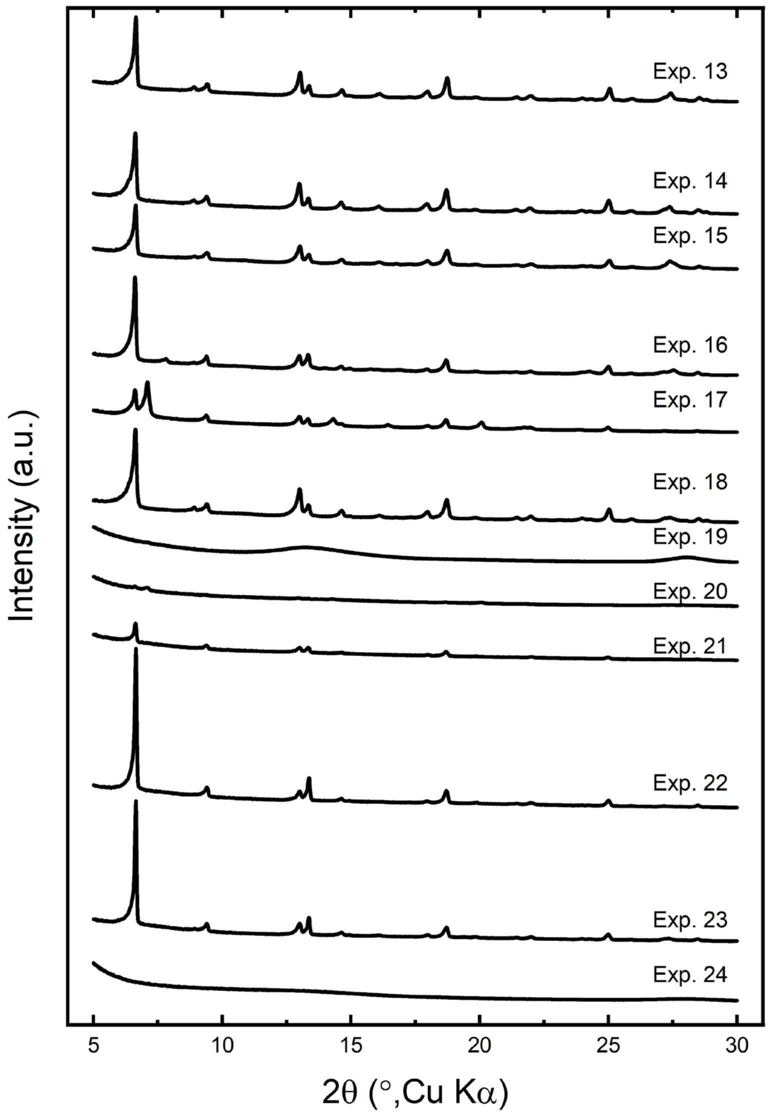

Figure S41. Powder X-ray diffraction analysis of MOF-322 compounds synthesized under reaction conditions specified in Experiments 13 through 24, corresponding to Iterations 1 through 4, as detailed in Table S2. These conditions were proposed by the BO algorithm, with each iteration comprising of three experiments.

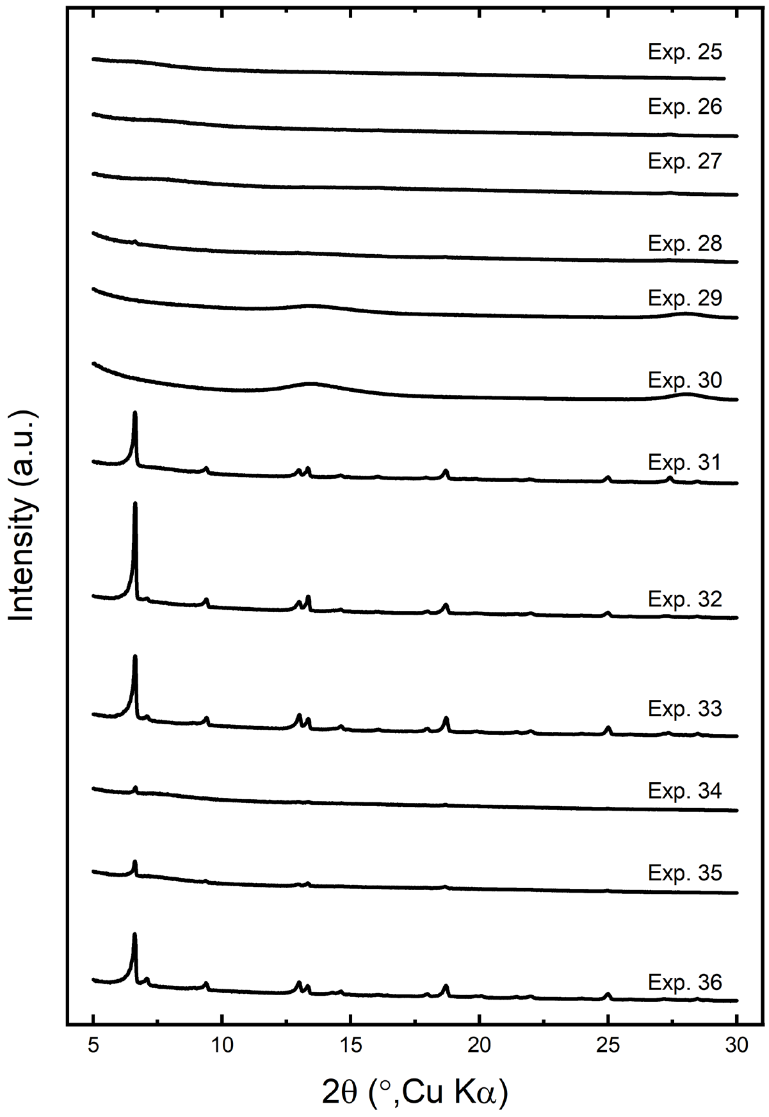

Figure S42. Powder X-ray diffraction analysis of MOF-322 compounds synthesized under reaction conditions specified in Experiments 25 through 36, corresponding to Iterations 5 through 8, as detailed in Table S2. These conditions were proposed by the BO algorithm, with each iteration comprising of three experiments.

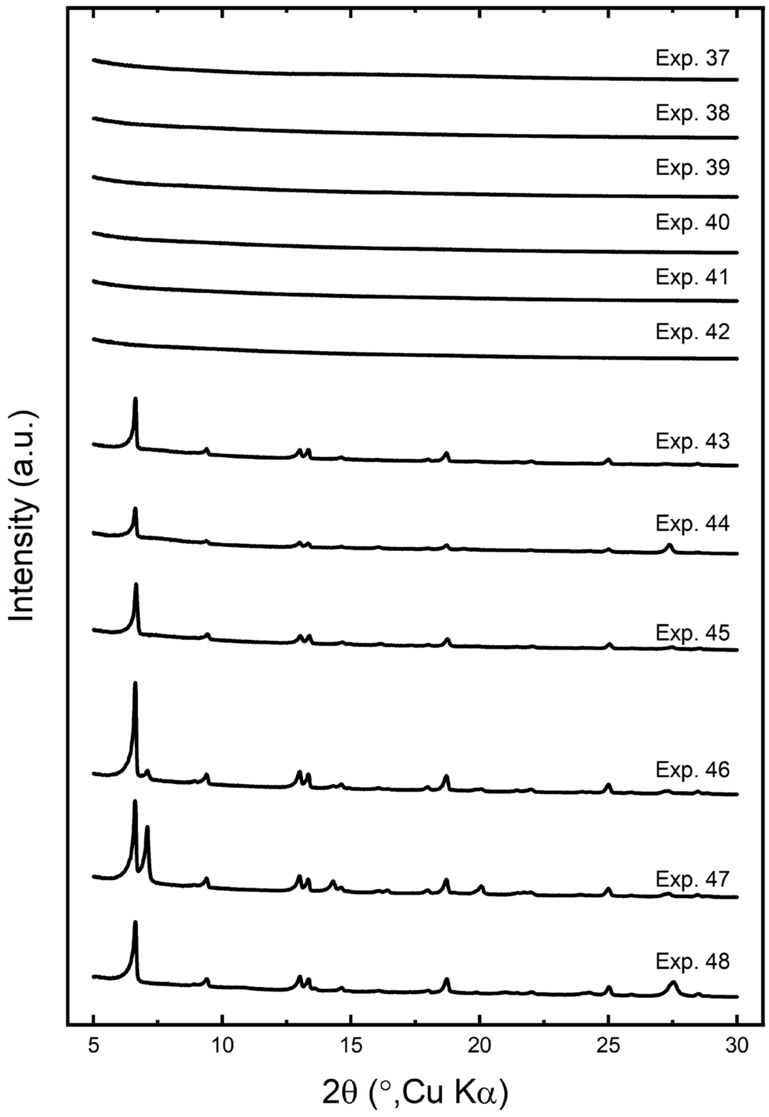

Figure S43. Powder X-ray diffraction analysis of MOF-322 compounds synthesized under reaction conditions specified in Experiments 37 through 48, corresponding to Iterations 9 through 12, as detailed in Table S2. These conditions were proposed by the BO algorithm, with each iteration comprising of three experiments.

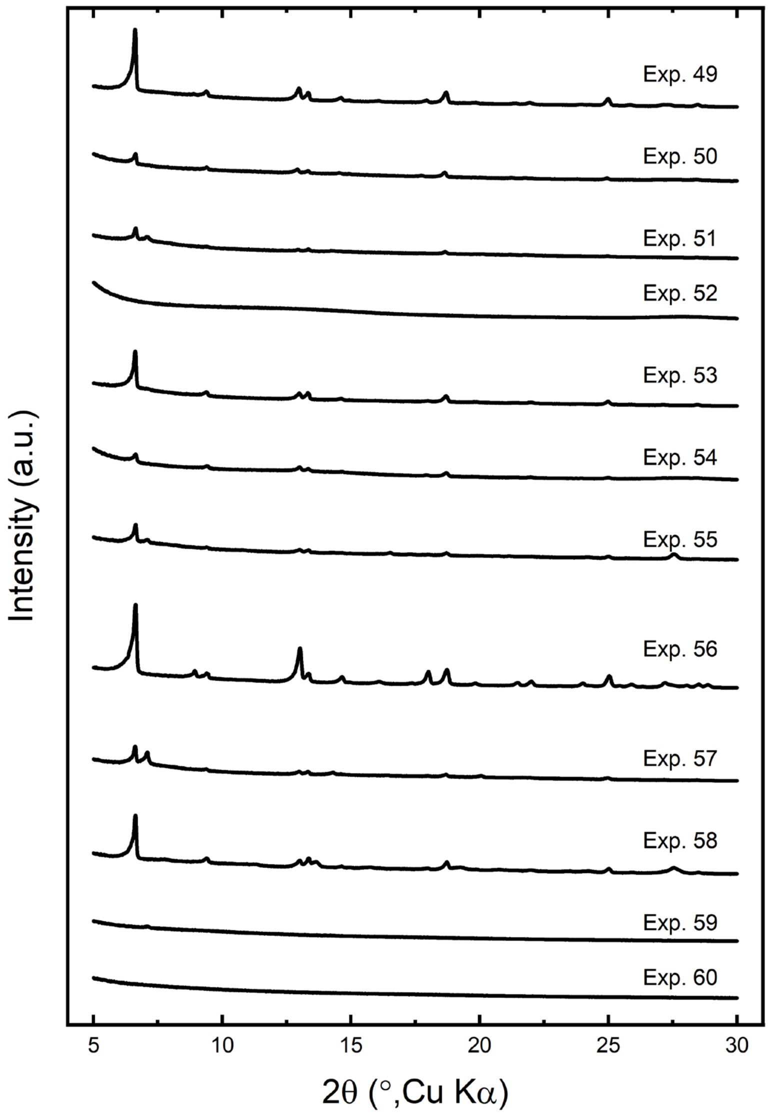

Figure S44. Powder X-ray diffraction analysis of MOF-322 compounds synthesized under reaction conditions specified in Experiments 49 through 60, corresponding to Iterations 13 through 16, as detailed in Table S2. These conditions were proposed by the BO algorithm, with each iteration comprising of three experiments.

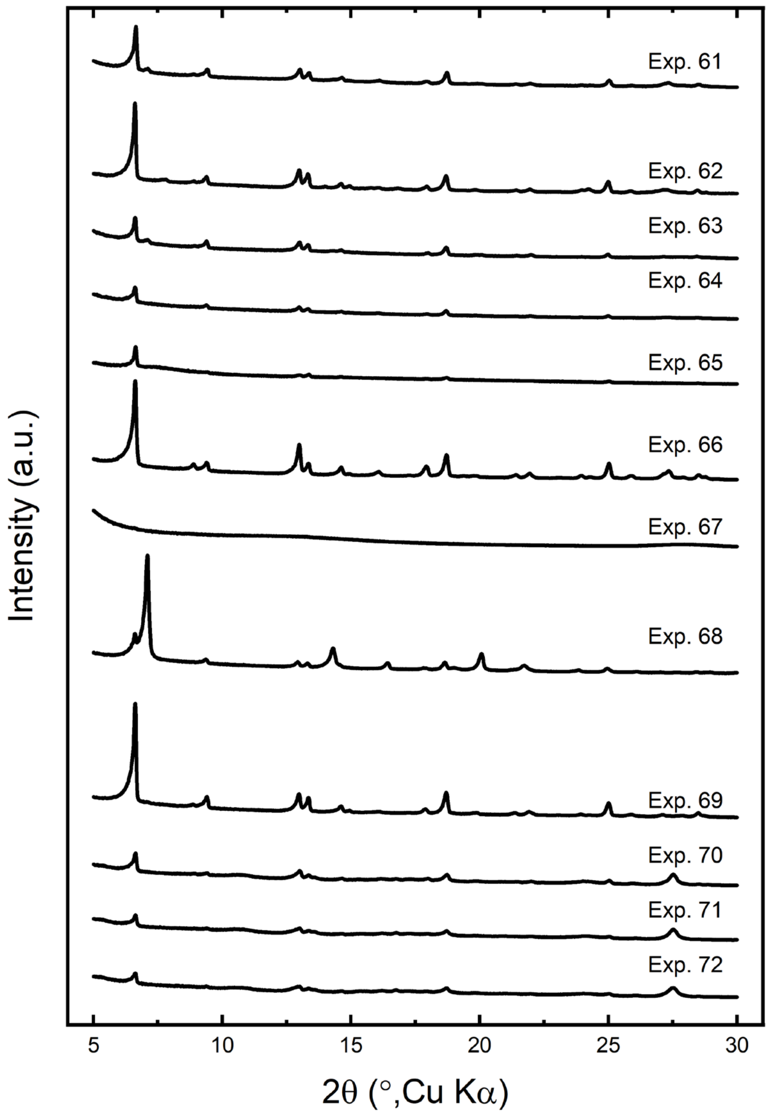

Figure S45. Powder X-ray diffraction analysis of MOF-322 compounds synthesized under reaction conditions specified in Experiments 61 through 72, corresponding to Iterations 17 through 20, as detailed in Table S2. These conditions were proposed by the BO algorithm, with each iteration comprising of three experiments.

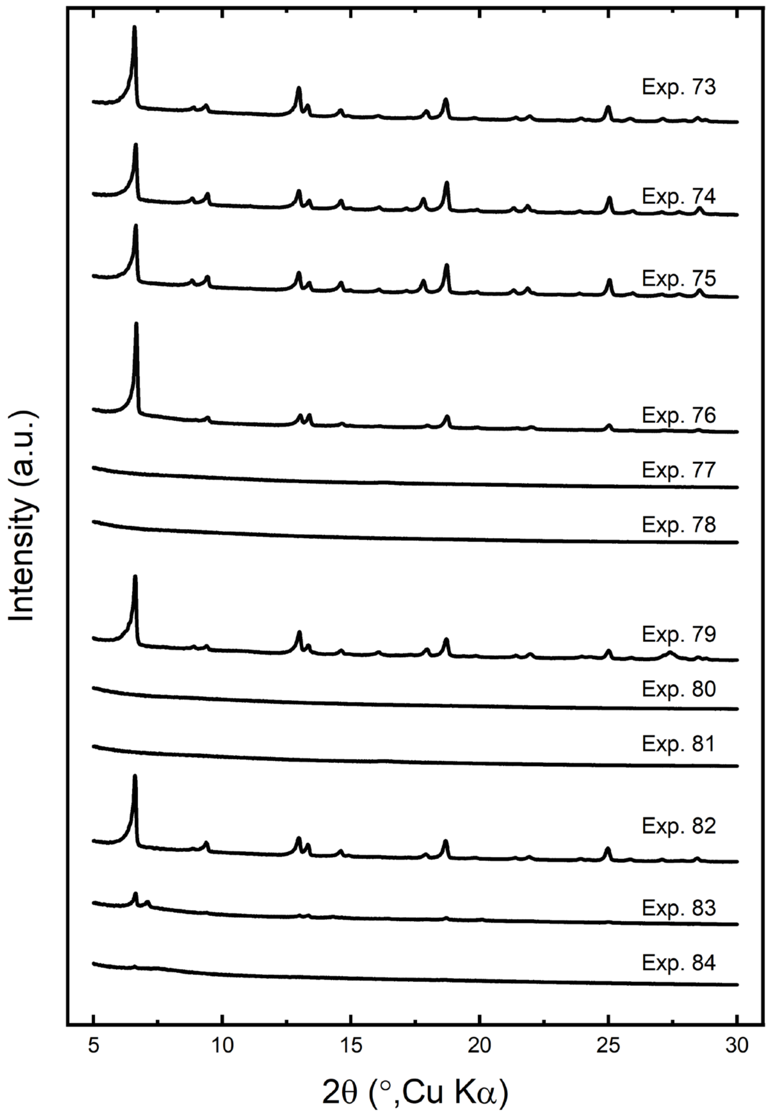

Figure S46. Powder X-ray diffraction analysis of MOF-322 compounds synthesized under reaction conditions specified in Experiments 73 through 84, corresponding to Iterations 21 through 24, as detailed in Table S2. These conditions were proposed by the BO algorithm, with each iteration comprising of three experiments.

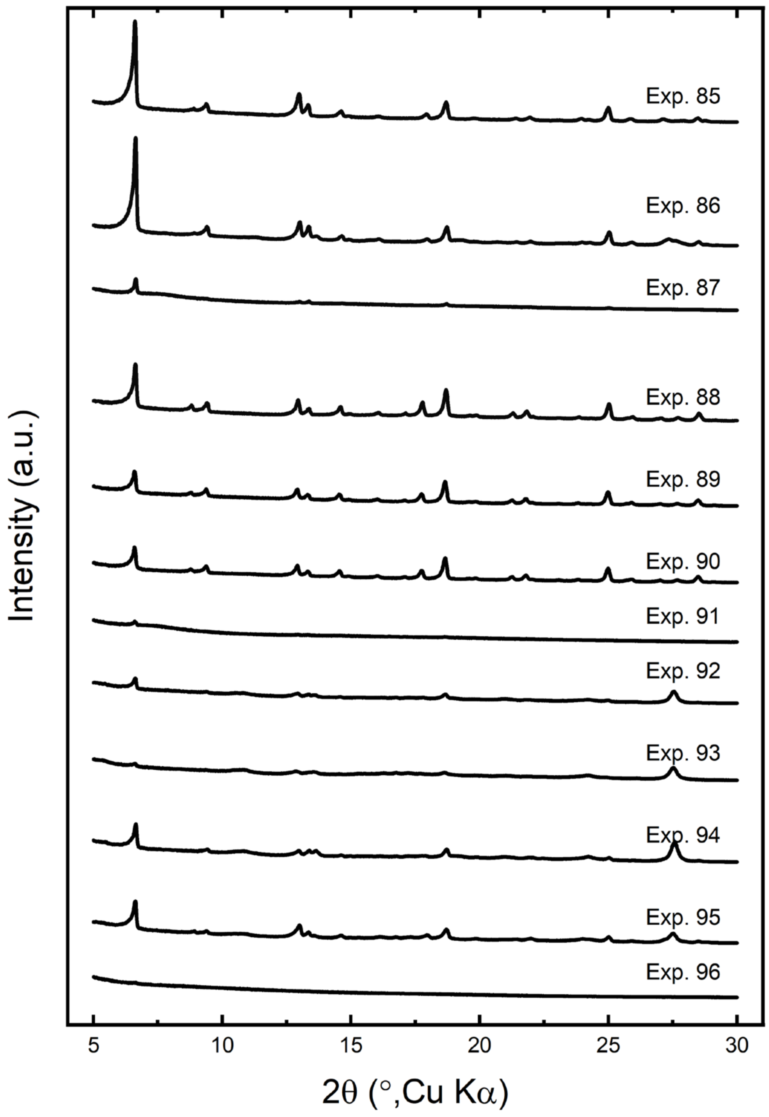

Figure S47. Powder X-ray diffraction analysis of MOF-322 compounds synthesized under reaction conditions specified in Experiments 85 through 96, corresponding to Iterations 25 through 28, as detailed in Table S2. These conditions were proposed by the BO algorithm, with each iteration comprising of three experiments.

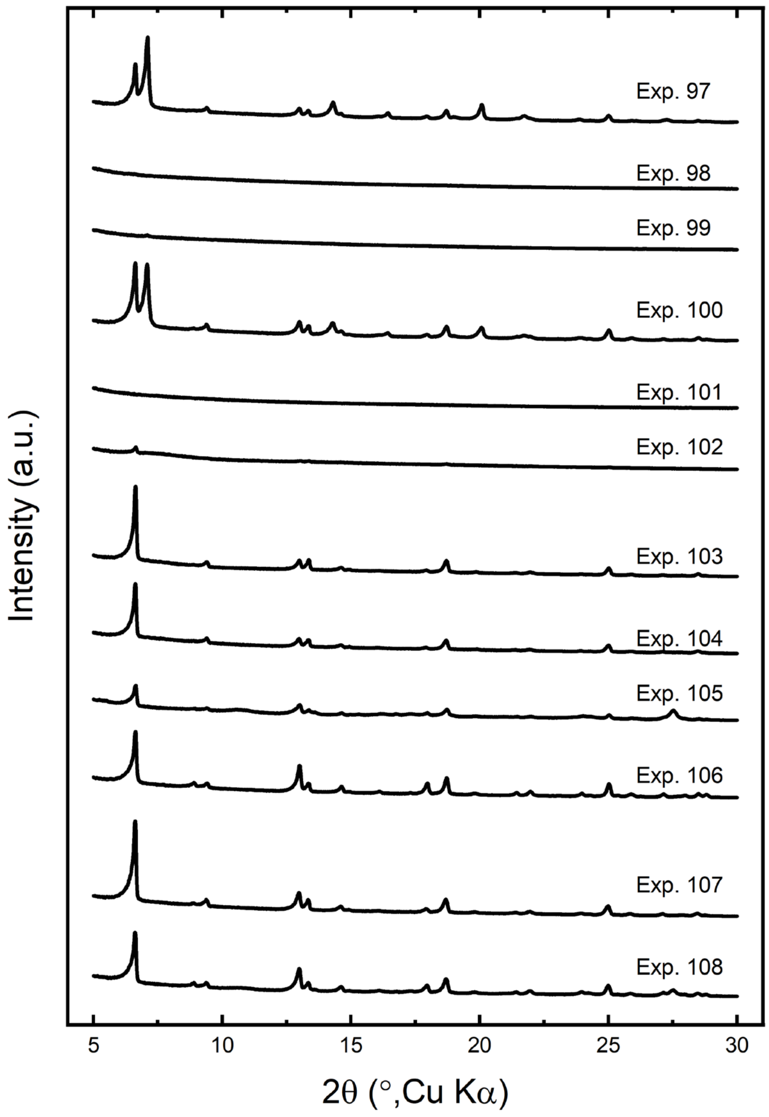

Figure S48. Powder X-ray diffraction analysis of MOF-322 compounds synthesized under reaction conditions specified in Experiments 97 through 108, corresponding to Iterations 29 through 32, as detailed in Table S2. These conditions were proposed by the BO algorithm, with each iteration comprising of three experiments.

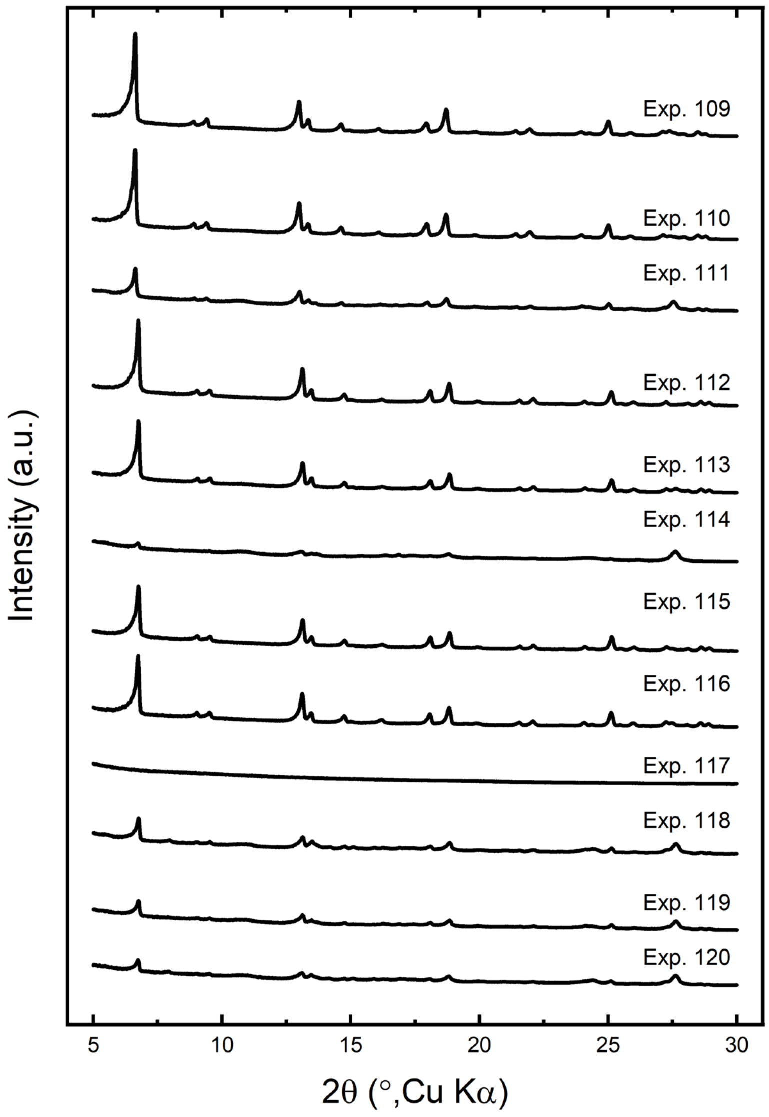

Figure S49. Powder X-ray diffraction analysis of MOF-322 compounds synthesized under reaction conditions specified in Experiments 109 through 120, corresponding to Iterations 33 through 36, as detailed in Table S2. These conditions were proposed by the BO algorithm, with each iteration comprising of three experiments.

Structure Modelling and Elucidation of MOF-322

MOF-322 was modeled in the space group *I4₁/a* (No. 14). Using the BIOVIA Materials Studio 2020 software, Pawley refinement was conducted against the experimental powder X-ray diffraction data of MOF-322 to provide the unit cell parameters ($a = b = 26.3$ Å, $c = 10.5$ Å, $\alpha = \beta = \gamma = 90°$) with good agreement factors ($R_p = 6.22\%$, $R_{wp} = 9.19\%$).

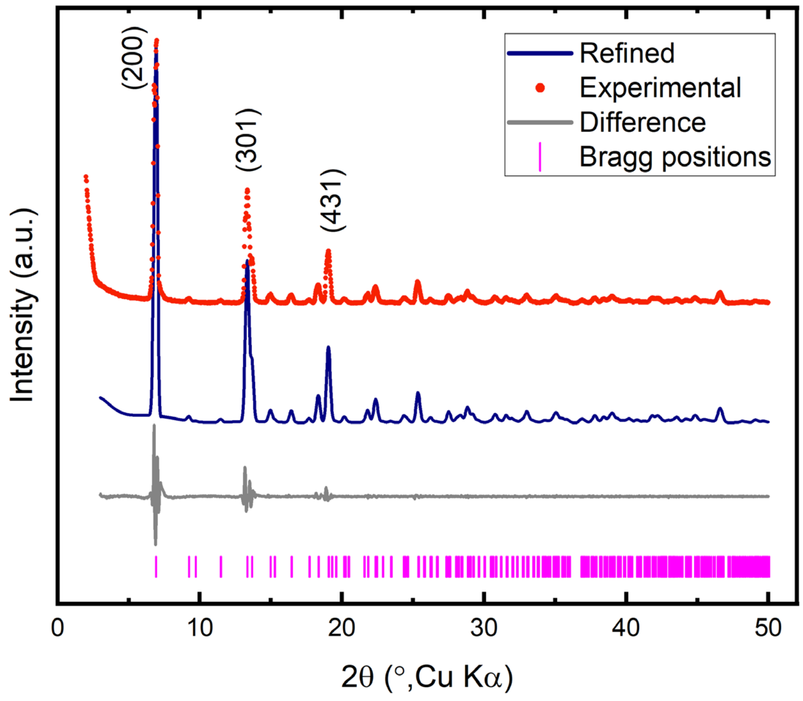

Figure S50. Pawley refinement of MOF-322 in the *cis*-connected aluminum seconding building unit conformation against the experimental PXRD pattern of MOF-322. Major peaks are indexed.

Table S4. Crystallographic information file of refined MOF-322 structure.

```
_symmetry_space_group_name_H-M    'I41/A'
_symmetry_Int_Tables_number       88
_symmetry_cell_setting            tetragonal
loop_
_symmetry_equiv_pos_as_xyz
  x,y,z
  -x+1/2,-y,z+1/2
  -y+3/4,x+1/4,z+1/4
  y+3/4,-x+3/4,z+3/4
  -x,-y,-z
  x+1/2,y,-z+1/2
  y+1/4,-x+3/4,-z+3/4
  -y+1/4,x+1/4,-z+1/4
  x+1/2,y+1/2,z+1/2
  -x,-y+1/2,z
  -y+1/4,x+3/4,z+3/4
  y+1/4,-x+1/4,z+1/4
  -x+1/2,-y+1/2,-z+1/2
  x,y+1/2,-z
  y+3/4,-x+1/4,-z+1/4
  -y+3/4,x+3/4,-z+3/4
_cell_length_a                    26.2764
_cell_length_b                    26.2764
_cell_length_c                    10.5251
_cell_angle_alpha                 90.0000
_cell_angle_beta                  90.0000
_cell_angle_gamma                 90.0000
loop_
_atom_site_label
_atom_site_type_symbol
_atom_site_fract_x
_atom_site_fract_y
_atom_site_fract_z
Al1    Al    0.19690   0.50320   0.04680
O2     O     0.24730   0.50510  -0.07410
O3     O     0.19890   0.57690   0.06650
O4     O     0.14310   0.50600  -0.06940
O5     O     0.19070   0.43440   0.02590
O6     O     0.14320   0.49800   0.16170
C7     C     0.22310   0.61360   0.01900
C8     C     0.20870   0.89330  -0.07300
C9     C     0.21370   0.66620   0.07200
H10    H     0.19631   0.67093   0.17124
C11    C     0.22330   0.79290  -0.04380
C12    C     0.20990   0.84140   0.00690
C13    C     0.19370   0.83430   0.13530
S14    S     0.19710   0.78140   0.16390
C15    C     0.21540   0.75580   0.05330
C16    C     0.22640   0.70460  -0.00100
H17    H     0.24450   0.69734  -0.09847
H18    H     0.23748   0.78960  -0.14655
H19    H     0.18107   0.86850   0.19484
```

PXRD Measurement on COF-323 Samples

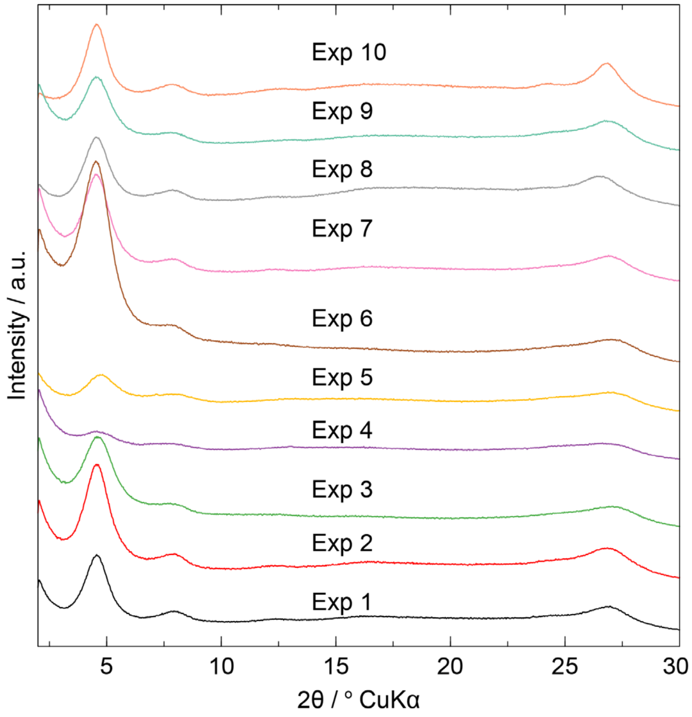

Figure S51. Powder X-ray diffraction analysis of COF-323 compounds synthesized under reaction conditions specified in Experiments 1 through 10.

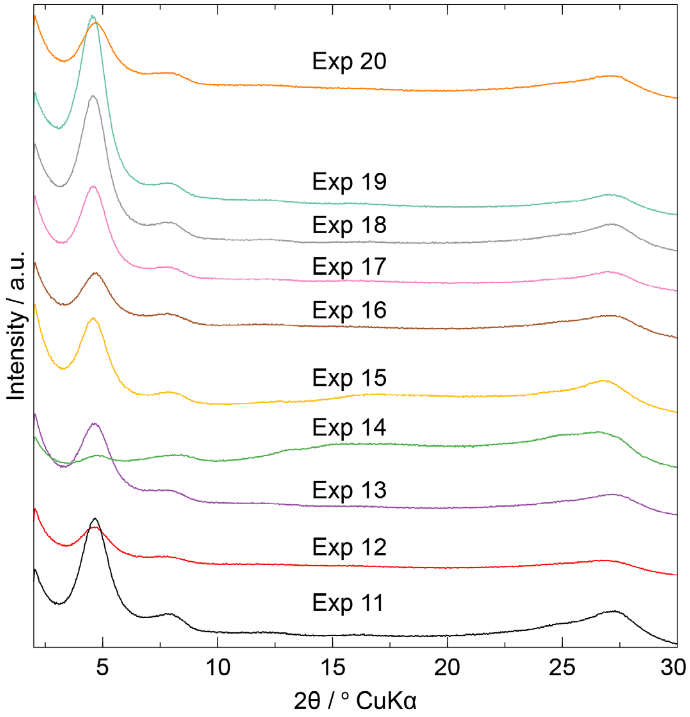

Figure S52. Powder X-ray diffraction analysis of COF-323 compounds synthesized under reaction conditions specified in Experiments 11 through 20.

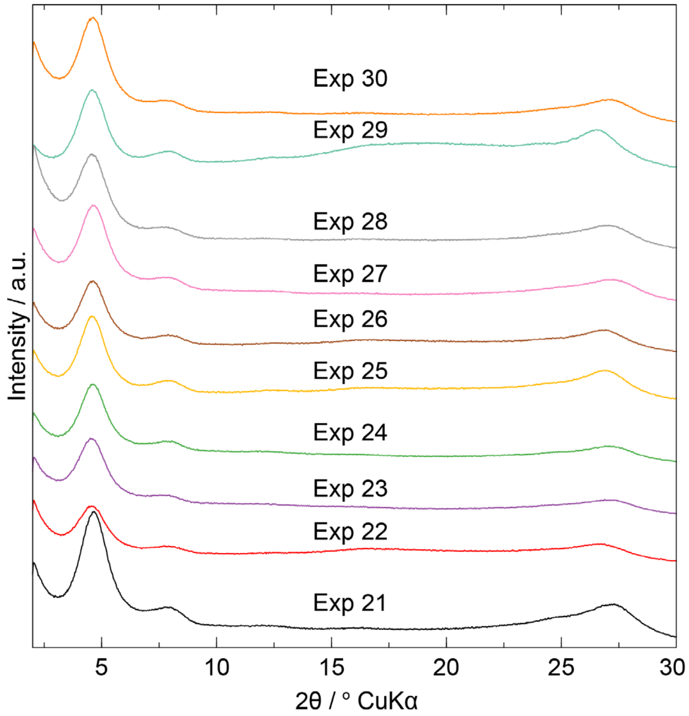

Figure S53. Powder X-ray diffraction analysis of COF-323 compounds synthesized under reaction conditions specified in Experiments 21 through 30.

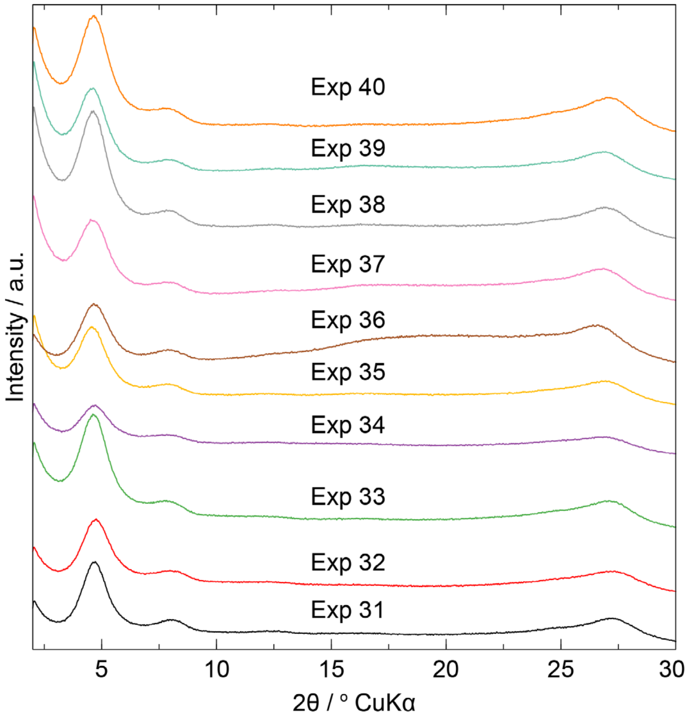

Figure S54. Powder X-ray diffraction analysis of COF-323 compounds synthesized under reaction conditions specified in Experiments 31 through 40.

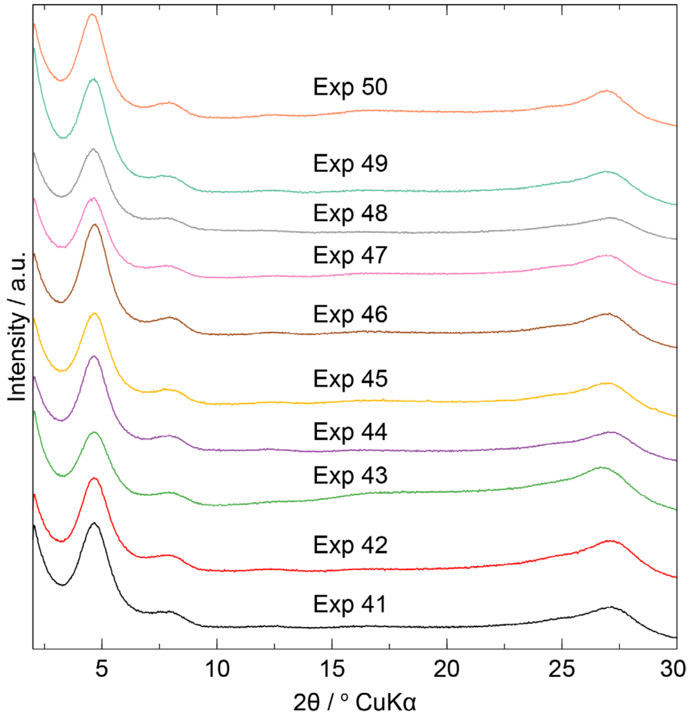

Figure S55. Powder X-ray diffraction analysis of COF-323 compounds synthesized under reaction conditions specified in Experiments 41 through 50.

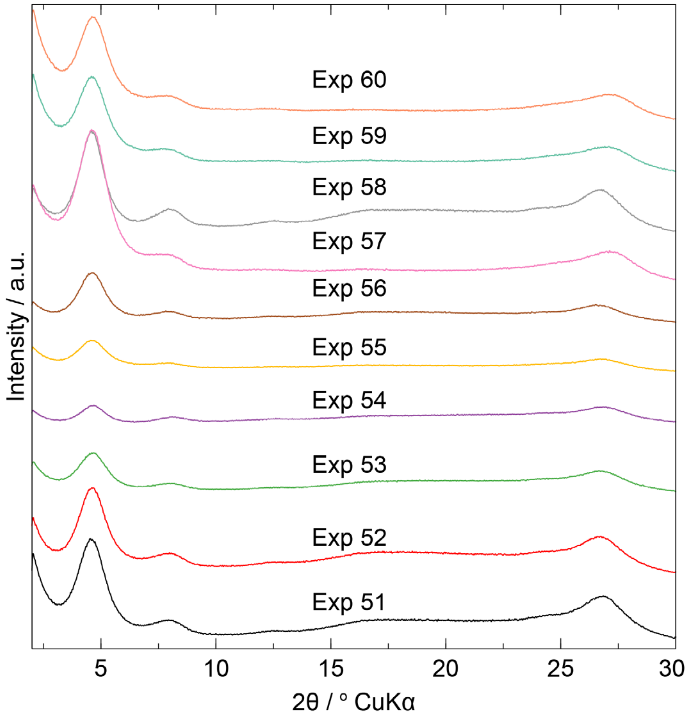

Figure S56. Powder X-ray diffraction analysis of COF-323 compounds synthesized under reaction conditions specified in Experiments 51 through 60.

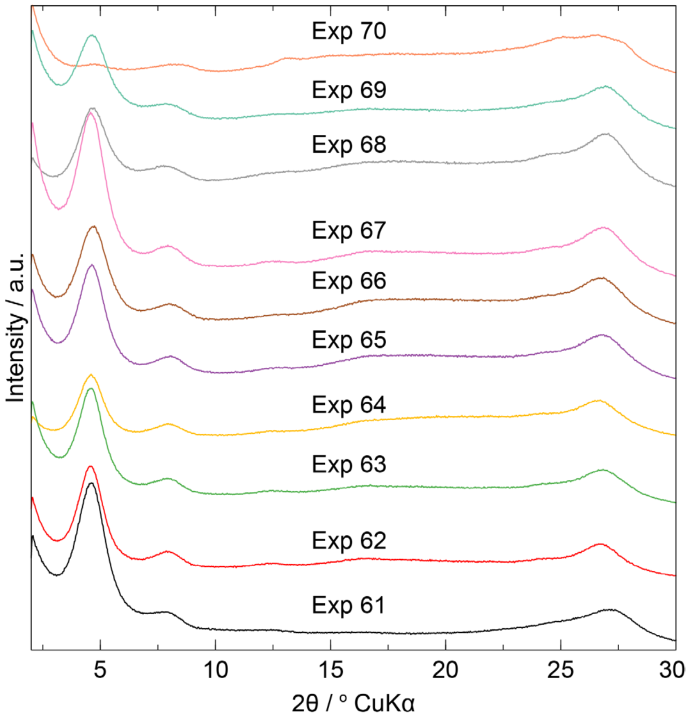

Figure S57. Powder X-ray diffraction analysis of COF-323 compounds synthesized under reaction conditions specified in Experiments 61 through 70.

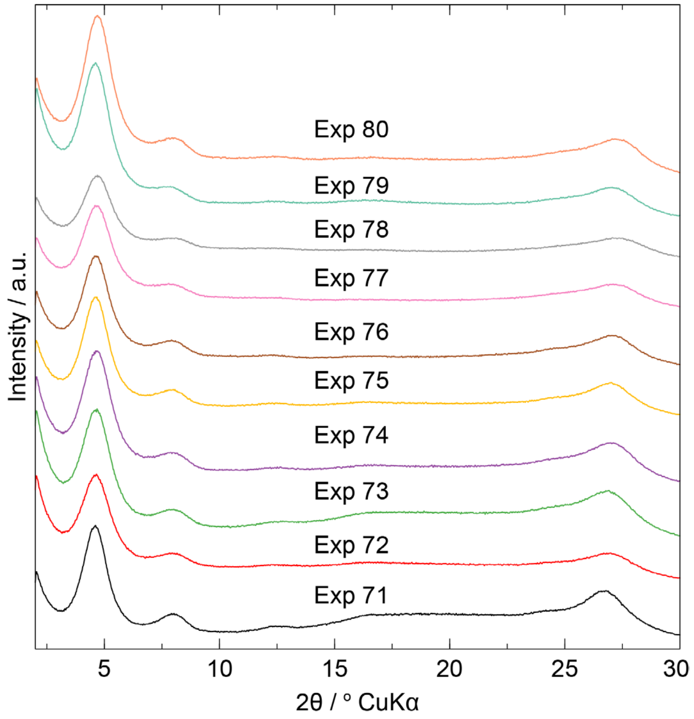

Figure S58. Powder X-ray diffraction analysis of COF-323 compounds synthesized under reaction conditions specified in Experiments 71 through 80.

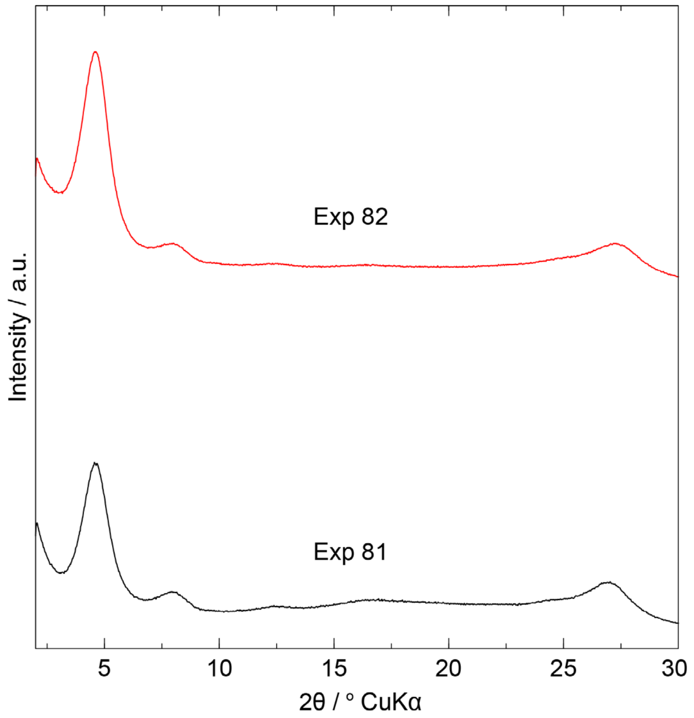

Figure S59. Powder X-ray diffraction analysis of COF-323 compounds synthesized under reaction conditions specified in Experiments 81 and 82.

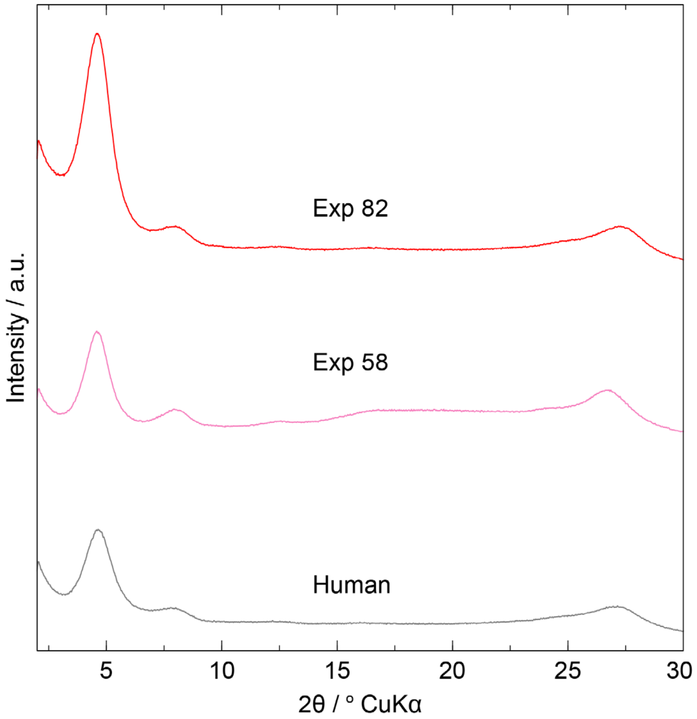

Figure S60. Overlay of the PXRD patterns of COF sample using conditions optimized by human and Experiments 58 and 82.

Section S6. Thermogravimetric Analysis

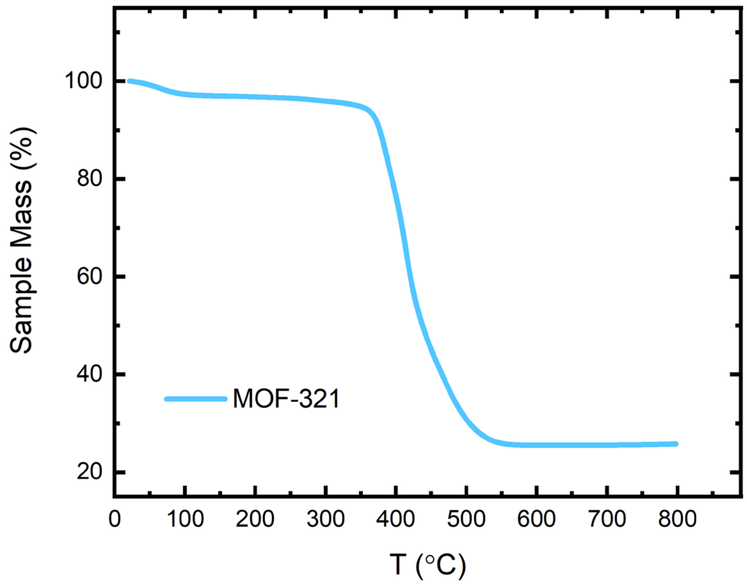

Figure S61. TGA trace of MOF-321 under nitrogen flow.

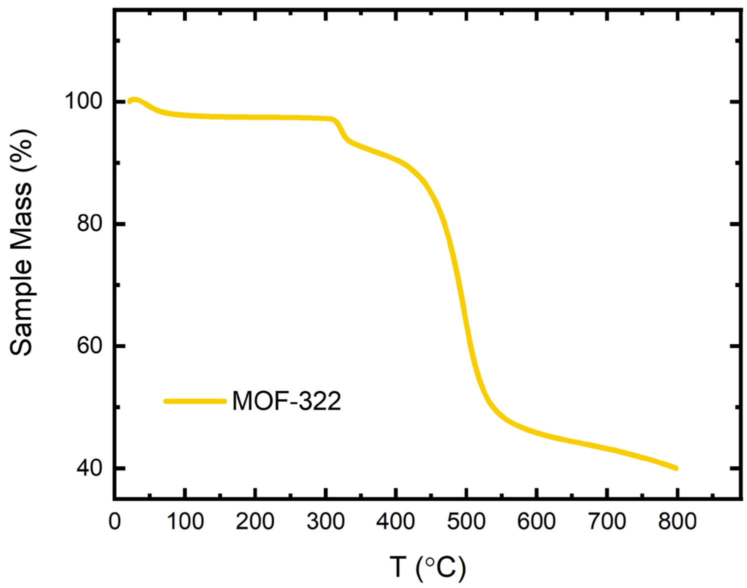

Figure S62. TGA trace of MOF-322 under nitrogen flow.

Section S7. Nitrogen Sorption Analysis

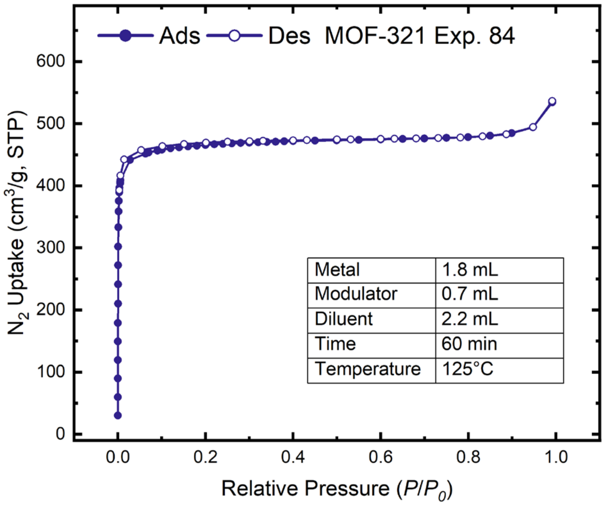

Figure S63. Nitrogen sorption isotherm at 77 K for MOF-321 synthesized using the microwave-assisted method. This condition yields a BET surface area of 1876 m^2 g^{-1}. *P*, nitrogen pressure; P_0 = 1 atm; Symbols of filled circles denote the adsorption branch, while empty circles denote the desorption branch.

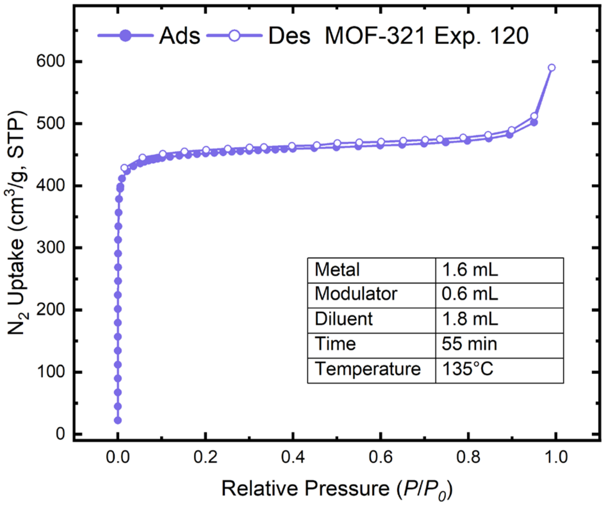

Figure S64. Nitrogen sorption isotherm at 77 K for MOF-321 synthesized using the microwave-assisted method. This condition yields a BET surface area of 1807 m^2 g^{-1}. *P*, nitrogen pressure; P_0 = 1 atm; Symbols of filled circles denote the adsorption branch, while empty circles denote the desorption branch.

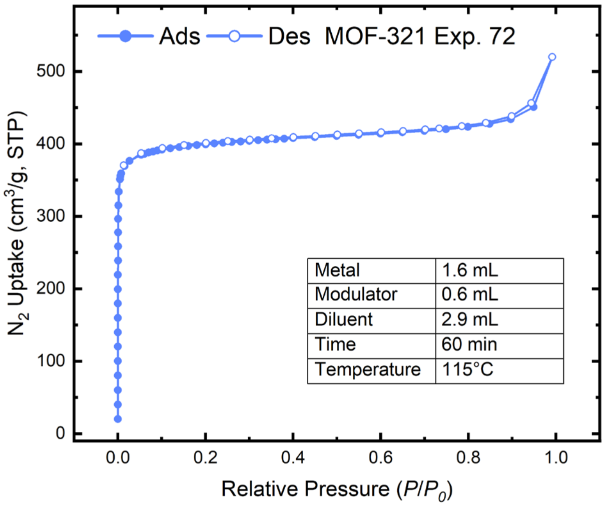

Figure S65. Nitrogen sorption isotherm at 77 K for MOF-321 synthesized using the microwave-assisted method. This condition yields a BET surface area of 1596 m^2 g^{-1}. P, nitrogen pressure; P_0 = 1 atm; Symbols of filled circles denote the adsorption branch, while empty circles denote the desorption branch.

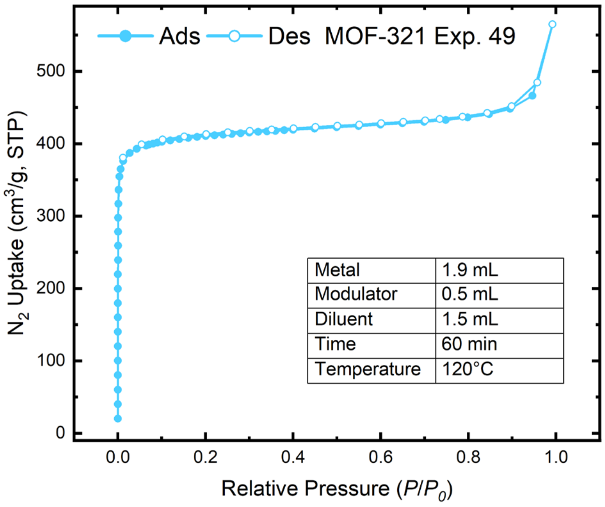

Figure S66. Nitrogen sorption isotherm at 77 K for MOF-321 synthesized using the microwave-assisted method. This condition yields a BET surface area of 1640 m^2 g^{-1}. P, nitrogen pressure; P_0 = 1 atm; Symbols of filled circles denote the adsorption branch, while empty circles denote the desorption branch.

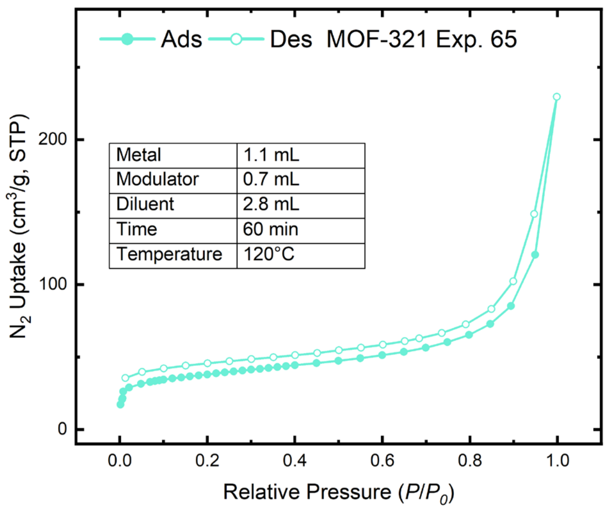

Figure S67. Nitrogen sorption isotherm at 77 K for MOF-321 synthesized using the microwave-assisted method. This condition yields a BET surface area of 137 m^2 g^{-1}. *P*, nitrogen pressure; P_0 = 1 atm; Symbols of filled circles denote the adsorption branch, while empty circles denote the desorption branch.

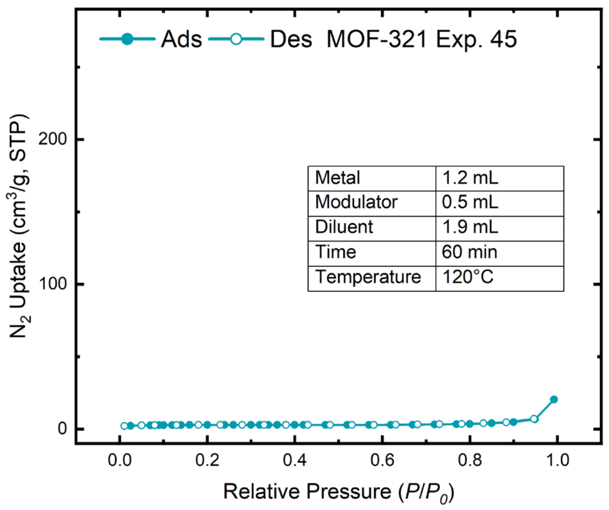

Figure S68. Nitrogen sorption isotherm at 77 K for MOF-321 synthesized using the microwave-assisted method. This condition yields a BET surface area of 11 m^2 g^{-1}. *P*, nitrogen pressure; P_0 = 1 atm; Symbols of filled circles denote the adsorption branch, while empty circles denote the desorption branch.

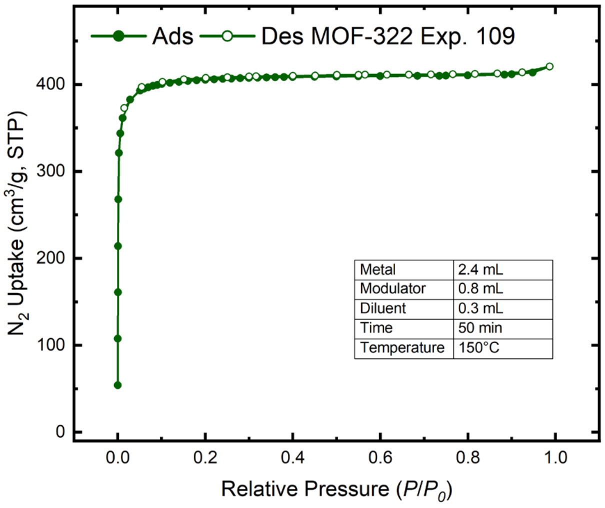

Figure S69. Nitrogen sorption isotherm at 77 K for MOF-322 synthesized using the microwave-assisted method. This condition yields a BET surface area of 1584 m^2 g^{-1}. *P*, nitrogen pressure; P_0 = 1 atm; Symbols of filled circles denote the adsorption branch, while empty circles denote the desorption branch.

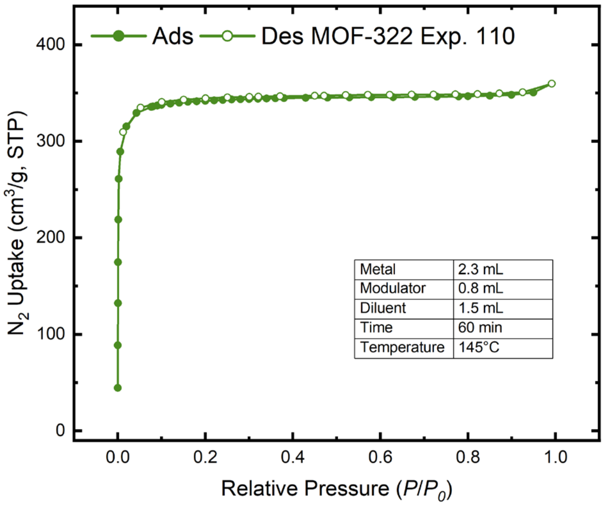

Figure S70. Nitrogen sorption isotherm at 77 K for MOF-322 synthesized using the microwave-assisted method. This condition yields a BET surface area of 1378 m^2 g^{-1}. *P*, nitrogen pressure; P_0 = 1 atm; Symbols of filled circles denote the adsorption branch, while empty circles denote the desorption branch.

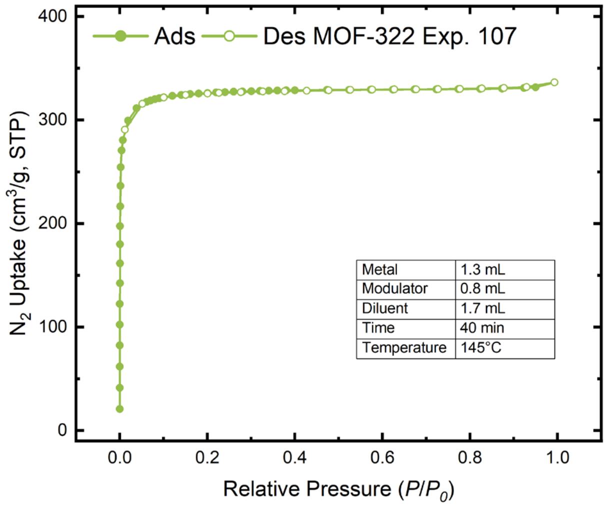

Figure S71. Nitrogen sorption isotherm at 77 K for MOF-322 synthesized using the microwave-assisted method. This condition yields a BET surface area of 1280 m^2 g^{-1}. *P*, nitrogen pressure; P_0 = 1 atm; Symbols of filled circles denote the adsorption branch, while empty circles denote the desorption branch.

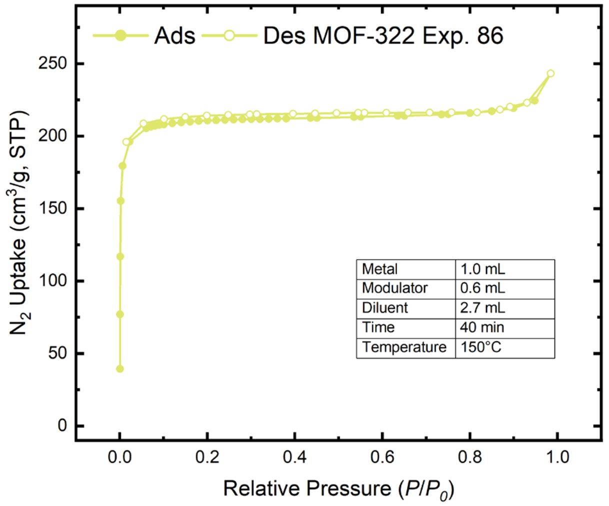

Figure S72. Nitrogen sorption isotherm at 77 K for MOF-322 synthesized using the microwave-assisted method. This condition yields a BET surface area of 854 m^2 g^{-1}. *P*, nitrogen pressure; P_0 = 1 atm; Symbols of filled circles denote the adsorption branch, while empty circles denote the desorption branch.

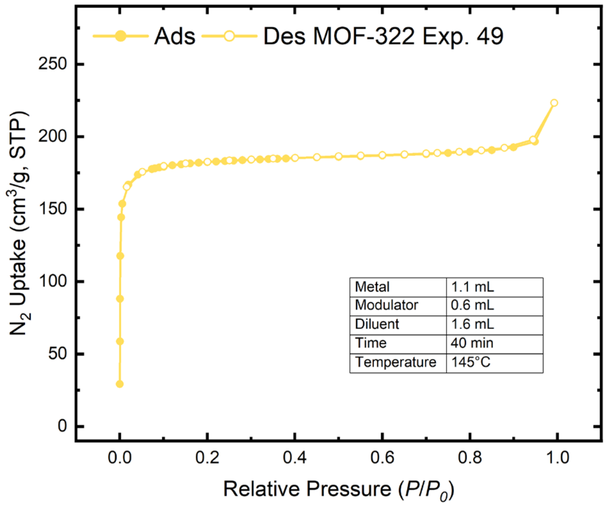

Figure S73. Nitrogen sorption isotherm at 77 K for MOF-322 synthesized using the microwave-assisted method. This condition yields a BET surface area of 731 m^2 g^{-1}. *P*, nitrogen pressure; P_0 = 1 atm; Symbols of filled circles denote the adsorption branch, while empty circles denote the desorption branch.

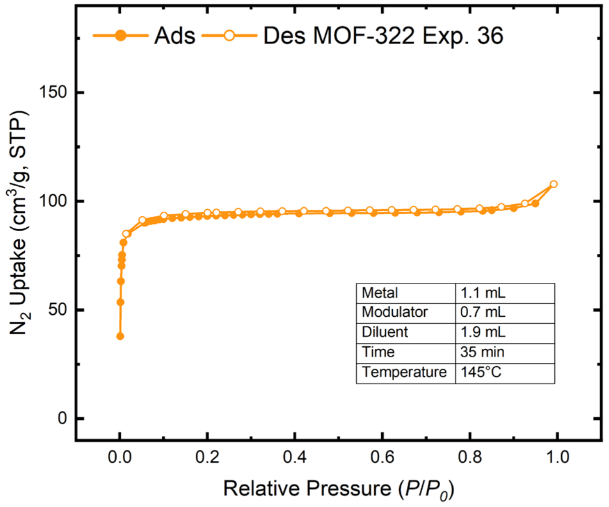

Figure S74. Nitrogen sorption isotherm at 77 K for MOF-322 synthesized using the microwave-assisted method. This condition yields a BET surface area of 376 m^2 g^{-1}. *P*, nitrogen pressure; P_0 = 1 atm; Symbols of filled circles denote the adsorption branch, while empty circles denote the desorption branch.

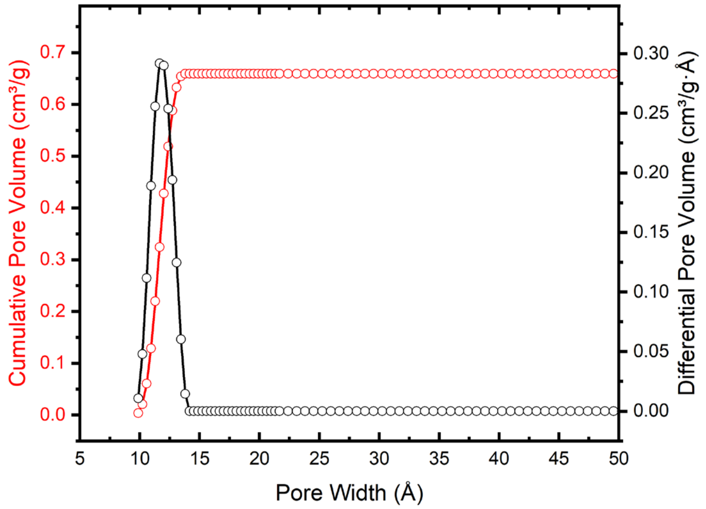

Figure S75. Pore size distribution of MOF-321 (Exp. 84) derived from N_2 sorption isotherm measured at 77 K, the adsorption branch was fitted with a quenched solid-state density functional theory (QSDFT) model of N_2 on carbon at 77 K (slit/cylindrical pores). Pore width = 11.8 Å.

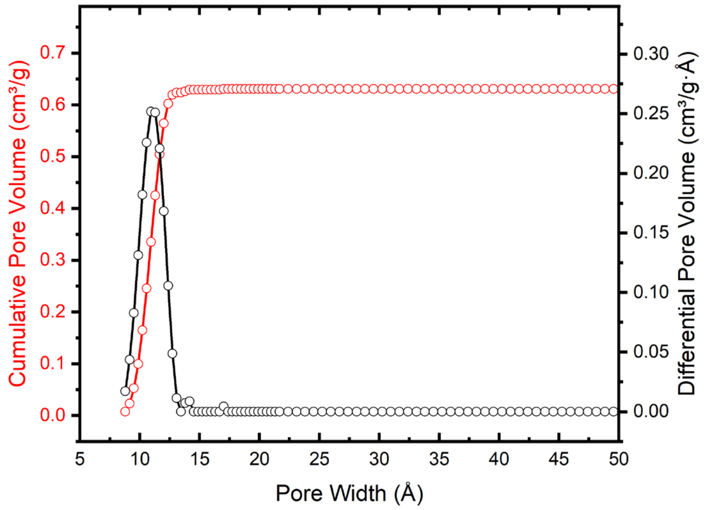

Figure S76. Pore size distribution of MOF-321 (Exp. 120) derived from N_2 sorption isotherm measured at 77 K, the adsorption branch was fitted with a quenched solid-state density functional theory (QSDFT) model of N_2 on carbon at 77 K (slit/cylindrical pores). Pore width = 11.1 Å.

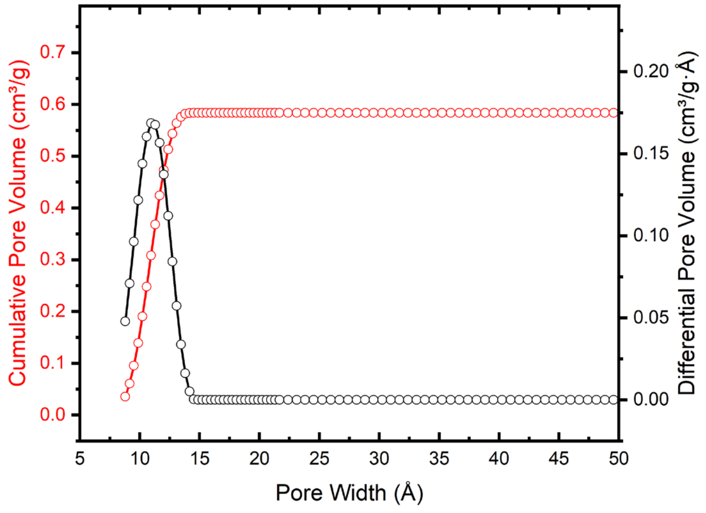

Figure S77. Pore size distribution of MOF-321 (Exp. 72) derived from N_2 sorption isotherm measured at 77 K, the adsorption branch was fitted with a quenched solid-state density functional theory (QSDFT) model of N_2 on carbon at 77 K (slit/cylindrical pores). Pore width = 11.1 Å.

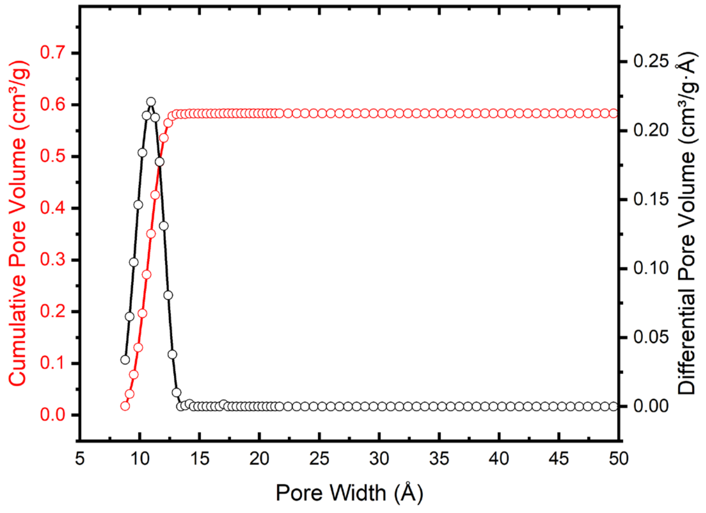

Figure S78. Pore size distribution of MOF-321 (Exp. 49) derived from N_2 sorption isotherm measured at 77 K, the adsorption branch was fitted with a quenched solid-state density functional theory (QSDFT) model of N_2 on carbon at 77 K (slit/cylindrical pores). Pore width = 11.0 Å.

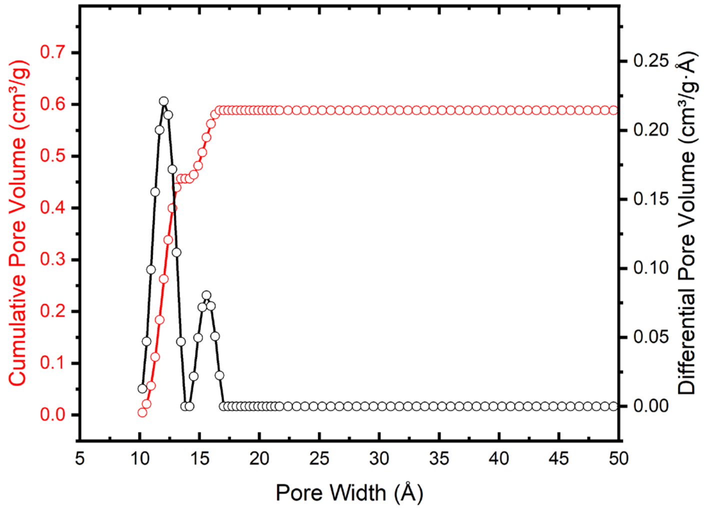

Figure S79. Pore size distribution of MOF-322 (Exp. 109) derived from N_2 sorption isotherm measured at 77 K, the adsorption branch was fitted with a quenched solid-state density functional theory (QSDFT) model of N_2 on carbon at 77 K (slit/cylindrical pores). Pore width = 12.1 Å.

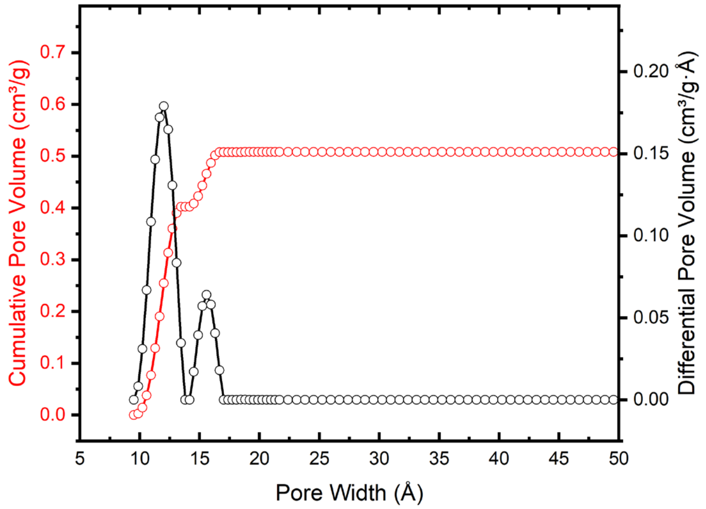

Figure S80. Pore size distribution of MOF-322 (Exp. 110) derived from N_2 sorption isotherm measured at 77 K, the adsorption branch was fitted with a quenched solid-state density functional theory (QSDFT) model of N_2 on carbon at 77 K (slit/cylindrical pores). Pore width = 12.0 Å.

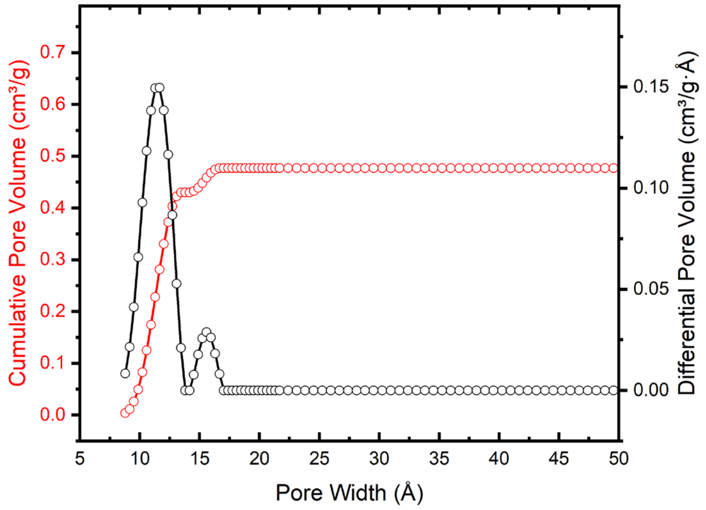

Figure S81. Pore size distribution of MOF-322 (Exp. 107) derived from N_2 sorption isotherm measured at 77 K, the adsorption branch was fitted with a quenched solid-state density functional theory (QSDFT) model of N_2 on carbon at 77 K (slit/cylindrical pores). Pore width = 11.5 Å.

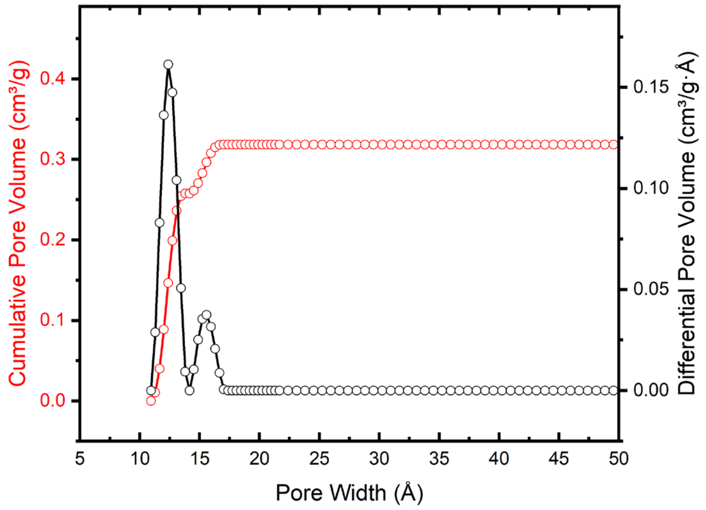

Figure S82. Pore size distribution of MOF-322 (Exp. 86) derived from N_2 sorption isotherm measured at 77 K, the adsorption branch was fitted with a quenched solid-state density functional theory (QSDFT) model of N_2 on carbon at 77 K (slit/cylindrical pores). Pore width = 12.4 Å.

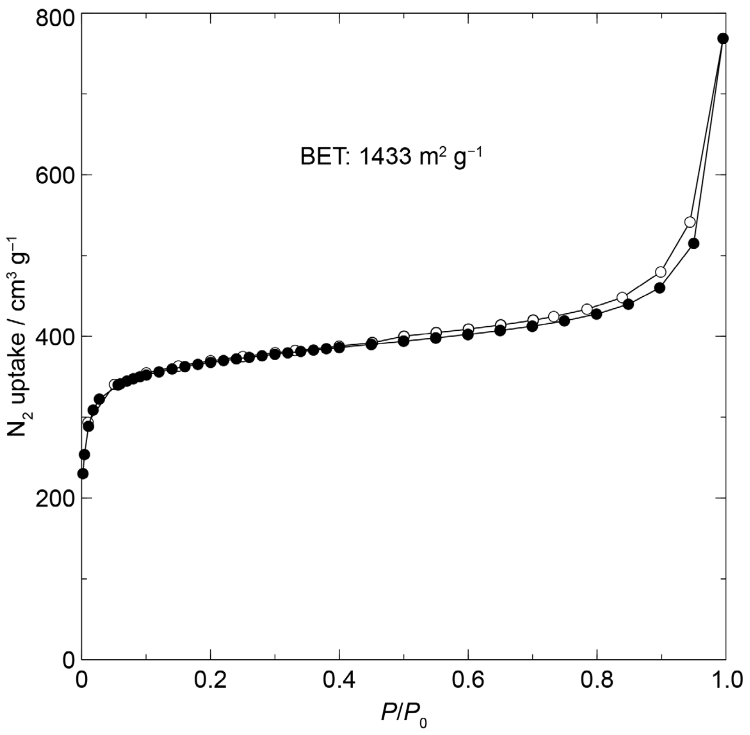

Figure S83. N_2 sorption isotherm at 77 K of activated COF-323. The filled and open circles represent the adsorption and desorption branches, respectively.

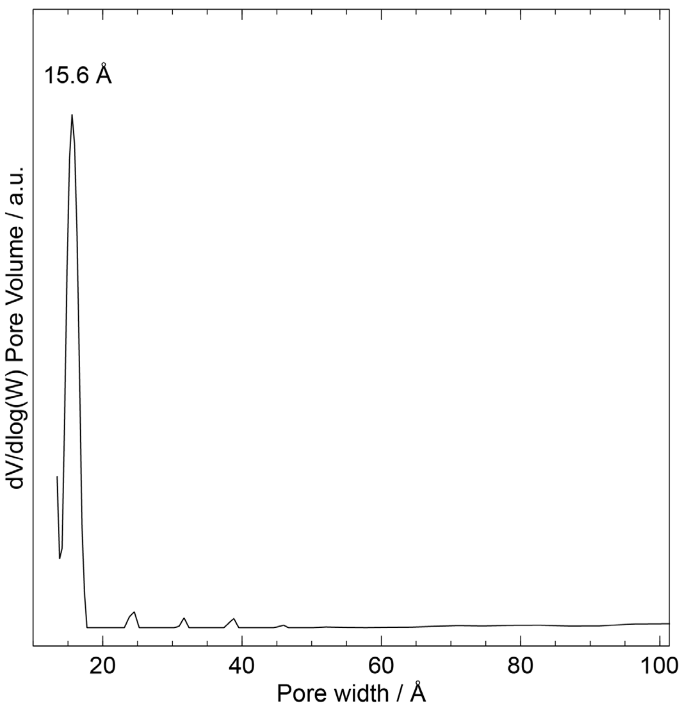

Figure S84. Pore size distributions of COF-323. The N_2-Cylindrical Pores-Oxide Surface model was used indicating a pore width of 15.6 Å.

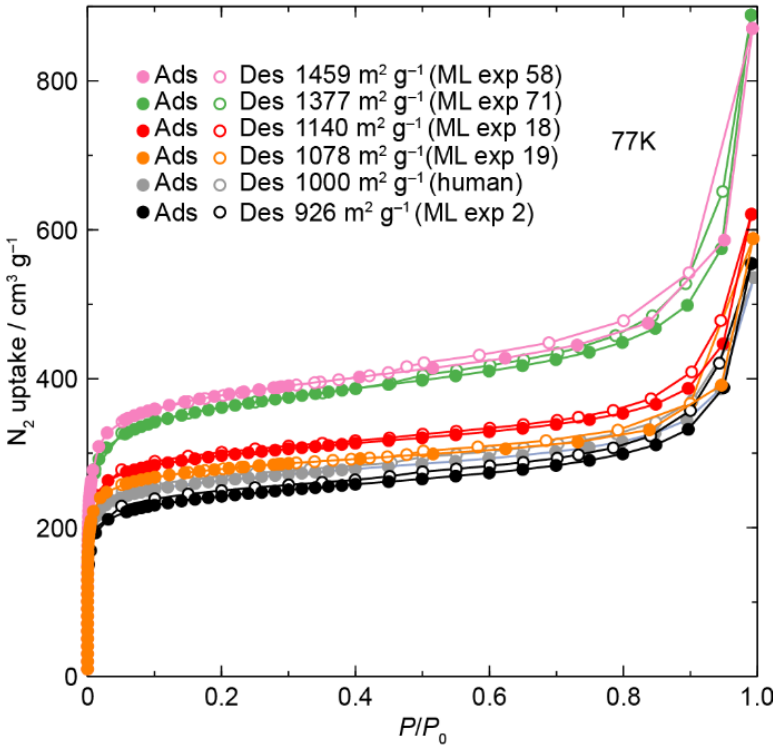

Figure S85. N_2 sorption isotherms of the representative COF samples measured at 77 K.

Section S8. Water Sorption Analysis

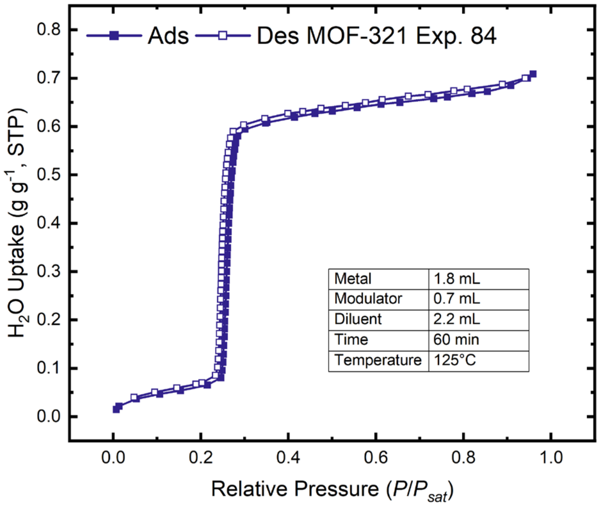

Figure S86. Water vapor sorption isotherms of MOF-321, which was synthesized utilizing the microwave-assisted method during Experiment 84, plotted against relative humidity (P/P_{sat}). P, water vapor pressure; P_{sat}, saturation water vapor pressure at 25°C.

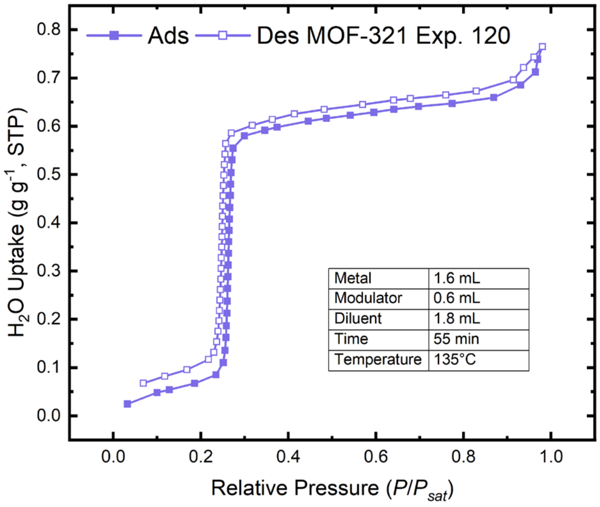

Figure S87. Water vapor sorption isotherms of MOF-321, which was synthesized utilizing the microwave-assisted method during Experiment 120, plotted against relative humidity (P/P_{sat}). P, water vapor pressure; P_{sat}, saturation water vapor pressure at 25°C.

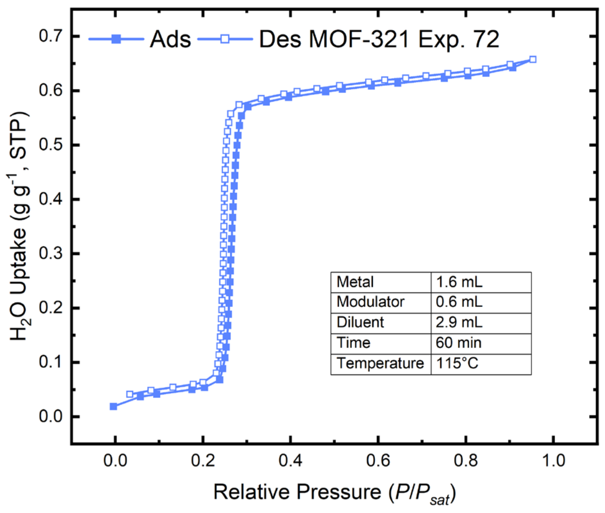

Figure S88. Water vapor sorption isotherms of MOF-321, which was synthesized utilizing the microwave-assisted method during Experiment 72, plotted against relative humidity (P/P_{sat}). P, water vapor pressure; P_{sat}, saturation water vapor pressure at the respective analysis temperature.

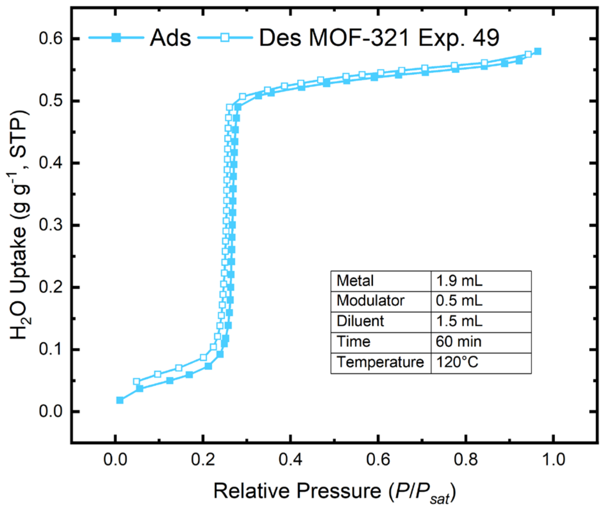

Figure S89. Water vapor sorption isotherms of MOF-321, which was synthesized utilizing the microwave-assisted method during Experiment 49, plotted against relative humidity (P/P_{sat}). P, water vapor pressure; P_{sat}, saturation water vapor pressure at 25°C.

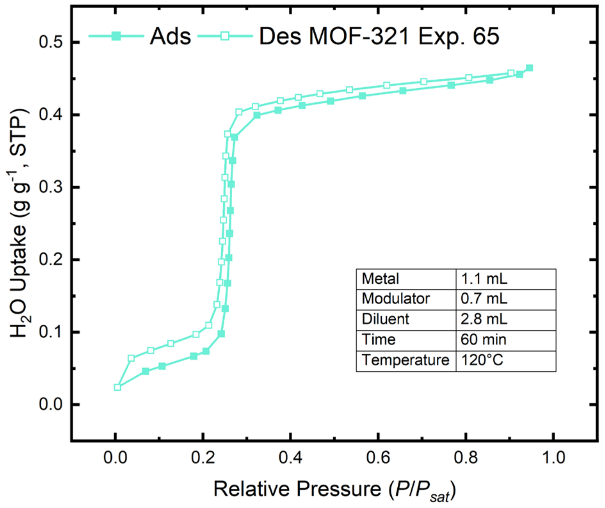

Figure S90. Water vapor sorption isotherms of MOF-321, which was synthesized utilizing the microwave-assisted method during Experiment 65, plotted against relative humidity (P/P_{sat}). P, water vapor pressure; P_{sat}, saturation water vapor pressure at 25°C.

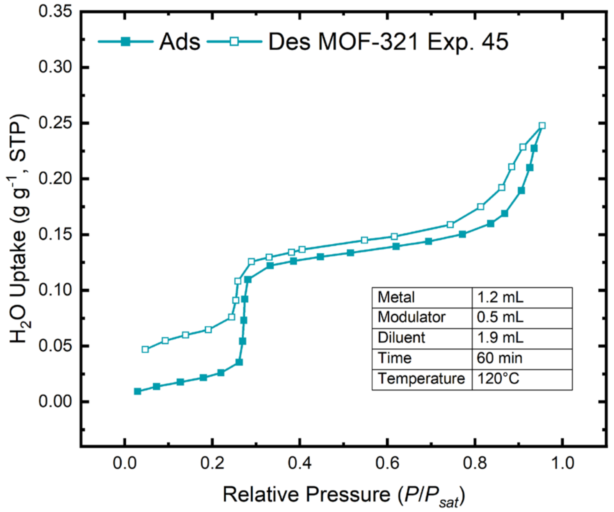

Figure S91. Water vapor sorption isotherms of MOF-321, which was synthesized utilizing the microwave-assisted method during Experiment 45, plotted against relative humidity (P/P_{sat}). P, water vapor pressure; P_{sat}, saturation water vapor pressure at 25°C.

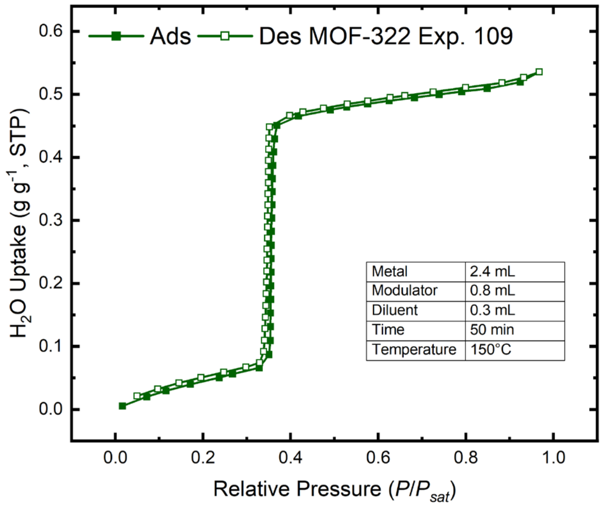

Figure S92. Water vapor sorption isotherms of MOF-322, which was synthesized utilizing the microwave-assisted method during Experiment 109, plotted against relative humidity (P/P_{sat}). P, water vapor pressure; P_{sat}, saturation water vapor pressure at 25°C.

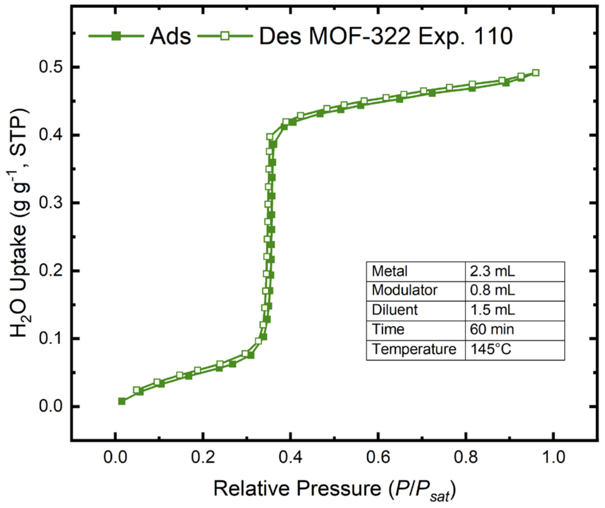

Figure S93. Water vapor sorption isotherms of MOF-322, which was synthesized utilizing the microwave-assisted method during Experiment 110, plotted against relative humidity (P/P_{sat}). P, water vapor pressure; P_{sat}, saturation water vapor pressure at 25°C.

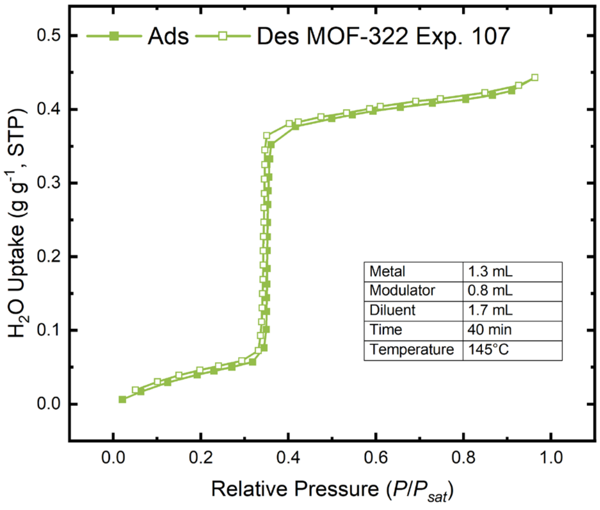

Figure S94. Water vapor sorption isotherms of MOF-322, which was synthesized utilizing the microwave-assisted method during Experiment 107, plotted against relative humidity (P/P_{sat}). P, water vapor pressure; P_{sat}, saturation water vapor pressure at 25°C.

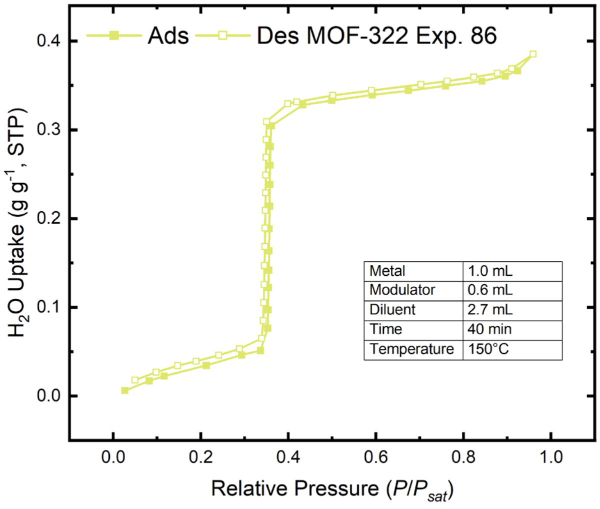

Figure S95. Water vapor sorption isotherms of MOF-322, which was synthesized utilizing the microwave-assisted method during Experiment 86, plotted against relative humidity (P/P_{sat}). P, water vapor pressure; P_{sat}, saturation water vapor pressure at 25°C.

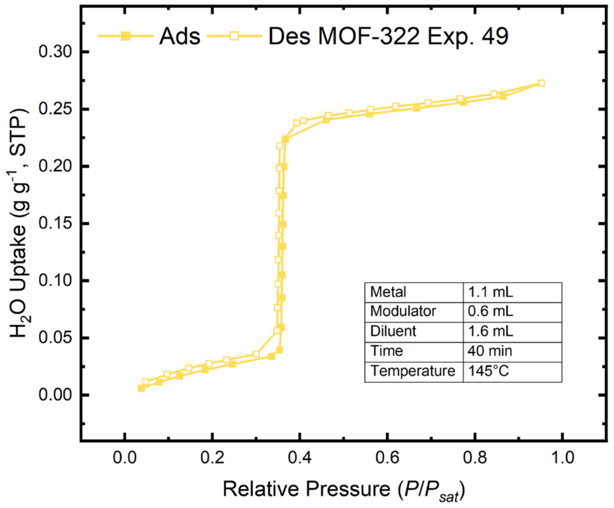

Figure S96. Water vapor sorption isotherms of MOF-322, which was synthesized utilizing the microwave-assisted method during Experiment 49, plotted against relative humidity (P/P_{sat}). P, water vapor pressure; P_{sat}, saturation water vapor pressure at 25°C.

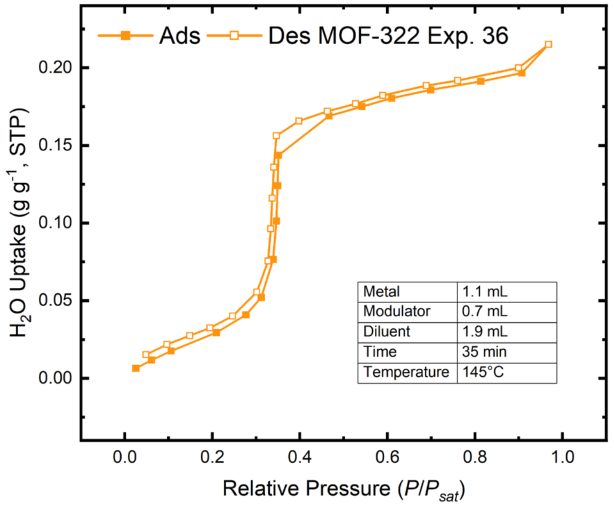

Figure S97. Water vapor sorption isotherms of MOF-322, which was synthesized utilizing the microwave-assisted method during Experiment 36, plotted against relative humidity (P/P_{sat}). P, water vapor pressure; P_{sat}, saturation water vapor pressure at 25°C.

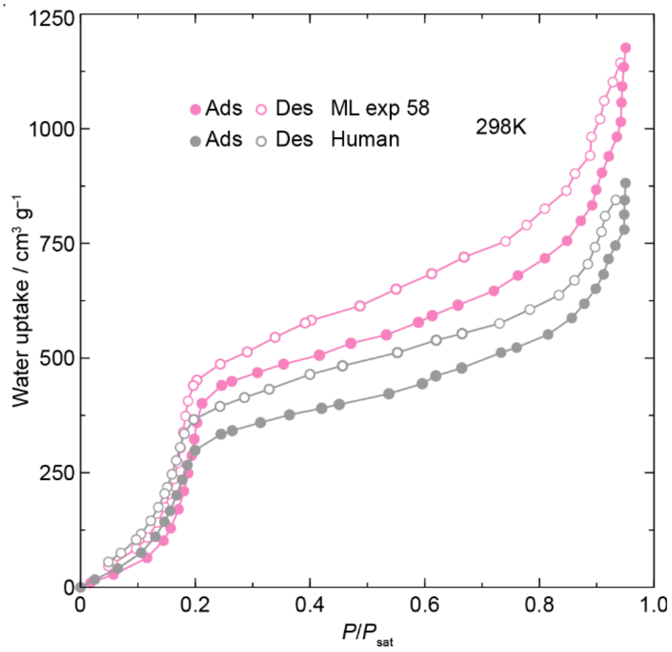

Figure S98. Comparison of water sorption analysis between COF-323 synthesized under machine learning suggested conditions and the best version prepared by a human after exhaustive screening efforts involving numerous experimental conditions. *P*: Water vapor pressure. P_{sat}: The saturation vapor pressure at the respective temperature. The filled and open circles represent the adsorption and desorption points, respectively.

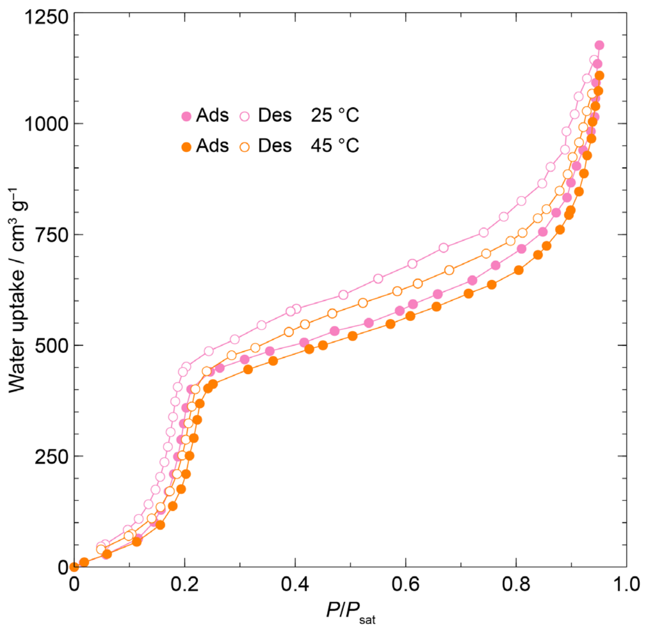

Figure S99. Water sorption analysis of COF-323 (Exp. 58) at different temperatures (25 °C and 45 °C). *P*: Water vapor pressure. P_{sat}: The saturation vapor pressure at the respective temperature. The filled and open circles represent the adsorption and desorption points, respectively.

Section S9. Bayesian Optimization Method

MOF Crystallinity Optimization

Given the large parameter space, with five synthesis variables each featuring a broad range and small increments, as detailed in Section S4, it is laborious and time consuming to fully explore the 6,101,172 potential conditions in search of the optimal crystallinity index for MOF-321 and MOF-322. Therefore we applied a Bayesian optimization (BO) algorithm to efficiently sample the synthesis parameters, since BO is known for finding the global optimum of a black box objective function $f(x)$ in a minimum number of steps[10], and since it has shown previous success in property prediction and synthesis optimization for material discovery.[11-16] The algorithm builds a surrogate model that better approximates the objective function $f(x)$, i.e., crystallinity index, over the search space x, defined by the metal amount, modulator amount, solvent amount, reaction time and temperature, through incorporating and updating prior belief about $f(x)$ with samples directed by an acquisition function for the most promising set of parameters to inform subsequent experiments. We used Expected Improvement (EI) as our acquisition function, defined as

$$EI(x) = \begin{cases} (\mu(x) - f_{max}(x) - \xi)\Phi(Z) + \sigma(x)\varphi(Z), & \sigma(x) > 0 \\ 0, & \sigma(x) = 0 \end{cases}$$

where

$$Z = \begin{cases} (\mu(x) - f_{max}(x) - \xi)/\sigma(x), & \sigma(x) > 0 \\ 0, & \sigma(x) = 0 \end{cases}$$

where $\mu(x)$ and $\sigma(x)$ are the mean and the standard deviation of the model posterior. Φ and φ denote the cumulative density function (CDF) and probability density function (PDF) of the standard normal distribution. The function balances exploitation of high predicted objective and exploration of areas where the prediction uncertainty is high, with parameter ξ, set to 0.01 in our experiments. A random forest (RF) model with 100 tree estimators was used as the surrogate model due to its capacity to handle both the categorical and continuous synthesis parameters, through other alternatives exist such as Gaussian Process and Bayesian neural networks.[15, 16] The key parameters in the synthesis include the volume of metal and stock solution, the additional solvent (water) to vary the total concentration, the reaction time and temperature (Section S4, Supporting Information), since these parameters were hypothesized to have impact on the crystallinity of the resulting MOFs.

The initial data set consists of 12 experiments, the synthesis conditions of which were randomly drawn from the entire search space and their crystallinity index were determined after measuring the PXRD (Section S5, Supporting Information). With the initial data points, the RF model is first trained to evaluate the EI and the maximum of this function was used as the next suggested experiment. For experiments parallelization, we adopted the constant liar strategy[17] where the previously suggested experiment is presumed to yield an objective value of the current maximum to update the model and generate the next set of parameters in one iteration. Here we used a batch

size of 3, and the RF model was iteratively updated with the experiment results to query for the next batch.

The performance of the model after 36 iterations is summarized in this section for MOF-321 and MOF-322, respectively. Both show an overall correspondence between the best synthesis parameters and the predicted crystallinity index maximum (Figure S100 and Figure S103). The lower diagonal shows how a given pair of synthesis parameters affects the predicted crystallinity index when the influence of all others is averaged out. The bright yellow region corresponds to the highest averaged predicted crystallinity index under a given pair of parameters, whereas the dark blue is the vice versa. The red stars indicate the synthesis parameters that yielded the highest crystallinity index, and the black dots mark all sampled parameters during the BO process. The plots on the diagonal show the distribution of the averaged predicted crystallinity index over each of the 5 single synthesis parameters, with the red dashed line marking the best synthesis parameters. All partial dependencies were evaluated from 250 random samples at each point. In addition, we evaluated the importance of each synthesis parameter by examining its impact on reducing the mean squared error of the predicted crystallinity. This was achieved by using the parameter for splitting within each decision tree (Figure S101 and Figure S104). Notably, for both MOFs, the amount of metal stock solution and the additional solvent added were identified as the most influential parameters. These are directly associated with the metal-to-linker ratio and concentration level, respectively. Therefore, these two parameters should receive special attention when optimizing synthesis conditions.

The evolution of each synthesis parameter as a function of experiment number was plotted (Figure S102 and Figure S105). In the case of MOF-321, parameters such as metal amount, solvent amount, and reaction time display a narrower range that correlates with successful crystal formation. The sampling of these variables quickly converged around the best synthesis parameters. Conversely, for MOF-322, the model took advantage of the high crystallinity region in relation to temperature. However, the wide distributions of sampled modulator amount and reaction time suggest attempts that were more exploratory than exploitative. These results underscore the unique synthesis conditions necessary to produce highly crystalline MOF-321 and MOF-322, respectively. The findings not only suggest our model's capability of pinpointing the optimal conditions within a limited number of experiments, but also demonstrate its applicability to MOFs with diverse synthesis conditions. Crucially, this can be achieved even when starting with no input of chemical knowledge or intuition, further attesting to the model's robustness in identifying optimal MOF synthesis conditions.

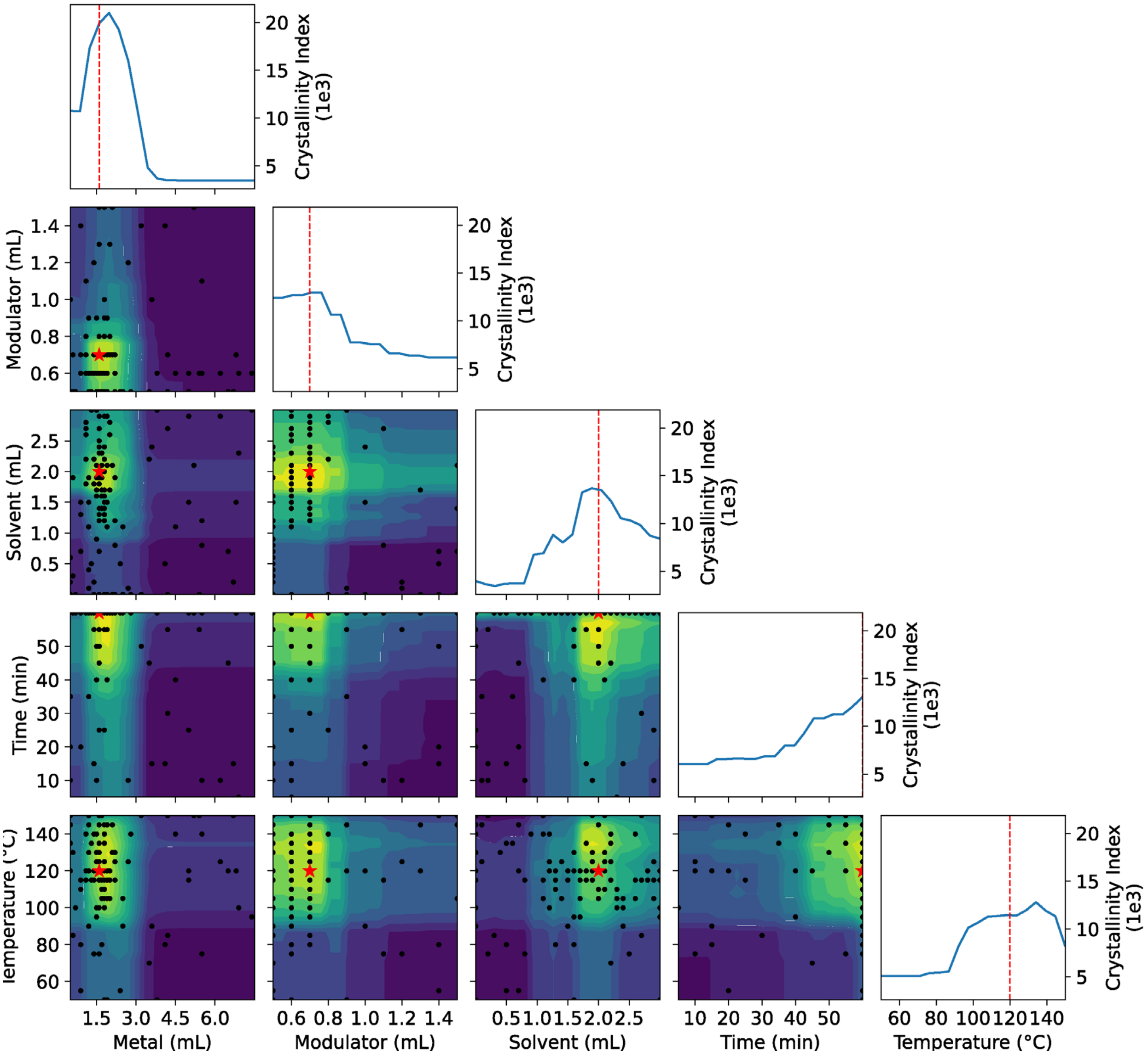

Figure S100. The partial dependence of predicted crystallinity index on synthesis parameters for MOF-321 at the end of the optimization.

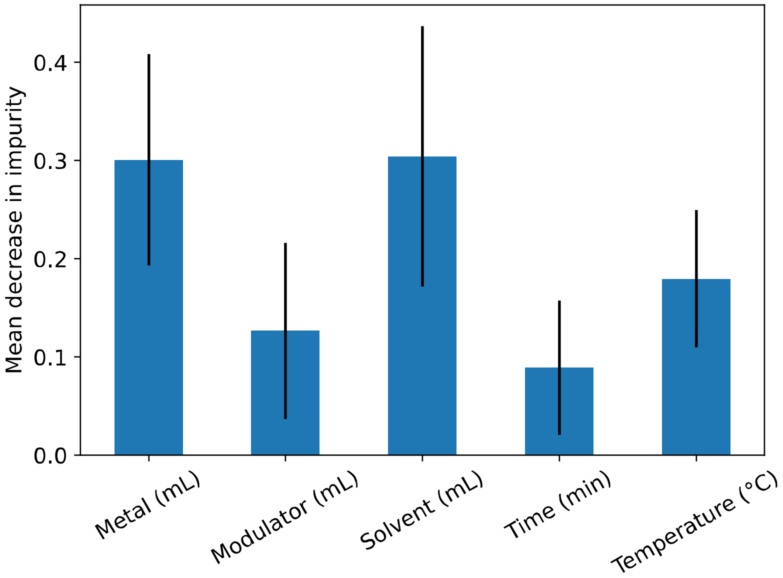

Figure S101. Feature importance of synthesis parameters of MOF-321. 'Metal' represents the amount of $AlCl_3$ stock solution added and is associated with the metal-to-linker ratio. 'Modulator' represents the amount of NaOH stock solution added. 'Solvent' represents the amount of additional DI water added to the mixture, influencing the concentration level. 'Time' and 'Temperature' pertain to microwave synthesis conditions.

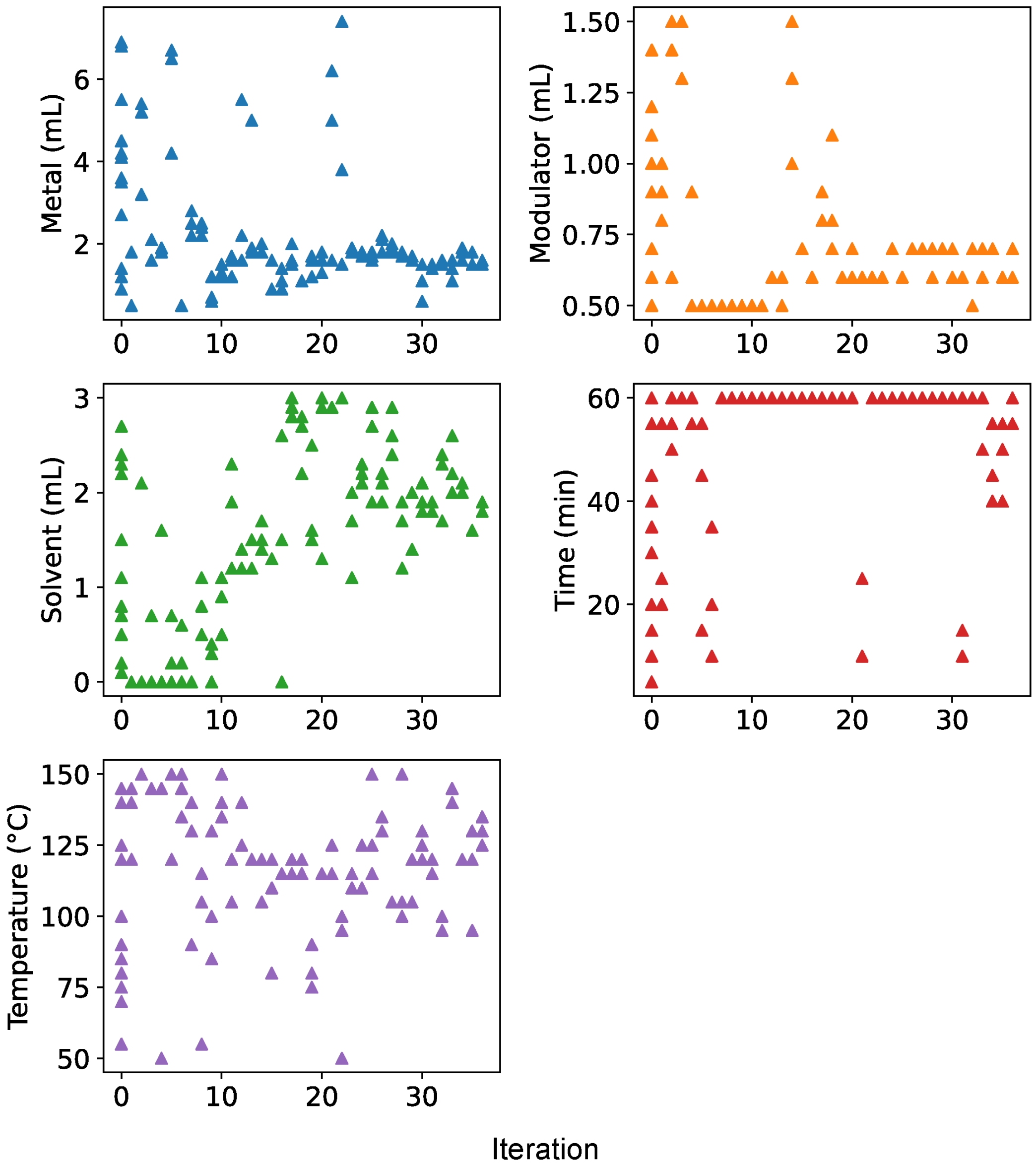

Figure S102. The evolution of synthesis parameters for MOF-321.

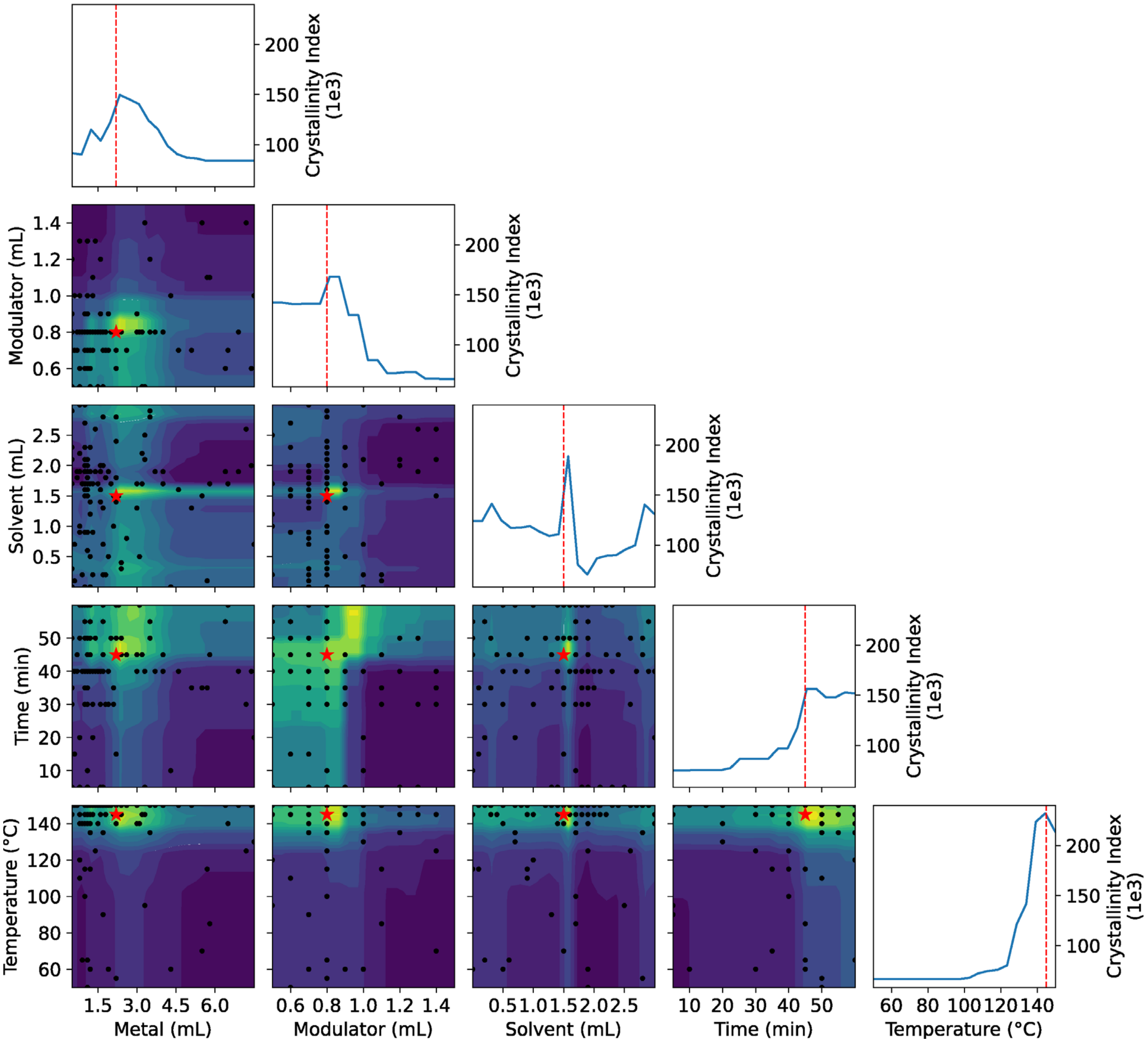

Figure S103. The partial dependence of predicted crystallinity index on synthesis parameters for MOF-322 at the end of the optimization.

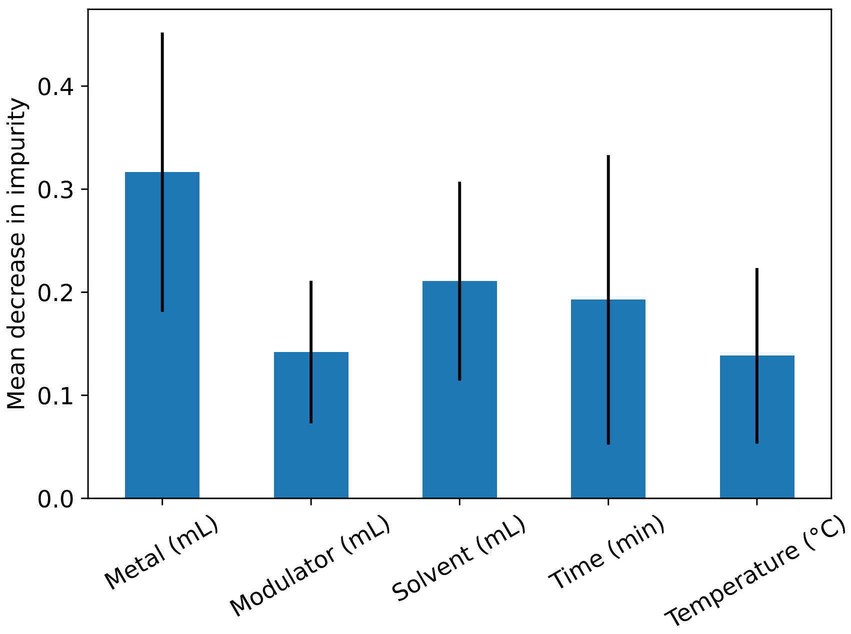

Figure S104. Feature importance of synthesis parameters of MOF-322. 'Metal' represents the amount of $AlCl_3$ stock solution added and is associated with the metal-to-linker ratio. 'Modulator' represents the amount of NaOH stock solution added. 'Solvent' represents the amount of additional DI water added to the mixture, influencing the concentration level. 'Time' and 'Temperature' pertain to microwave synthesis conditions.

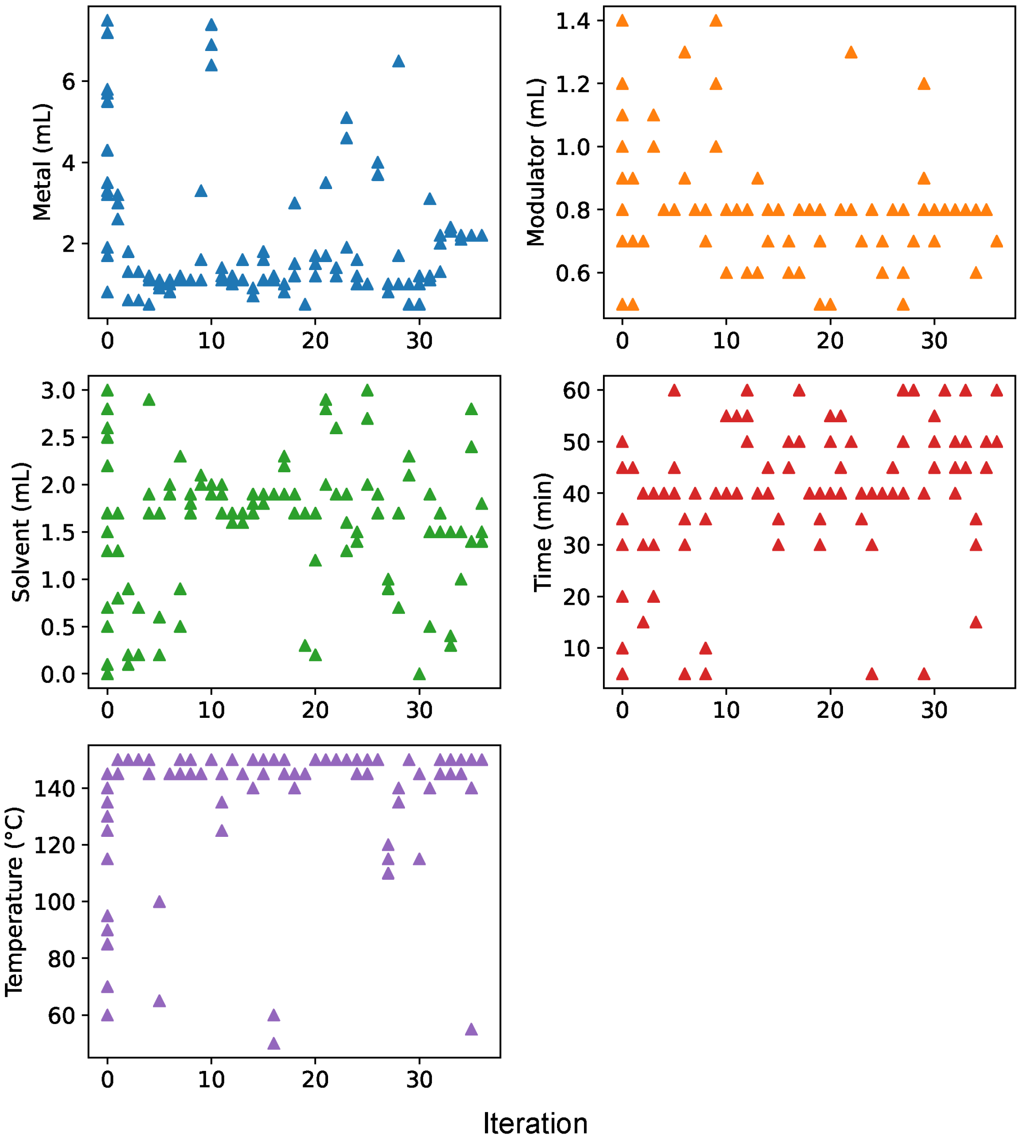

Figure S105. The evolution of synthesis parameters for MOF-322.

COF Crystallinity Optimization

The synthesis parameter search space for COF-323 is delineated by the different solvent combinations, the quantity of the acid modulator and dioxane solvent, reaction time, and temperature. An initial dataset of experiment outcomes of 10 randomly selected set of parameters was used to train a probabilistic surrogate model that approximates the objective crystallinity values accounting for uncertainties. The code used for MOF crystallinity optimization was adapted for a COF target by modifying the synthesis parameter boundary and definition.

The sampling trajectory of each synthesis parameter as a function of experiment number is illustrated in fig. 103. Each experiment is color coded by its measured crystallinity index, whose value is shown to be dominated by intensity. While the spread of the sampled solvent volumes and solvent mixture arrangements implies an uncertainty-guided search for these parameters, the model exploited the modulator volume and reaction temperature ranges that correspond to high crystallinity. In particular, the last 20 iterations showed steady improvement in terms of crystallinity index (see PXRD in Section S5), when the model concentrated its sampling of the 3 parameters. The optimized surrogate model prediction also demonstrated a good agreement with the measured crystallinity index, with a 96.2% pearson correlation. This strategy, contrasting with traditional human trial-and-error methods—typically adjusting only one or two synthesis parameters while keeping others constant—enables simultaneous modification of all five variables, targeting enhanced crystallinity. Moreover, the results underscore the model's proficiency in identifying optimal conditions within a restricted number of experiments and demonstrate the versatility in handling COF with varied synthesis conditions. Importantly, these feats are attainable without the need for prior chemical knowledge or intuition, which further substantiates the model's robustness in determining optimal synthesis conditions for COFs.

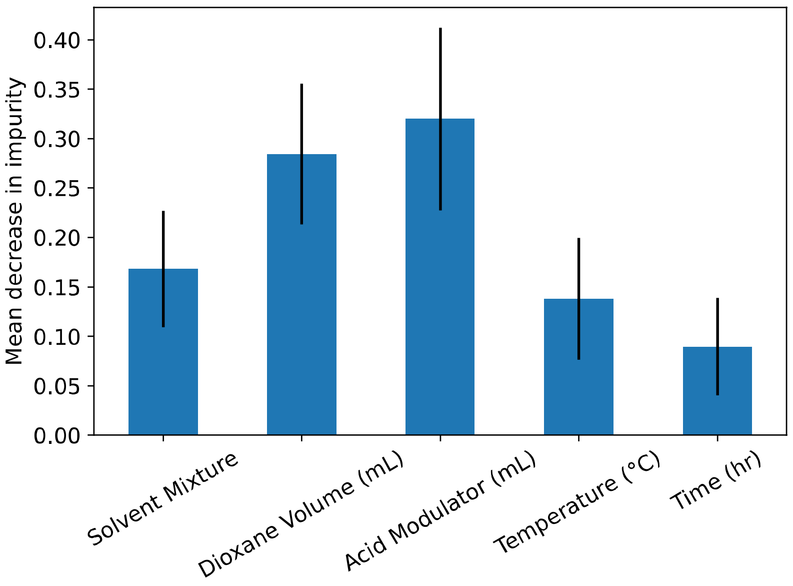

Figure S106. Feature importance of synthesis parameters of COF-323. 'Solvent Mixture' represents the choice of the solvent system for the synthesis. 'Dioxane Volume' represents the volume of dioxane in the solvent mixture (with the total volume set to 1 mL), influencing the volume ratios between two solvents. 'Acid Modulator' represents the amount of acid modulator added to the mixture. 'Time' and 'Temperature' pertain to microwave synthesis conditions.

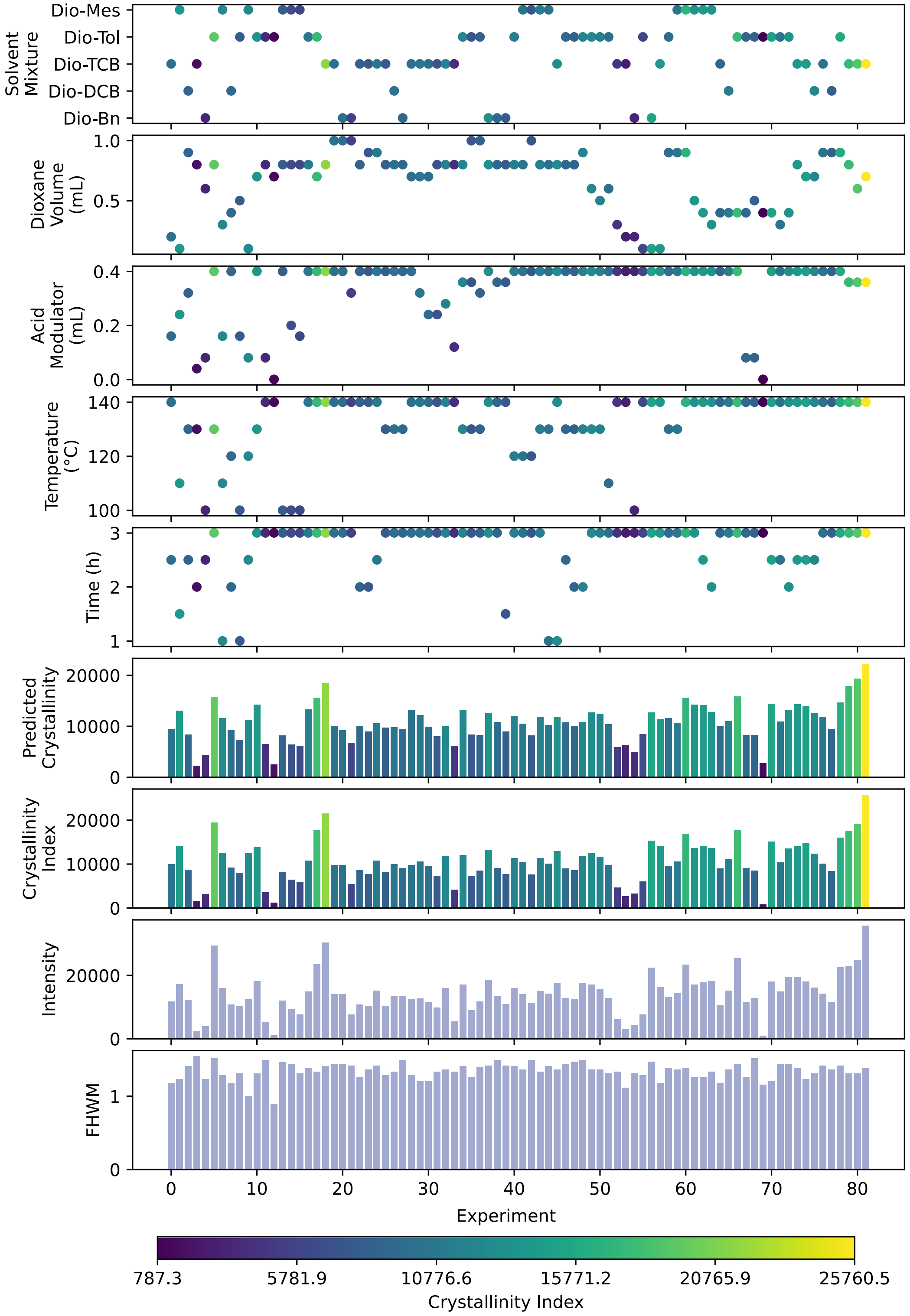

Figure S107. The sampling of synthesis parameters of COF-323 and their measured and predicted crystallinity as a function of experiment number.

References

(1) Vaswani, A.; Shazeer, N.; Parmar, N.; Uszkoreit, J.; Jones, L.; Gomez, A. N.; Kaiser, Ł.; Polosukhin, I., Attention is all you need. *NIPS* **2017,** *30*.

(2) Zheng, Z.; Rong, Z.; Rampal, N.; Borgs, C.; Chayes, J. T.; Yaghi, O. M., A GPT-4 Reticular Chemist for Guiding MOF Discovery. *Angew. Chem. Int. Ed.* **2023**, e202311983.

(3) Zheng, Z.; Zhang, O.; Borgs, C.; Chayes, J. T.; Yaghi, O. M., ChatGPT Chemistry Assistant for Text Mining and Prediction of MOF Synthesis. *J. Am. Chem. Soc.* **2023,** *145* (32), 18048–18062.

(4) Kong, Y.-R.; Zhang, R.; Zhang, J.; Luo, H.-B.; Liu, Y.; Zou, Y.; Ren, X.-M., Microwave-assisted rapid synthesis of nanoscale MOF-303 for hydrogel composites with superior proton conduction at ambient-humidity conditions. *ACS Appl. Energy Mater.* **2021,** *4* (12), 14681-14688.

(5) Zheng, Z.; Alawadhi, A. H.; Yaghi, O. M., Green Synthesis and Scale-Up of MOFs for Water Harvesting from Air. *Mol. Front. J.* **2023,** *7* (1), 1-20.

(6) Lenzen, D.; Bendix, P.; Reinsch, H.; Fröhlich, D.; Kummer, H.; Möllers, M.; Hügenell, P. P.; Gläser, R.; Henninger, S.; Stock, N., Scalable green synthesis and full-scale test of the metal–organic framework CAU-10-H for use in adsorption-driven chillers. *Adv. Mater.* **2018,** *30* (6), 1705869.

(7) Kayal, S.; Chakraborty, A.; Teo, H. W. B., Green synthesis and characterization of aluminium fumarate metal-organic framework for heat transformation applications. *Mater. Lett.* **2018,** *221*, 165-167.

(8) Cho, K. H.; Borges, D. D.; Lee, U.; Lee, J. S.; Yoon, J. W.; Cho, S. J.; Park, J.; Lombardo, W.; Moon, D.; Sapienza, A., Rational design of a robust aluminum metal-organic framework for multi-purpose water-sorption-driven heat allocations. *Nat. Commun.* **2020,** *11* (1), 1-8.

(9) Hanikel, N.; Kurandina, D.; Chheda, S.; Zheng, Z.; Rong, Z.; Neumann, S. E.; Sauer, J.; Siepmann, J. I.; Gagliardi, L.; Yaghi, O. M., MOF Linker Extension Strategy for Enhanced Atmospheric Water Harvesting. *ACS Cent. Sci.* **2023,** *9* (3), 551-557.

(10) Jones, D. R.; Schonlau, M.; Welch, W. J., Efficient global optimization of expensive black-box functions. *J. Glob. Optim.* **1998,** *13*, 455-492.

(11) Gongora, A. E.; Xu, B.; Perry, W.; Okoye, C.; Riley, P.; Reyes, K. G.; Morgan, E. F.; Brown, K. A., A Bayesian experimental autonomous researcher for mechanical design. *Sci. Adv.* **2020,** *6* (15), eaaz1708.

(12) Langner, S.; Häse, F.; Perea, J. D.; Stubhan, T.; Hauch, J.; Roch, L. M.; Heumueller, T.; Aspuru-Guzik, A.; Brabec, C. J., Beyond ternary OPV: high-throughput experimentation and self-driving laboratories optimize multicomponent systems. *Adv. Mater.* **2020,** *32* (14), 1907801.

(13) Wahab, H.; Jain, V.; Tyrrell, A. S.; Seas, M. A.; Kotthoff, L.; Johnson, P. A., Machine-learning-assisted fabrication: Bayesian optimization of laser-induced graphene patterning using in-situ Raman analysis. *Carbon* **2020,** *167*, 609-619.

(14) Xie, Y.; Zhang, C.; Deng, H.; Zheng, B.; Su, J.-W.; Shutt, K.; Lin, J., Accelerate synthesis of metal–organic frameworks by a robotic platform and bayesian optimization. *ACS Appl. Mater. Interfaces* **2021,** *13* (45), 53485-53491.

(15) Burger, B.; Maffettone, P. M.; Gusev, V. V.; Aitchison, C. M.; Bai, Y.; Wang, X.; Li, X.; Alston, B. M.; Li, B.; Clowes, R., A mobile robotic chemist. *Nature* **2020,** *583* (7815), 237-241.

(16) Hase, F.; Roch, L. M.; Kreisbeck, C.; Aspuru-Guzik, A., Phoenics: a Bayesian optimizer for chemistry. *ACS Cent. Sci.* **2018,** *4* (9), 1134-1145.

(17) Chevalier, C.; Ginsbourger, D. In *Fast computation of the multi-points expected improvement with applications in batch selection*, International conference on learning and intelligent optimization, Springer: 2013; pp 59-69.
